# New Formulas of Feedback Capacity for AGN Channels with Memory: A Time-Domain Sufficient Statistic Approach †

**DOI:** 10.3390/e27020207

**Published:** 2025-02-15

**Authors:** Charalambos D. Charalambous, Christos Kourtellaris, Stelios Louka

**Affiliations:** Department of Electrical and Computer Engineering, University of Cyprus, P.O. Box 20537, Nicosia CY-1678, Cyprus; kourtellaris.christos@ucy.ac.cy (C.K.);

**Keywords:** feedback capacity, non-stationary, unstable, Gaussian channels, memory, sufficient statistic

## Abstract

Recently, several papers identified technical issues related to equivalent time-domain and frequency-domain “characterization of the *n*–block or transmission” feedback capacity formula and its asymptotic limit, the feedback capacity, of additive Gaussian noise (AGN) channels, first introduce by Cover and Pombra in 1989 (IEEE Transactions on Information Theory). The main objective of this paper is to derive new results on the Cover and Pombra characterization of the *n*–block feedback capacity formula, and to clarify the main points of confusion regarding the time-domain results that appeared in the literature. The first part of this paper derives new equivalent time-domain sequential characterizations of feedback capacity of AGN channels driven by non-stationary and non-ergodic Gaussian noise. It is shown that the optimal channel input processes of the new equivalent sequential characterizations are expressed as functionals of a *sufficient statistic and a Gaussian orthogonal innovations process.* Further, the Cover and Pombra *n*–block capacity formula is expressed as a functional of two generalized matrix difference Riccati equations (DREs) of the filtering theory of Gaussian systems, contrary to results that appeared in the literature and involve only one DRE. It is clarified that prior literature deals with a simpler problem that presupposes the state of the noise is known to the encoder and the decoder. In the second part of this paper, the existence of the asymptotic limit of the *n*–block feedback capacity formula is shown to be equivalent to the convergence properties of solutions of the two generalized DREs. Further, necessary and or sufficient conditions are identified for the existence of asymptotic limits, for stable and unstable Gaussian noise, when the optimal input distributions are asymptotically time-invariant but not necessarily stationary. This paper contains an in-depth analysis, with various examples, and identifies the technical conditions on the feedback code and state space noise realization, so that the time-domain capacity formulas that appeared in the literature, for AGN channels with stationary noises, are indeed correct.

## 1. Introduction, Motivation, Main Results, Current State of Knowledge

In the recent papers [1,2,3], concerns are raised whether the time-domain analysis in [4], deals with the Cover and Pombra [5] “characterization of the *n*–block or transmission” feedback capacity formula and its asymptotic limit, the feedback capacity, of additive Gaussian noise (AGN) channels. Furthermore, the recent comment paper [6] identified gaps in the proof of the simplified frequency-domain characterization of Theorem 4.1 in [4]. The main objective of this paper is to derive new results on the Cover and Pombra characterization of the *n*–block feedback capacity formula, and to clarify the main points of confusion regarding the time-domain results of [4] and related literature i.e., [7].

### 1.1. The Problem, Motivation, and Main Results

We consider the additive Gaussian noise (AGN) channel defined by [5]

(1) $$\begin{array}{c} Y_{t}=X_{t}+V_{t},\;t=1,\ldots ,n,\;\frac{1}{n}E\left\{\sum_{t=1}^{n}{\left(X_{t}\right)}^{2}\right\}\leq \kappa ,\;\kappa \in \left[0,\infty \right) \end{array}$$

where

$X^{n}\overset{▵}{=}\left\{X_{1},X_{2},\ldots ,X_{n}\right\}$ is the sequence of channel input random variables (RVs) $X_{t}:\Omega \to R$;
  $Y^{n}\overset{▵}{=}\left\{Y_{1},Y_{2},\ldots ,Y_{n}\right\}$ is the sequence of channel output RVs $Y_{t}:\Omega \to R$;
  $V^{n}\overset{▵}{=}\left\{V_{1},\ldots ,V_{n}\right\}$ is the sequence of jointly Gaussian distributed RVs $V_{t}:\Omega \to R$, with distribution $P_{V^{n}}\left(dv^{n}\right)$, which are not necessarily stationary or ergodic.
  We wish to examine the feedback capacity of the AGN channel (1) for two distinct formulations of code definition and a noise model, described below under Case (I) and Case (II) formulations. Special cases of these will be related to the existing literature.

**Case (I) Formulation.** This formulation respects the following two conditions.

(I.1). The feedback code does not assume knowledge of the initial state of the noise at the encoder and the decoder (see Definition 1), and
  (I.2) the noise sequence $V^{n}$ is represented by a partially observable (partially observable means that knowledge of $V^{t-1}$ and the initial state do not specify the state $S^{t},t=1,\ldots ,n$.) state space realization, with state sequence $S^{n}$ (see Definition 2).

For a formulation that respects (I.1) and (I.2), Cover and Pombra characterized the “*n*–finite transmission” feedback capacity [5] (Equations (10) and (11)), using the information measure ($C_{n}^{fb}\left(\kappa \right)$ is identified using the converse coding theorem [5]),

(2) $$\begin{array}{c} C_{n}^{fb}\left(\kappa \right)\overset{▵}{=}\underset{P_{X_{t}|X^{t-1},Y^{t-1}},t=1,\ldots ,n:\;\frac{1}{n}E\left\{\sum_{t=1}^{n}{\left(X_{t}\right)}^{2}\right\}\leq \kappa }{\sup}\sum_{t=1}^{n}H\left(Y_{t}|Y^{t-1}\right)-H\left(V^{n}\right) \end{array}$$

provided the supremum exists, where H(·) denotes (differential) entropy.

**Case (I.a) Formulation.** Although not mentioned in [5], if the feedback code assumes knowledge of the initial state of the noise or the channel, $S_{1}=s$, at the encoder and the decoder (see Definition 3), it follows directly from [5] (Equations (10) and (11)), that (2) is replaced by the information measure

(3) $$\begin{array}{c} C_{n}^{fb}\left(\kappa ,s\right)\overset{▵}{=}\underset{P_{X_{t}|X^{t-1},Y^{t-1},s},t=1,\ldots ,n:\;\frac{1}{n}E\left\{\sum_{t=1}^{n}{\left(X_{t}\right)}^{2}|S_{1}=s\right\}\leq \kappa }{\sup}\sum_{t=1}^{n}H\left(Y_{t}|Y^{t-1},s\right)-H\left(V^{n}|s\right). \end{array}$$

**Case (II) Formulation.** This formulation relaxes Conditions (I.1) and (I.2) to the following two conditions:

(II.1) The feedback code assumes knowledge of the initial state of the noise or the channel, $S_{1}=s$, at the encoder and the decoder (see Definition 3);
  (II.2) the noise sequence $V^{n}$ is represented by a fully observable state space realization (fully observable means knowledge of $V^{t-1}$ and initial state specify the state $S^{t},t=1,\ldots ,n$), with state sequence $S^{n}$ such that the noise $V^{t-1}$ and the initial state $S_{1}$ uniquely defines the noise state sequence $S^{t}$ and vice versa for t=1,…,n.
  Thus, Formulation (2), which respects Conditions (I.1) and (I.2), is the most general.

For a formulation that respects Conditions (II.1) and (II.2), Yang, Kavcic, and Tatikonda [8] characterized the *n*–finite transmission feedback capacity [8] (Section II (particularly Section II.C, I–III)), using the information measure,

(4) $$\begin{array}{c} C_{n}^{fb,S}\left(\kappa ,s\right)\overset{▵}{=}\underset{P_{X_{t}|S^{t},Y^{t-1},s},t=1,\ldots ,n:\;\frac{1}{n}E\left\{\sum_{t=1}^{n}{\left(X_{t}\right)}^{2}|S_{1}=s\right\}\leq \kappa }{\sup}\sum_{t=1}^{n}H\left(Y_{t}|Y^{t-1},s\right)-H\left(V^{n}|s\right). \end{array}$$

Compared to $C_{n}^{fb}\left(\kappa \right)$ and $C_{n}^{fb}\left(\kappa ,s\right)$, the definition of $C_{n}^{fb,S}\left(\kappa ,s\right)$ imposes Condition (II.2) and is fundamentally different from the former, because the input distributions of $C_{n}^{fb,S}\left(\kappa ,s\right)$ are different from $C_{n}^{fb}\left(\kappa \right)$ and $C_{n}^{fb}\left(\kappa ,s\right)$. Hence, the information rates of the three formulas are generally different. However, for certain Gaussian noise models of $V^{n}$, it might be the case that, under Condition (II.1), the information measures $C_{n}^{fb}\left(\kappa ,s\right)$ and $C_{n}^{fb,S}\left(\kappa ,s\right)$ coincide. We provide several examples in the main body of this paper.

**Motivation and Fundamental Differences of Case (I) and Case (II) Formulations.**

At this point, we pause to discuss two technical issues of Case (I) and Case (II) formulations, which are not clarified in [4,7,9]. These technical issues are related to the time-domain characterization of feedback capacity, Theorem 6.1 in [4]; they are first discussed in [1,2,3,10]. A recent comment paper [6] also identified gaps in the proof of the frequency-domain characterization of capacity, Theorem 4.1 in [4], which affect the proofs of Theorems 4.1, 4.6, 5.3 and 6.1; Propositions 4.7 and 5.1; Remarks 4.5 and 5.2; and Lemma 6.1 in [4].

To illustrate the technical issues, we consider the autoregressive moving average stable noise denoted by ARMA (a,c),a∈[−1,1],c∈(−1,1),c≠a, studied by many authors [4,7,8,9,11,12], as a benchmark example.

(5) $$\begin{array}{cc} \; & V_{t}=cV_{t-1}+W_{t}-aW_{t-1},\;\forall t\in Z_{+}\overset{▵}{=}\left\{1,2,\ldots \right\}, \end{array}$$

(6) $$\begin{array}{cc} \; & V_{0}\in N\left(0,K_{V_{0}}\right),\;K_{V_{0}}\geq 0,\;W_{0}\in N\left(0,K_{W_{0}}\right),\;K_{W_{0}}\geq 0,W_{t}\in N\left(0,K_{W}\right),\;K_{W}>0,\;\forall t, \end{array}$$

(7) $$\begin{array}{cc} \; & \left\{W_{0},W_{1},\ldots ,W_{n}\right\}\text{indep. seq. and indep. of}\;V_{0}. \end{array}$$

As in [4,7], we define the state variable of the noise by

(8) $$\begin{array}{c} S_{t}\overset{▵}{=}\frac{cV_{t-1}-aW_{t-1}}{c-a},\;\forall t\in Z_{+} \end{array}$$

Then, the state space realization of $V^{n}$ is

(9) $$\begin{array}{cc} \; & S_{t+1}=cS_{t}+W_{t},\;V_{t}=\left(c-a\right)S_{t}+W_{t},\;\forall t\in Z_{+}, \end{array}$$

(10) $$\begin{array}{cc} \; & K_{S_{1}}=\frac{{\left(c\right)}^{2}K_{V_{0}}+{\left(a\right)}^{2}K_{W_{0}}}{{\left(c-a\right)}^{2}},\;K_{V_{0}}\geq 0,\;K_{W_{0}}\geq 0\;\text{both given}. \end{array}$$

Bounds on feedback capacity, when a=0, i.e., corresponding to an autoregressive noise are derived in [13,14,15] using linear coding schemes.

For the Case (I) formulation, the information measure $C_{n}^{fb}\left(\kappa \right)$, i.e., (2), corresponds to the supremum over channel input distributions $P_{X_{t}|X^{t-1},Y^{t-1}},t=1,\ldots ,n$.

For the Case (II) formulation, the information measure $C_{n}^{fb,S}\left(\kappa ,s\right)$, i.e., (4), corresponds to the supremum over channel input distributions $P_{X_{t}|S^{t},Y^{t-1},s},t=1,\ldots ,n$, and Conditions (II.1) and (II.2) are necessary. Alternatively, the necessary conditions for $C_{n}^{fb}\left(\kappa \right)$ to reduce to $C_{n}^{fb,S}\left(\kappa ,s\right)$ are the conditions stated in (12) and (13) (these also follow independently of the Case (I) formulation from the converse coding theorem).

(11) $$\begin{array}{cc} \; & P_{X_{t}|X^{t-1},Y^{t-1}}=P_{X_{t}|V^{t-1},Y^{t-1}}\;\text{holds by channel def.}\;Y_{k}=X_{k}+V_{k},k=1,\ldots ,n; \end{array}$$

(12) $$\begin{array}{cc} \; & =P_{X_{t}|V^{t-1},Y^{t-1},s}\;ifS_{1}=s\;\text{is known to the feedback code, i.e., Cond. (II.1)}; \end{array}$$

(13) $$\begin{array}{cc} \; & =P_{X_{t}|S^{t},Y^{t-1},s}\;if\left(V^{t-1},S_{1}=s\right)\text{uniquely}\;\mathrm{def}.S^{t}\;\text{and vice-versa, i.e., Cond. (II.2)}. \end{array}$$

Thus, a necessary condition for (13) to hold is $S_{1}=\left(V_{0},W_{0}\right)=\left(v_{0},w_{0}\right)=s$, which is known to the encoder. It follows from [4] (Theorem 6.1; also see Lemma 6.1 and the comments above it, Equation (71)), that Conditions (II.1) and (II.2) are assumed; hence, these results are not developed for the Cover and Pombra [5] formulation. Additional information can be found in Remark 5.

Second, the analysis of the asymptotic per unit time limits of (2)–(4), i.e.,

(14) $$\begin{array}{cc} C^{fb}\left(\kappa \right)\overset{▵}{=} & \lim_{n⟶\infty }\underset{{\left\{P_{X_{t}|X^{t-1},Y^{t-1}}\right\}}_{t=1}^{n},\;\frac{1}{n}E\left\{\sum_{t=1}^{n}{\left(X_{t}\right)}^{2}\right\}\leq \kappa }{\sup}\frac{1}{n}\left\{\sum_{t=1}^{n}H\left(Y_{t}|Y^{t-1}\right)-H\left(V^{n}\right)\right\}, \end{array}$$

is a non-trivial problem, and it requires certain technical necessary and/or sufficient conditions for the limits to exist, as well as for the rates to be independent of the initial distribution $P_{V_{1}}$ or the initial data, $S_{1}=s$ (see [3,10]), even if the noise process $V^{n}$ is stationary. It is easy to verify that the analysis in the past studies [4,7,9,11,12] considered the simpler problem $C_{n}^{fb,S}\left(\kappa ,s\right)$, and that the asymptotic limit $\lim_{n⟶\infty }\frac{1}{n}C_{n}^{fb,S}\left(\kappa ,s\right)$ does not correspond to the ergodic capacity. We clarify these points in our examples.

**Main Results.** The main results of this paper are briefly stated below.

(1) In the first part of this paper, we derive new equivalent sequential characterizations of the Cover and Pombra “*n*–block or transmission” feedback capacity formula [5] (Equation (11)), $C_{n}^{fb}\left(\kappa \right)$ (this first appeared in [10]). In particular, we derive equivalent realizations to the optimal channel input process $X^{n}$ [5] (Equation (11)), which are linear functionals of a *finite-dimensional sufficient statistic and an orthogonal innovations process*. From these new realizations follows the equivalent sequential characterizations of the “*n*–block or transmission” feedback capacity formula [5] (Equation (11)), which will henceforth be called the “*n*–finite transmission feedback information (*n*–FTFI) capacity”. The new *n*–FTFI capacity is expressed as a functional of *two generalized matrix difference Riccati equations (DREs) of the filtering theory of Gaussian systems*, instead of the one DRE given in [4,7,8]. In fact, we also show that the *n*–FTFI capacity of [4,7,8] corresponds to $C_{n}^{fb,S}\left(\kappa ,s\right)$.

(2) In the second part of this paper, we analyze the asymptotic per unit time limit of the sequential characterizations of the *n*–FTFI capacity, when the supremum and limit over n⟶∞ are interchanged in (14), denoted by $C^{fb,o}\left(\kappa \right)$. Then, we show $C^{fb,o}\left(\kappa \right)=C^{fb}\left(\kappa \right)$. We identify necessary and/or sufficient conditions for the asymptotic limit to exist, and for the optimal input process $X_{t},t=1,\ldots ,$ to be asymptotically stationary, in terms of the convergence properties of two generalized matrix difference Riccati equations (DREs) to their corresponding two generalized matrix algebraic Riccati equations (AREs). We make use of the so-called detectability, stabilizability, and unit circle controllablity conditions of generalized Kalman filters of Gaussian processes [16,17].

(3) From (1) and (2), we derive analogous results for $C_{n}^{fb}\left(\kappa ,s\right)$ and its per unit time asymptotic limit, denoted by $C^{fb,o}\left(\kappa ,s\right)$, as degenerate versions of $C_{n}^{fb}\left(\kappa \right)$ and $C^{fb,o}\left(\kappa \right)$. Further, we show that for certain noise models, and under certain conditions, it holds that $C^{fb,o}\left(\kappa ,s\right)=C^{fb}\left(\kappa \right)$, i.e., these values do not depend on the initial state or initial distributions.

(4) From (1) and (2), we derive analogous results for the Case (II) formulation, i.e., $C_{n}^{fb,S}\left(\kappa ,s\right)$ and its per unit time asymptotic limit denoted by $C^{fb,S}\left(\kappa ,s\right)$, and we show these are fundamentally different from the Case (I) formulation, $C_{n}^{fb}\left(\kappa \right)$ and $C^{fb}\left(\kappa \right)$, as well as $C_{n}^{fb}\left(\kappa ,s\right)$ and $C^{fb}\left(\kappa ,s\right)$. In particular, we show that the characterizations of *n*–FTFI capacity, $C_{n}^{fb,S}\left(\kappa ,s\right)$, for the Case (II) formulation follow directly from the Case (I) formulation, as a special case (an independent derivation is also presented). Moreover, $C_{n}^{fb,S}\left(\kappa ,s\right)$ is a functional of one generalized DRE, while $C_{n}^{fb}\left(\kappa \right),C_{n}^{fb}\left(\kappa ,s\right)$ are functionals of two generalized DREs.

### 1.2. The Code Definitions and Noise Models

**Case (I) Feedback Code and Noise Definitions.** For the Case (I) formulation, we consider the code of Definition 1 (due to [5]).

**Definition 1.**  *Time-varying feedback code [5]*

*A noiseless time-varying feedback code for the AGN Channel (1) is denoted by $\left(2^{nR},n\right)$, n=1,2,… and consists of the following elements and assumptions:*
*(i) The uniformly distributed messages $W:\Omega \to M_{n}\overset{▵}{=}\left\{1,2,\ldots ,2^{nR}\right\}$.*
*(ii) The time-varying encoder strategies, often called codewords of block length n, defined by (the superscript e(·) on $E^{e}$ indicates that the distribution depends on the strategy $e\left(\cdot \right)\in E_{\left[1,n\right]}\left(\kappa \right)$).*
(15) $$\begin{array}{cc} E_{\left[1,n\right]}\left(\kappa \right)≜ & \{X_{1}=e_{1}\left(W\right),X_{2}=e_{2}\left(W,X_{1},Y_{1}\right)\ldots ,X_{n}=e_{n}\left(W,X^{n-1},Y^{n-1}\right):\\ \; & \;\frac{1}{n}E^{e}\left(\sum_{t=1}^{n}{\left(X_{t}\right)}^{2}\right)\leq \kappa \}. \end{array}$$
  *(iii) The average error probability of the decoder functions $y^{n}⟼d_{n}\left(y^{n}\right)\in M_{n}$, defined by*
(16) $$\begin{array}{c} P_{error}^{\left(n\right)}=P\left\{d_{n}\left(Y^{n}\right)\neq W\right\}=\frac{1}{2^{nR}}\sum_{w=1}^{2^{nR}}P\left\{d_{n}\left(Y^{n}\right)\neq w|W=w\right\}. \end{array}$$
  *(iv) The channel input sequence “$X^{n}\overset{▵}{=}\left\{X_{1},\ldots ,X_{n}\right\}$ is causally related (a notion found in [5], page 39, above Lemma 5) to $V^{n}$”, which is equivalent to the following decomposition of the joint probability distribution of $\left(X^{n},V^{n}\right)$:*
(17) $$\begin{array}{cc} P_{X^{n},V^{n}}= & P_{V_{n}|V^{n-1},X^{n}}\;P_{X_{n}|X^{n-1},V^{n-1}}\;\ldots \;P_{V_{2}|V_{1},X^{2}}P_{X_{2}|X_{1},V_{1}}P_{V_{1}|X_{1}}P_{X_{1}} \end{array}$$
(18) $$\begin{array}{cc} = & P_{V^{n}}\prod_{t=1}^{n}P_{X_{t}|X^{t-1},V^{t-1}},\;that\;is,\;P_{V_{t}|V^{t-1},X^{t}}=P_{V_{t}|V^{t-1}}. \end{array}$$
  *That is, $X^{t}↔V^{t-1}↔V_{t}$ is a Markov chain, for t=1,…,n. As usual, the messages W are independent of the channel noise $V^{n}$.*
*A rate R is called an achievable rate with feedback coding, if there exists a sequence of codes $(2^{nR},n),n=1,2,\ldots$, such that $P_{error}^{\left(n\right)}⟶0$ as n⟶∞. The feedback capacity $C^{fb}\left(\kappa \right)$ is defined as the supremum of all achievable rates R.*

*We note that, in general, $C^{fb}\left(\kappa \right)$ depends on the initial distribution $P_{V_{1}}$; the ergodic capacity requires that $C^{fb}\left(\kappa \right)$ is independent of $P_{V_{1}}$ (see [18]).*

We consider a noise model which is consistent with [5], i.e., $V^{n}$ is jointly Gaussian distributed, $P_{V^{n}}=\times_{t=1}^{n}P_{V_{t}|V^{t-1}}$, and induced by the partially observable state space (PO-SS) realization of Definition 2.

**Definition 2.**  *A time-varying PO-SS realization of Gaussian noise $V^{n}\in N\left(0,K_{V^{n}}\right)$ is defined by*

(19) $$\begin{array}{cc} \; & S_{t+1}=A_{t}S_{t}+B_{t}W_{t},\;t=1,\ldots ,n-1 \end{array}$$
(20) $$\begin{array}{cc} \; & V_{t}=C_{t}S_{t}+N_{t}W_{t},\;t=1,\ldots ,n, \end{array}$$
(21) $$\begin{array}{cc} \; & S_{1}\in N\left(\mu_{S_{1}},K_{S_{1}}\right),\;K_{S_{1}}⪰0, \end{array}$$
(22) $$\begin{array}{cc} \; & W_{t}\in N\left(0,K_{W_{t}}\right),\;K_{W_{t}}⪰0,\;t=1\ldots ,n\;indep.Gaussian\;process,\;W^{t}\;indep.of\;S_{1}, \end{array}$$
(23) $$\begin{array}{cc} \; & S_{t}:\Omega \to R^{n_{s}},\;W_{t}:\Omega \to R^{n_{w}},\;V_{t}:\Omega \to R^{n_{v}},\;R_{t}\overset{▵}{=}N_{t}K_{W_{t}}N_{t}^{T}≻0,\;t=1,\ldots ,n \end{array}$$
  *where $n_{v}=1$, $n_{s},n_{w}$ are arbitrary positive integers, $\left(A_{t},B_{t},C_{t},N_{t},\mu_{S_{1}},K_{S_{1}},K_{W_{t}}\right)$ are nonrandom, measurable in t, with bounded entries, and $n_{s},n_{w}$ are finite positive integers.*
*The above components $B_{t}W_{t}$ and $N_{t}W_{t}$ can be taken as*
(24) $$\begin{array}{cc} \; & B_{t}W_{t}=B_{t}^{1}W_{t}^{1}+B_{t}^{2}W_{t}^{2},\;N_{t}W_{t}=N_{t}^{1}W_{t}^{1}+N_{t}^{2}W_{t}^{2},\;t=1,\ldots ,n, \end{array}$$
(25) $$\begin{array}{cc} \; & W^{1,n}\;and\;W^{1,n}\;areindependent, \end{array}$$
  *where $B_{t}^{1}$ or $B_{t}^{2}$ can be zero and $N_{t}^{1}$ or $N_{t}^{2}$ can be zero ∀t.*
*A time-invariant PO-SS realization of the Gaussian noise $V^{n}\in N\left(0,K_{V^{n}}\right)$ is defined by (19)–(23), with $\left(A_{t},B_{t},C_{t},N_{t},K_{W_{t}}\right)=\left(A,B,C,N,K_{W}\right),\forall t$.*

For the Case (I) formulation, we use the terminology “partially observable”, which is standard in filtering theory [16], because the noise $V^{n}$ induces a distribution $P_{V^{n}}=\times_{t=1}^{n}P_{V_{t}|V^{t-1}}$, and $P_{V_{t}|V^{t-1}}$ cannot be expressed as a function of the state of the noise, i.e., $V^{t-1}$ does not uniquely define $S^{t}$. However, if $S_{1}=s$ is known to the encoder, then it can be as easily verified from the ARMA (a,c), that $V^{t-1}$ uniquely defines $S^{t}$, recursively. However, for the PO-SS realization, with $B_{t}^{2}=0,N_{t}^{1}=0,\forall t$, even if the initial state $S_{1}=s$ is known to the encoder, $V^{t-1}$ does not uniquely define $S^{t}$ because there are two independent noises $W^{1,n-1}$ and $W^{2,n}$ that enter the equations of $S^{n}$ and $V^{n}$. The PO-SS realization is often adopted in many practical problems of engineering and science to realize jointly Gaussian processes $V^{n}$.

We should emphasize that for the Case (I) formulation to be consistent with the Cover and Pombra [5] formulation, both the code of Definition 1 and the PO-SS realization of Definition 2 must respect the following two conditions:

**(A1)** *The initial state $S_{1}$ of the noise is not known at the encoder nor the decoder;*

**(A2)** *At each t, the representation of the noise $V^{t-1}$ by the PO-SS realization of Definition 2 does not uniquely determine the state of the noise $S^{t}$ and vice-versa, i.e., it is a partially observable realization.*

**Case (II) Formulation of Feedback Code and Noise Definitions.** For the Case (II) formulation, we presuppose the following:

**Condition 1.** *The initial state of the noise or channel $S_{1}=s$ is known to the encoder and decoder;*

**Condition 2.** *Given a fixed initial state $S_{1}=s$, known to the encoder and the decoder, at each t, the channel noise $V^{t-1}$ uniquely defines the state of the noise $S^{t}$ and vice-versa.*

Thus, for the Case (II) formulation, the code is that of Definition 3, below (hence different from Definition 1).

**Definition 3.**  *A code with initial state known at the encoder and the decoder*

*A variant of the code of Definition 1 is feedback code with the initial state of the noise or channel $S_{1}=s$, known to the encoder and decoder strategies, denoted by $\left(s,2^{nR},n\right)$, n=1,2,….*
*The code $\left(s,2^{nR},n\right)$, n=1,2,… is defined as in Definition 2, with (ii), (iii), and (iv) being replaced by*
$$\begin{array}{cc} E_{\left[1,n\right]}^{s}\left(\kappa \right)≜ & \{X_{1}=e_{1}\left(W,S_{1}\right),X_{2}=e_{2}\left(W,S_{1},X_{1},Y_{1}\right)\ldots ,X_{n}=e_{n}\left(W,S_{1},X^{n-1},Y^{n-1}\right): \end{array}$$
(26) $$\begin{array}{cc} \; & \frac{1}{n}E^{e}\left\{\sum_{i=1}^{n}{\left(X_{t}\right)}^{2}|S_{1}=s\right\}\leq \kappa \},\;\left(y^{n},s\right)⟼d_{n}^{s}\left(y^{n},s\right)\in M_{n}, \end{array}$$
(27) $$\begin{array}{cc} P_{X^{n},V^{n}|S_{1}}= & P_{V^{n}|S_{1}}\prod_{t=1}^{n}P_{X_{t}|X^{t-1},V^{t-1},S_{1}},\;that\;is,\;P_{V_{t}|V^{t-1},X^{t},S_{1}}=P_{V_{t}|V^{t-1},S_{1}}. \end{array}$$
  *The feedback capacity is denoted by $C^{fb}\left(\kappa ,s\right)$ and should be distinguished from $C^{fb}\left(\kappa \right)$.*
*The feedback capacity is denoted by and $C^{fb,S}\left(\kappa ,s\right)$ if, in addition, Condition 2 holds.*
*The initial state may include $S_{1}\overset{▵}{=}\left(V_{-\infty }^{0},Y_{-\infty }^{0}\right)$, etc.*

It will become apparent that past studies [4,7,8] considered feedback capacity, $C^{fb,S}\left(\kappa ,s\right)$, and not $C^{fb}\left(\kappa ,s\right)$ or $C^{fb}\left(\kappa \right)$.

For the Case (II) formulation, it is obvious (from the converse to the coding theorem) that the optimal channel input conditional distribution is expressed as a function of the state of the noise, $S^{n}$, due to (12), (13).

### 1.3. Approach of This Paper

Our approach and analysis of information measures (2)–(4), as well sa their per unit time limits, is based on the following two step procedure:

**Step # 1.** We apply a linear transformation to the Cover and Pombra optimal channel input process [5] (Equation (11)), (see (33)–(39) to equivalently represent it by a linear functional of the past channel noise sequence, the past channel output sequence, and an orthogonal Gaussian process, i.e., an innovations process. That is, $X^{n}$ is uniquely represented, since it is expressed in terms of the orthogonal process.

**Step # 2.** We express the optimal input process by a functional of a *sufficient statistic*, which satisfies a Markov recursion, and an *orthogonal innovations process*. It then follows that the Cover and Pombra characterization of the “*n*–block” formula [5] (Equation (10)) (see (33) and (34)) is equivalently represented by a sequential characterization. The problem of feedback capacity is then expressed as the maximization over two sequences of time-varying strategies of the channel input process of the difference of (differential) entropies of the innovations processes of $Y^{n}$ and $V^{n}$ (analog of entropies in the right-hand side of (2)).

(28) $$\begin{array}{cc} H\left(Y^{n}\right)-H\left(V^{n}\right)= & \sum_{t=1}^{n}\left\{H\left(Y_{t}|Y^{t-1}\right)-H\left(V_{t}|V^{t-1}\right)\right\} \end{array}$$

(29) $$\begin{array}{cc} = & \sum_{t=1}^{n}\left\{H\left(Y_{t}-E\left\{Y_{t}|Y^{t-1}\right\}|Y^{t-1}\right)-H\left(V_{t}-E\left\{V_{t}|V^{t-1}\right\}|V^{t-1}\right)\right\} \end{array}$$

(30) $$\begin{array}{cc} \overset{\left(a\right)}{=} & \sum_{t=1}^{n}\left\{H\left(Y_{t}-E\left\{Y_{t}|Y^{t-1}\right\}\right)-H\left(V_{t}-E\left\{V_{t}|V^{t-1}\right\}\right)\right\} \end{array}$$

(31) $$\begin{array}{cc} = & \sum_{t=1}^{n}\left\{H\left(I_{t}\right)-H\left({\hat{I}}_{t}\right)\right\},\;I_{t}\overset{▵}{=}Y_{t}-E\left\{Y_{t}|Y^{t-1}\right\},\;{\hat{I}}_{t}\overset{▵}{=}V_{t}-E\left\{V_{t}|V^{t-1}\right\} \end{array}$$

where (a) is due to the Gaussianity of $Y^{n}$ and $V^{n}$, as well as due the independence of the innovations processes $I_{t}$ and $Y^{t-1}$ and of innovations processes ${\hat{I}}_{t}$ and $V^{t-1}$.

The asymptotic analysis of $\lim_{n⟶\infty }\frac{1}{n}C_{n}^{fb}\left(\kappa \right)$ (or with limit and supremum interchanged) is then addressed from the asymptotic properties of entropy rates and the average power,

(32) $$\begin{array}{c} \lim_{n⟶\infty }\frac{1}{n}\sum_{t=1}^{n}\left\{H\left(I_{t}\right)-H\left({\hat{I}}_{t}\right)\right\},\;\lim_{n⟶\infty }\frac{1}{n}E\left\{\sum_{t=1}^{n}{\left(X_{t}\right)}^{2}\right\} \end{array}$$

over the channel input distributions, and where the covariance of the innovations process of $Y^{n}$ is a functional of the solutions of two generalized matrix DREs. We identify necessary and/or sufficient conditions for the existence of limits, irrespective of whether the noise $V^{n}$ is non-stationary, unstable, or stationary. Further, we show that, in general, the characterizations of feedback capacity for the Case (I) and Case (II) formulations are fundamentally different, and we identify conditions based on the feedback codes and noise to coincide.

### 1.4. Review of Related Literature

Asymptotic feedback capacity formulas and bounds for AGN channels, driven by stationary and asymptotically stationary (often limited memory) noise, are derived since the early 1970s in an anthology of papers based on information theoretic formulas, under various assumptions [7,8,9,11,12,13,14,19,20,21,22,23,24,25,26,27,28,29,30,31,32,33], in two directions.

**(D1)**  Characterizations and explicit formulas of asymptotic feedback capacity that correspond to feedback codes, when the initial state of the noise (the initial state $S_{1}$ is any a priori information) $S_{1}=s_{1}$ is known or not known to the encoder and the decoder;
**(D2)**  Bounds on asymptotic feedback capacity that correspond to linear feedback coding schemes of communicating Gaussian random variables (RVs), Θ:Ω→R, and coding schemes of communicating digital messages $W:\Omega \to \{1,2,\ldots ,2^{nR}\}$, when the initial state the $S_{1}=s_{1}$ is known or not known to the encoder and the decoder.

#### 1.4.1. The Cover and Pombra Characterizations of Capacity Pombra [5]

Cover and Pombra characterized the *n*–FTFI capacity for non-stationary and non-ergodic noise $V^{n}$, [5] (Equation (10)), by (we use H(X) to denote differential entropy of a continuous-valued RV *X*; hence, we indirectly assume the probability density functions exist)

(33) $$\begin{array}{cc} C_{n}^{fb}\left(\kappa \right)\overset{▵}{=} & \max_{\left(B^{n},K_{{\bar{Z}}^{n}}\right):\;\frac{1}{n}tr\left\{E\left(X^{n}{\left(X^{n}\right)}^{T}\right)\right\}\leq \kappa }H\left(Y^{n}\right)-H\left(V^{n}\right) \end{array}$$

(34) $$\begin{array}{cc} = & \max_{\left(B^{n},K_{{\bar{Z}}^{n}}\right):\;\frac{1}{n}tr\left\{B^{n}\;K_{V^{n}}\;{\left(B^{n}\right)}^{T}+K_{{\bar{Z}}^{n}}\right\}\leq \kappa }\frac{1}{2}\log\frac{|\left(B^{n}+I_{n\times n}\right)K_{V^{n}}{\left(B^{n}+I_{n\times n}\right)}^{T}+K_{{\bar{Z}}^{n}}|}{|K_{V^{n}}|} \end{array}$$

where the distribution $P_{Y^{n}}$ is induced by a jointly Gaussian channel input process $X^{n}$ [5] (Equation (11)):

(35) $$\begin{array}{cc} \; & X_{t}=\sum_{j=1}^{t-1}B_{t,j}V_{j}+{\bar{Z}}_{t},\;t=1,\ldots ,n, \end{array}$$

(36) $$\begin{array}{cc} \; & X^{n}=B^{n}V^{n}+{\bar{Z}}^{n},\;Y^{n}=\left(B^{n}+I_{n\times n}\right)V^{n}+{\bar{Z}}^{n}, \end{array}$$

(37) $$\begin{array}{cc} \; & {\bar{Z}}^{n}\;\text{is jointly Gaussian},\;N(0,K_{{\bar{Z}}^{n}}),\;{\bar{Z}}^{n}\;\text{is independent of}\;V^{n}, \end{array}$$

(38) $$\begin{array}{cc} \; & X^{n}\overset{▵}{=}{\left[\begin{array}{cccc} X_{1} & X_{2} & \ldots & X_{n} \end{array}\right]}^{T}\;\text{similarly for the rest},\;B^{n}\;\text{lower diagonal matrix}, \end{array}$$

(39) $$\begin{array}{cc} \; & \frac{1}{n}E\left\{\sum_{t=1}^{n}{\left(X_{t}\right)}^{2}\right\}=\frac{1}{n}tr\;\left\{E\left(X^{n}{\left(X^{n}\right)}^{T}\right)\right\}\leq \kappa . \end{array}$$

The notation $N(0,K_{{\bar{Z}}^{n}})$ means that the random variable ${\bar{Z}}^{n}$ is jointly Gaussian with mean $E\{{\bar{Z}}^{n}\}=0$ and covariance matrix $K_{{\bar{Z}}^{n}}=E\left\{{\bar{Z}}^{n}{\left({\bar{Z}}^{n}\right)}^{T}\right\}$, and $I_{n\times n}$ denotes an *n* by the *n* identity matrix. Feedback capacity, $C^{fb}\left(\kappa \right)$, is characterized by the per unit time limit of the *n*–FTFI capacity [5] (Theorem 1).

(40) $$\begin{array}{c} C^{fb}\left(\kappa \right)\overset{▵}{=}\lim_{n⟶\infty }\frac{1}{n}C_{n}^{fb}\left(\kappa \right). \end{array}$$

Over the years, considerable efforts have been devoted to compute $C_{n}^{fb}\left(\kappa \right)$ and $C^{fb}\left(\kappa \right)$ [4,7,8,9,11,33], often under simplified assumptions on the channel noise. In addition, bounds are described in [27,28,29], while numerical methods are developed in [31] for time-invariant AGN channels, driven by stationary noise. In [4,7,11,33,34], the authors considered a variant of (40) by interchanging the per unit time limit and maximization operations under the following assumption: the joint process $(X^{n},Y^{n}),n=1,2,\ldots$ is either *jointly stationary or asymptotically stationary*, and *the joint distribution of the joint process $(X^{n},Y^{n}),n=1,2,\ldots$ is time-invariant*. We describe [4,7,8] below.

#### 1.4.2. The Yang, Kavcic and Tatikonda [8] Characterization of Maximal Information Rate

In [8], the authors analyzed the feedback capacity of the AGN channel (1), driven by a stationary noise, described the power spectral density (PSD) functions $S_{V}\left(e^{j\theta }\right),\theta \in \left[-\pi ,\pi \right]$:

(41) $$\begin{array}{c} S_{V}\left(e^{j\theta }\right)\overset{▵}{=}K_{W}\frac{\left(1-\sum_{k=1}^{L}a\left(k\right)e^{jk\theta }\right)\left(1-\sum_{k=1}^{L}a\left(k\right)e^{-jk\theta }\right)}{\left(1-\sum_{k=1}^{L}c\left(k\right)e^{jk\theta }\right)\left(1-\sum_{k=1}^{L}c\left(k\right)e^{-jk\theta }\right)},\;|c\left(k\right)|<1,\;|a\left(k\right)|<1,\;c\left(k\right)\neq a\left(k\right). \end{array}$$

The analysis in [8] is based on time-domain methods and corresponds to the Case (II) formulation (see [8] (Section II; in particular, Section II.C, I–III, Theorem 1, Section III) by considering a specific time-invariant, stable, state space realization of the noise PSD (41), such that Conditions (II.1), and (II.2) hold, i.e.,

*The initial state of the noise, $S_{1}=s$, is known to the encoder and the decoder, and the initial state and noise $\left(s,V^{t-1}\right)$ uniquely define the noise state $S^{t}$, and vice-versa, for all t.*

The time-domain characterization of feedback capacity, called the maximal information rate [8] (Theorem 6), corresponds to the supremum and limit being interchanged, and involves only one matrix Riccati equation of the linear filtering theory. However, ref. [8] (Theorem 6) does not state the conditions based on which the maximal information rate is valid (i.e., existence of asymptotic limit).

#### 1.4.3. The Kim [4] Characterization of Feedback Capacity

The author in [4] also analyzed the feedback capacity of the AGN channel (1), driven by stationary noise described by the PSD (41) and by a time-invariant, stable, state space realization of the noise $V^{n}$ (see [4] (Section VI)). A major point of confusion is that the characterization of feedback capacity in the time domain [4] (Theorem 6.1) does not state whether this corresponds to Case (I) or Case (II) formulations. The reader, however, can verify from [4] (Lemma 6.1 and comments above it) that the time-domain characterization of feedback capacity [4] (Theorem 6.1) corresponds to the Case (II) formulation, as stated in the study by Yang, Kavcic and Tatikonda [8]. In fact, the characterization of feedback capacity [4] (Theorem 6.1) involves only one Riccati equation of the linear filtering theory, as in [8]. We reconfirm this point at various parts of this paper (see, for example, Section 2.6).

#### 1.4.4. The Gattami [7] Characterization of Feedback Capacity and Semi-Definite Progamming Formulation

The authors in [7] re-visited the feedback capacity of the AGN channel (1) driven by a stationary noise described by the PSD (41) and with a time-invariant, stable, state space realization of the noise $V^{n}$. One of the main results of [7] is the feedback capacity characterization for the Case (II) formulation, i.e., that involves only one matrix Riccati equation of the filtering theory, precisely as in [4,8]. Another main result of [7] is the re-formulation of the optimization problem using semi-definite programming.

In the following remark, we will provide additional insights into the results of [4,7,8].

**Remark 1.**  *On the formulas in [4,7,8].*

*Refs. [4,7] considered the stable, time-invariant PO-SS realization of Definition 2, with $W_{t}:\Omega \to R,N_{t}=1,\forall t$, i.e., $n_{w}=1$.*

*In [4], (Theorem 6.1) (see [4] (above and below Equation (70))), the asymptotic characterization of feedback capacity involves one filtering matrix ARE and is achieved by a channel input, which is different at times $n=1,2,\ldots ,n_{s}$ from subsequent time $n=n_{s}+1,\ldots$, given by*

(42) $$\begin{array}{cc} \; & X_{n}=Z_{n}\in N\left(0,K_{Z}\right),K_{Z}>0,\;n=1,\ldots ,n_{s}, \end{array}$$

(43) $$\begin{array}{cc} \; & X_{n}=\chi \left(S_{n}-E\left\{S_{n}|Y^{n-1}\right\}\right),\;n=n_{s}+1,n_{s}+2,\ldots . \end{array}$$

*where χ is a constant vector, and $Z_{n},n=1,2,\ldots ,n_{s}$ are IID Gaussian RVs.*

*In [7] (Theorem 3), the asymptotic characterization of feedback capacity involves one filtering matrix ARE and is incurred by the time-invariant channel input*

(44) $$\begin{array}{c} X_{n}=\chi \left(S_{n}-E\left\{S_{n}|Y^{n-1}\right\}\right)+Z_{n},\;Z_{n}\in N\left(0,K_{Z}\right),\;K_{Z}\geq 0,\;n=1,2,\ldots \end{array}$$

*$Z_{n},n=1,2,\ldots$, are IID Gaussian RVs.*

*In [8], a state space realization of the PSD is considered, and the maximal information rate is presented, (Equation (125)) and [8] (Theorem 6, Equation (138), $I_{max}$), which involves one filtering matrix ARE. It is achieved by*

(45) $$\begin{array}{cc} \; & X_{n}=\chi \left(S_{n-1}-E\left\{S_{n-1}|Y^{n-1},S_{0}=s_{0}\right\}\right)+Z_{n},\;X_{1}=Z_{1}, \end{array}$$

(46) $$\begin{array}{cc} \; & Z_{n}\in N\left(0,K_{Z}\right),\;K_{Z}\geq 0,\;n=1,2,\ldots , \end{array}$$

*where $Z_{n},n=1,2,\ldots$ are IID Gaussian RVs.*

*The above references computed the feedback capacity and maximal information rate for the ARMA (a,c),a∈[−1,1],c∈(−1,1),c≠a and arrived at the conclusion that it is precisely Butman’s [13] and Wolfowitz’s [14] lower bound.*

*It will become apparent that [4,7,8] arrived at the above expressions by considering problem $C^{fb,S}\left(\kappa ,s\right)$, and not $C^{fb}\left(\kappa \right)$ or $C^{fb}\left(\kappa ,s\right)$.*

Sequential equivalent characterizations of the Cover and Pombra [5] *n*–FTFI capacity, $C_{n}^{fb}\left(\kappa \right)$ and $C^{fb}\left(\kappa \right)$ capacity of the AGN channel (1) driven by a non-stationary and non-ergodic Gaussian noise $V^{n}$, as well as by time-varying, unstable (and stable) state space realizations of Definition 2, are derived in [1,2,3,10], including relations between Case (I) and Case (II) formulations. In particular, ref. [10] proved that $C_{n}^{fb}\left(\kappa \right)$ of the AGN channel with state space noise of Definition 2 involves two matrix Riccati equations of the linear filtering theory and that, under certain conditions, $C_{n}^{fb}\left(\kappa \right)$ reduces to $C_{n}^{fb,S}\left(\kappa ,s\right)$, which involves only one matrix Riccati equation. Corresponding expressions are obtained for their per unit time asymptotic limits. The methods and results of [10] are generalized to multiple-input multiple-output (MIMO) Gaussian channels in [3]. Further, ref. [2] derived closed-form expressions of $C^{fb}\left(\kappa \right)$ for AGN channels driven by the stable and unstable ARMA (a,c),a∈(−∞,∞),c∈(−∞,∞),c≠a noise, showing the connection between Case (I) and Case (II) formulations. Ref. [3] generalized the earlier investigation in [1], which considered the autoregressive unit memory stable and unstable noise. An investigation of nonfeedback capacity of stable and unstable noise is outlined in [35]. The connection of ergodic theory and feedback capacity of unstable channels is discussed in [36,37].

MIMO Gaussian channels are also investigated in [38]. However, many of the expressions in [38] are previously obtained in [3,10]. The analysis in [38] does not include a derivation of the optimal channel input that achieves $C_{n}^{fb}\left(\kappa \right)$ and $C^{fb}\left(\kappa \right)$, and does not discuss the connection between Case (I) and Case (II) formulations. The closed-form expressions of capacity of the examples in [38], are special cases of expressions in [1] and in [2], which treated the noise ARMA (a,c),∀a∈(−∞,∞),∀c∈(−∞,∞),c≠a.

We structure this paper as follows.

In Section 2, we derive the new sequential characterizations of the *n*–FTFI capacity for the Cover and Pombra formulation of feedback capacity of the AGN channel (1), i.e., for the Case (I) formulation, $C_{n}^{fb}\left(\kappa \right)$. We also derive analogous sequential characterizations for $C_{n}^{fb}\left(\kappa ,s\right)$ and for the Case (II) formulation, $C_{n}^{fb,S}\left(\kappa ,s\right)$, i.e., when Conditions 1 and 2 hold, to illustrate their fundamental differences.

In Section 3, we present the asymptotic analysis of feedback capacity for the Case (I) formulation. In Section 4, we treat the Case (II) formulation.

This paper contains several examples and makes comparisons to the existing literature.

## 2. Sequential Characterizations of n–FTFI Capacity for Case (I) Formulation

In this section, we derive equivalent sequential characterizations for the following:

(i) $C_{n}^{fb}\left(\kappa \right)$ defined by (2) of the Case (I) formulation, i.e., for the Cover and Pombra *n*–FTFI capacity characterization (34);

(ii) $C_{n}^{fb}\left(\kappa ,s\right)$ defined by (3), as a degenerated case of $C_{n}^{fb}\left(\kappa \right)$;

(iii) $C_{n}^{fb,S}\left(\kappa ,s\right)$ defined by (4) of the Case (II) formulation, as a degenerated case of $C_{n}^{fb}\left(\kappa \right)$.

We organize the presentation of the material as follows.

(1) Section 2.1. Here, we introduce our notation.

(2) Section 2.2. The main result is Theorem 1, which provides an equivalent sequential characterization of the Cover and Pombra characterization of the *n*–FTFI capacity, $C_{n}^{fb}\left(\kappa \right)$, i.e., of (33) and (34). Our derivation proceeds as follows. We apply a linear transformation to the Cover and Pombra Gaussian optimal channel input $X^{n}$ (35), to represent $X_{t}$, with a linear function of $\left(V^{t-1},Y^{t-1}\right)$ or equivalently $\left(X^{t-1},Y^{t-1}\right)$ and an orthogonal Gaussian innovations process $Z_{t}$, which is independent of $\left(Z^{t-1},X^{t-1},V^{t-1},Y^{t-1}\right)$ for t=1,…,n.

Subsequently, we apply Theorem 1 to the time-varying PO-SS$\left(a_{t},c_{t},b_{t}^{1},b_{t}^{2},d_{t}^{1},d_{t}^{2}\right)$ noise (see Example 1) to the non-stationary autoregressive moving average, ARMA (a,c),a∈(−∞,∞),c∈(−∞,∞) noise, and to the stationary ARMA (a,c),a∈(−1,1),c∈(−1,1) noise (see Example 2), which is found in many references, such as [4]. It will become apparent that our characterizations of *n*–FTFI capacity are fundamentally different from past studies.

(3) Section 2.3. The main result is Theorem 3, which provides a simplified characterization of the sequential characterization of the *n*–FTFI capacity, $C_{n}^{fb}\left(\kappa \right)$, given in Theorem 1 (i.e., the equivalent of (34)), for the time-varying AGN channel (1) driven by the PO-SS realization of Definition 2, for the code of Definition 1. The *n*–FTFI capacity of Theorem 3 is expressed in terms of solutions to two DREs. Our derivation is based on identifying a *finite-dimensional sufficient statistic* to express $X_{t}$ as a functional of the sufficient statistic, instead of $\left(V^{t-1},Y^{t-1}\right)$ or $\left(X^{t-1},Y^{t-1}\right)$, and an orthogonal Gaussian innovations process.

(4) Section 2.4. The main results is Corollary 6, which is an application of Theorem 3 (i.e., the sufficient statistic representation), to the ARMA (a,c),a∈(−∞,∞),c∈(−∞,∞) noise of Example 2. This example shows that the *n*–FTFI capacity is expressed in terms of solutions to two DREs.

From Corollary 6, the following will become apparent:

(i) Neither the time-domain characterization [4] (Theorem 6.1) (see [4]) (Theorem 5.3) nor the frequency domain characterization [4] (Theorem 4.1) correspond to the Cover and Pombra characterization of feedback capacity.

(5) Section 2.5. The main results is Corollary 7, which gives the *n*–FTFI capacity for the Case (II) formulation, as a degenerate case of the Case (I) formulation, i.e., of Theorem 3.

(6) Section 2.6. The main result is Proposition 2, which further clarifies the following.

(i) The formulation of [8] and the formulation that led to [4] (Theorem 6.1) are based on the Case (II) formulation, as well as (ii) some of the oversights in [4,7,9,11,12].

### 2.1. Notation

Throughout this paper, we use the following notation:

$Z\overset{▵}{=}\left\{\ldots ,-1,0,1,\ldots \right\},Z_{+}\overset{▵}{=}\left\{1,\ldots \right\},Z_{+}^{n}\overset{▵}{=}\left\{1,2,\ldots ,n\right\}$, where *n* is a finite positive integer.
  $R\overset{▵}{=}\left(-\infty ,\infty \right)$, and $R^{m}$ is the vector space of tuples of the real numbers for an integer $n\in Z_{+}$.
  $C\overset{▵}{=}\left\{a+jb:\left(a,b\right)\in R\times R\right\}$ is the space of complex numbers.
  $R^{n\times m}$ is the set of *n* by *m* matrices with entries from the set of real numbers for integers $\left(n,m\right)\in Z_{+}\times Z_{+}$.
  $D_{o}\overset{▵}{=}\left\{c\in C:|c|<1\right\}$ is the open unit disc of the space of complex number C.
  $S_{+}^{n\times n},n\in Z_{+}$ (resp. $S_{++}^{n\times n}$) denotes the set of positive semidefinite (resp. positive definite) symmetric matrices with elements in the real numbers and of size n×n. Thus, $A\in S_{+}^{n\times n}$ if for all $w\in R^{n}$, $w^{T}Aw\geq 0$. Positive semidefiniteness is denoted by A⪰0 and (strict) positive definiteness by A≻0. $I_{n\times n}\in S_{++}^{n\times n},n\in Z_{+}$ denotes the identity matrix, $tr\left(A\right)$ denotes the trace of any matrix $A\in R^{n\times n},n\in Z_{+}$.
  spec(A)⊂C is the Spectrum of a matrix $A\in R^{q\times q},q\in Z_{+}$ (the set of all its eigenvalues). A matrix $A\in R^{q\times q}$ is called exponentially stable if all its eigenvalues are within the open unit disc, i.e., $spec\left(A\right)\subset D_{o}$.
  $\left(\Omega ,F,P\right)$ denotes a probability space. Given a random variable $X:\Omega \to R^{n_{x}},n_{x}\in Z_{+}^{n}$, its induced distribution on $R^{n_{x}}$ is denoted by $P_{X}$.
  $P_{X}\in N\left(\mu_{X},K_{X}\right),K_{X}⪰0$ denotes a Gaussian distributed RV *X*, with mean value $\mu_{X}$ and covariance matrix $K_{X}=cov\left(X,X\right)⪰0$, defined by
  (47) $$\begin{array}{c} \mu_{X}\overset{▵}{=}E\left\{X\right\},\;K_{X}=cov\left(X,X\right)\overset{▵}{=}E\left\{\left(X-E\left\{X\right\}\right){\left(X-E\left\{X\right\}\right)}^{T}\right\}. \end{array}$$
  Given another Gaussian random variable $Y:\Omega \to R^{n_{y}},n_{y}\in Z_{+}^{n}$, which is jointly Gaussian distributed with *X*, i.e., the joint distribution is $P_{X,Y}$, the conditional covariance of *X* given *Y* is defined by
  (48) $$\begin{array}{cc} K_{X|Y}=cov\left(X,X|Y\right)\overset{▵}{=} & E\left\{\left(X-E\left\{X|Y\right\}\right){\left(X-E\left\{X|Y\right\}\right)}^{T}|Y\right\} \end{array}$$
  (49) $$\begin{array}{cc} = & E\left\{\left(X-E\left\{X|Y\right\}\right){\left(X-E\left\{X|Y\right\}\right)}^{T}\right\} \end{array}$$
  where the last equality is due to a property of jointly Gaussian distributed RVs.
  Given three arbitrary RVs (X,Y,Z) with induced distribution $P_{X,Y,Z}$, the RVs (X,Z) are called conditionally independent given the RV *Y* if $P_{Z|X,Y}=P_{Z|Y}$. This conditional independence is often denoted by X↔Y↔Z, which is a Markov chain.

### 2.2. Preliminary Characterizations of n–FTFI Capacity of AGN Channels Driven by Correlated Noise

We start with preliminary calculations for the feedback code of Definition 1, which we use to prove Theorem 1. These calculations are introduced for the sake of clarity and to establish our notation.

For the feedback code of Definition 1, by the channel definition (1), i.e., (18), the conditional distribution of $Y_{t}$, given $Y^{t-1}=y^{t-1},X^{t}=x^{t}$, is
  $$\begin{array}{cc} P\left\{Y_{t}\in dy|Y^{t-1}=y^{t-1},X^{t}=x^{t}\right\}\overset{\left(a\right)}{=} & P\left\{Y_{t}\in dy|Y^{t-1}=y^{t-1},X^{t}=x^{t},V^{t-1}=v^{t-1}\right\} \end{array}$$
  (50) $$\begin{array}{cc} \overset{\left(b\right)}{=} & P_{V_{t}|V^{t-1}}\left(v_{t}:x_{t}+v_{t}\in dy\right),\;\;t=2,\ldots ,n \end{array}$$
  (51) $$\begin{array}{cc} = & P_{Y_{t}|X_{t},V^{t-1}} \end{array}$$
  (52) $$\begin{array}{cc} \equiv & P_{t}\left(dy|x_{t},v^{t-1}\right), \end{array}$$
  (53) $$\begin{array}{cc} P\left\{Y_{1}\in dy|Y^{0}=y^{0},X^{1}=x^{1}\right\}= & P_{Y_{1}|X_{1}}\equiv P_{1}\left(dy|x_{1}\right). \end{array}$$
  where (a) is due to (1) and (b) is due to (18). We introduce the set of channel input distributions with feedback, which are consistent with the code of Definition 1, not necessarily generated by the messages *W*, as follows:
  (54) $$\begin{array}{c} P_{\left[1,n\right]}\left(\kappa \right)\overset{▵}{=}\left\{P_{t}\left(dx_{t}|x^{t-1},y^{t-1}\right)\overset{▵}{=}P_{X_{t}|X^{t-1},Y^{t-1}},t=1,\ldots ,n:\frac{1}{n}E^{P}\left(\sum_{t=1}^{n}{\left(X_{t}\right)}^{2}\right)\leq \kappa \right\}. \end{array}$$
  By Definition 1, we have $E_{\left[1,n\right]}\left(\kappa \right)\subseteq P_{\left[1,n\right]}\left(\kappa \right)$. Moreover, by the channel definition, any pair of the sequence triple $\left(V^{t},X^{t},Y^{t}\right)$ uniquely defines the remaining sequence. Thus, the identity holds:
  (55) $$\begin{array}{cc} \; & {\bar{P}}_{\left[1,n\right]}\left(\kappa \right)\overset{▵}{=}\left\{{\bar{P}}_{t}\left(dx_{t}|v^{t-1},y^{t-1}\right),t=1,\ldots ,n:\frac{1}{n}E^{\bar{P}}\left(\sum_{t=1}^{n}{\left(X_{t}\right)}^{2}\right)\leq \kappa \right\}=P_{\left[1,n\right]}\left(\kappa \right). \end{array}$$
  We also emphasize that, by Definition 1, for a given feedback encoder strategy $e\left(\cdot \right)\in E_{\left[1,n\right]}\left(\kappa \right)$, i.e., $x_{1}=e_{1}\left(w\right),x_{2}=e_{2}\left(w,x_{1},y_{1}\right),\ldots ,x_{n}=e_{n}\left(w,x^{n-1},y^{n-1}\right)$ the conditional distributions of $Y_{t}$ given $\left(Y^{t-1},W\right)=\left(y^{t-1},w\right)$ is obtained as follows:
  (56) $$\begin{array}{cc} P_{Y_{t}|W,Y^{t-1}}^{e}\left(dy_{t}|,y^{t-1},w\right)\overset{\left(a\right)}{=} & P_{t}\left(dy_{t}|\left\{e_{j}\left(w,x^{j-1},y^{j-1}\right):j=1,\ldots ,t\right\},y^{t-1},w\right) \end{array}$$
  (57) $$\begin{array}{cc} \overset{\left(b\right)}{=} & P_{t}\left(dy_{t}|\left\{e_{j}\left(w,x^{j-1},y^{j-1}\right):j=1,\ldots ,t\right\},y^{t-1},v^{t-1},w\right) \end{array}$$
  (58) $$\begin{array}{cc} \overset{\left(c\right)}{=} & P_{t}\left(dy_{t}|\left\{e_{j}\left(w,x^{j-1},y^{j-1}\right):j=1,\ldots ,t\right\},v^{t-1},w\right) \end{array}$$
  (59) $$\begin{array}{cc} \overset{\left(d\right)}{=} & P_{t}\left(dy_{t}|\left\{e_{j}\left(w,x^{j-1},y^{j-1}\right):j=1,\ldots ,t\right\},v^{t-1}\right) \end{array}$$
  (60) $$\begin{array}{cc} \overset{\left(e\right)}{=} & P_{t}\left(dy_{t}|e_{t}\left(w,x^{t-1},y^{t-1}\right),v^{t-1}\right) \end{array}$$
  (a) is due to knowledge of the distribution of the strategies $e_{j}\left(\cdot \right),j=1,\ldots ,t$, the code definition, and the recursive substitution, $x_{1}=e_{1}\left(w\right),x_{2}=e_{2}\left(w,x_{1},y_{1}\right),\ldots ,e_{t}\left(w,x^{t-1},y^{t-1}\right)$, where $x^{t-1}$ is specified by the knowledge of the strategies, $e_{j}\left(\cdot \right),j=1,\ldots ,t-1$ and the knowledge of $\left(y^{t-2},w\right)$,
  (b) is due to knowing $x_{j}=e_{j}\left(w,x^{j-1},y^{j-1}\right),y_{j},j=1,\ldots ,t-1$ specifies $v_{j}=y_{j}-x_{j},j=1,\ldots ,t-1$,
  (c) is due to the fact that, any pair of the triple $\left(x^{t},y^{t},v^{t}\right)$ specifies the remaining sequence, i.e., knowing $\left(x^{t-1},v^{t-1}\right)$ specifies $y^{t-1}$, and $y^{t-1}$ is thus redundant,
  (d) is due to the conditional independence $P_{V_{t}|V^{t-1},X^{t},W}=P_{V_{t}|V^{t-1},X^{t}}$,
  (e) is due to (18), i.e., $P_{V_{t}|V^{t-1},X^{t}}=P_{V_{t}|V^{t-1}}$, and the channel definition.

By the channel definition $Y_{t}=X_{t}+V_{t},t=1,\ldots ,n$, each $e\left(\cdot \right)\in E_{\left[0,n\right]}\left(\kappa \right)$ is also expressed as

(61) $$\begin{array}{cc} \; & x_{1}=e_{1}\left(w\right)={\bar{e}}_{1}\left(w\right),\;x_{2}=e_{2}\left(w,x_{1},y_{1}\right)={\tilde{e}}_{2}\left(w,x_{1},v_{1},y_{1}\right)\overset{\left(a\right)}{=}{\bar{e}}_{2}\left(w,v_{1},y_{1}\right),\;\ldots ,\\ \; & x_{n}=e_{n}\left(w,x^{n-1},y^{n-1}\right)={\tilde{e}}_{n}\left(w,x^{n-1},v^{n-1},y^{n-1}\right)\overset{\left(a\right)}{=}{\bar{e}}_{n}\left(w,v^{n-1},y^{n-1}\right),\;w\in M^{\left(n\right)}. \end{array}$$

where (a) is due to the channel definition—i.e., the presence of $x^{t-1}$ in ${\tilde{e}}_{t}\left(\cdot ,v^{t-1},\cdot \right)$ can be removed, since it is redundant—and specified by $\left(v^{t-1},y^{t-1}\right)$. Consequently, we have the identity

(62) $$\begin{array}{cc} {\bar{E}}_{\left[1,n\right]}\left(\kappa \right)≜ & \{x_{1}={\bar{e}}_{1}\left(w\right),x_{2}={\bar{e}}_{2}\left(w,v_{1},y_{1}\right)\ldots ,x_{n}={\bar{e}}_{n}\left(w,v^{n-1},y^{n-1}\right):\\ \; & \frac{1}{n}E^{\bar{e}}\left(\sum_{i=1}^{n}{\left(X_{t}\right)}^{2}\right)\leq \kappa \}=E_{\left[1,n\right]}\left(\kappa \right). \end{array}$$

**Notation 1.**  *For the feedback code of Definition 3, with initial state $S_{1}=s$, known to the encoder and the decoder, the above sets $P_{\left[1,n\right]}\left(\kappa \right),{\bar{P}}_{\left[1,n\right]}\left(\kappa \right),E_{\left[1,n\right]},{\bar{E}}_{\left[1,n\right]}$ are replaced by $P_{\left[0,n\right]}^{s}\left(\kappa \right),{\bar{P}}_{\left[1,n\right]}^{s}\left(\kappa \right)$, $E_{\left[1,n\right]}^{s},{\bar{E}}_{\left[1,n\right]}^{s}$ to indicate that the distributions and codes are ${\bar{P}}_{t}\left(dx_{t}|v^{t-1},y^{t-1},s\right),t=1,\ldots ,x_{1}={\bar{e}}_{1}\left(w,s\right),x_{2}={\bar{e}}_{2}\left(w,v_{1},y_{1},s\right)\ldots ,x_{n}={\bar{e}}_{n}\left(w,v^{n-1},y^{n-1},s\right)$, etc., and these depend on s.*

In the next theorem, we present our preliminary equivalent sequential characterization of the Cover and Pombra characterization $C_{n}^{fb}\left(\kappa \right)$, i.e., of (33), under encoder strategies $E_{\left[0,n\right]}\left(\kappa \right)={\bar{E}}_{\left[0,n\right]}\left(\kappa \right)$ and channel input distributions $P_{\left[0,n\right]}\left(\kappa \right)={\bar{P}}_{\left[0,n\right]}\left(\kappa \right)$. Unlike the Cover and Pombra [5] realization of $X^{n}$, given by (35), at each time *t*, $X_{t}$ is driven by an orthogonal Gaussian process $Z_{t}$.

**Theorem 1.**  *Information structures of maximizing distributions for AGN Channels*

*Consider the AGN channel (1), i.e., with noise distribution $P_{V^{n}}$, and the code of Definition 1. Then, the following hold:*
*(a) The following inequality holds:*
(63) $$\begin{array}{c} \underset{{\bar{E}}_{\left[1,n\right]}\left(\kappa \right)}{\sup}\sum_{t=1}^{n}H^{\bar{e}}\left(Y_{t}|Y^{t-1}\right)\leq \underset{{\bar{P}}_{\left[1,n\right]}\left(\kappa \right)}{\sup}\sum_{t=1}^{n}H^{\bar{P}}\left(Y_{t}|Y^{t-1}\right) \end{array}$$
  *where the conditional (differential) entropy $H^{\bar{e}}\left(Y_{t}|Y^{t-1}\right)$ is evaluated with respect to the probability distribution $P_{t}^{\bar{e}}\left(dy_{t}|y^{t-1}\right)$, defined by*
(64) $$\begin{array}{c} P_{t}^{\bar{e}}\left(dy_{t}|y^{t-1}\right)=\int P_{t}\left(dy_{t}|{\bar{e}}_{t}\left(w,v^{t-1},y^{t-1}\right),v^{t-1}\right)\;P_{t}^{\bar{e}}\left(dw|y^{t-1}\right),\;t=0,\ldots ,n. \end{array}$$
  *and $H^{\bar{P}}\left(Y_{t}|Y^{t-1}\right)$ is evaluated with respect to the probability distribution $P_{t}^{\bar{P}}\left(dy_{t}|y^{t-1}\right)$, defined by*
(65) $$\begin{array}{c} P_{t}^{\bar{P}}\left(dy_{t}|y^{t-1}\right)=\int P_{t}\left(dy_{t}|x_{t},v^{t-1}\right)\;P_{t}^{\bar{P}}\left(dx_{t}|v^{t-1},y^{t-1}\right)\;P_{t}^{\bar{P}}\left(dv^{t-1}|y^{t-1}\right),\;t=0,\ldots ,n. \end{array}$$
*(b) The optimal channel input distribution $\left\{\bar{P}\left(dx_{t}|v^{t-1},y^{t-1}\right),t=1,\ldots ,n\right\}\in {\bar{P}}_{\left[1,n\right]}\left(\kappa \right)$, which maximizes $\sum_{t=1}^{n}H^{\bar{P}}\left(Y_{t}|Y^{t-1}\right)$ of part (a), i.e., the right-hand side of (63), is induced by an input process $X^{n}$, which is conditionally Gaussian, with linear conditional mean, nonrandom conditional covariance and given by*
(66) $$\begin{array}{cc} \; & E^{\bar{P}}\left\{X_{t}|V^{t-1},Y^{t-1}\right\}=\left\{\begin{array}{ccc} \Gamma_{t}^{1}V^{t-1}+\Gamma_{t}^{2}Y^{t-1}, & for & t=2,\ldots ,n\\ 0, & for & t=1, \end{array}\right. \end{array}$$
(67) $$\begin{array}{cc} \; & K_{X_{t}|V^{t-1},Y^{t-1}}\overset{▵}{=}cov\left(X_{t},X_{t}|V^{t-1},Y^{t-1}\right)=K_{Z_{t}}⪰0,\;t=1,\ldots ,n \end{array}$$
  *and such that the average constraint holds and (18) is respected.*
*(c) The optimal channel input distribution $\left\{\bar{P}\left(dx_{t}|v^{t-1},y^{t-1}\right),t=1,\ldots ,n\right\}\in {\bar{P}}_{\left[1,n\right]}\left(\kappa \right)$ of part (b) is induced by a jointly Gaussian process $X^{n}$, with a realization given by*
(68) $$\begin{array}{cc} \; & X_{t}=\sum_{j=1}^{t-1}\Gamma_{t,j}^{1}V_{j}+\sum_{j=1}^{t-1}\Gamma_{t,j}^{2}Y_{j}+Z_{t},\;X_{1}=Z_{1},\;t=2,\ldots ,n, \end{array}$$
(69) $$\begin{array}{cc} \; & \;=\Gamma_{t}^{1}V^{t-1}+\Gamma_{t}^{2}Y^{t-1}+Z_{t}, \end{array}$$
(70) $$\begin{array}{cc} \; & Z_{t}\in N\left(0,K_{Z_{t}}\right),\;t=1,\ldots ,n\;a\;Gaussian\;sequence, \end{array}$$
(71) $$\begin{array}{cc} \; & Z_{t}\;independent\;of\;\left(V^{t-1},X^{t-1},Y^{t-1}\right),\;t=1,\ldots ,n, \end{array}$$
(72) $$\begin{array}{cc} \; & Z^{n}\;independent\;of\;V^{n}, \end{array}$$
(73) $$\begin{array}{cc} \; & \frac{1}{n}E^{\bar{P}}\left\{\sum_{t=1}^{n}{\left(X_{t}\right)}^{2}\right\}\leq \kappa , \end{array}$$
(74) $$\begin{array}{cc} \; & \left(\Gamma_{t}^{1},\Gamma_{t}^{2},K_{Z_{t}}\right)\in \left(-\infty ,\infty \right)\times \left(-\infty ,\infty \right)\times \left[0,\infty \right)\;nonrandom. \end{array}$$
*(d) An equivalent characterization of the n–FTFI capacity $C_{n}^{fb}\left(\kappa \right)$, defined by (33) and (34), is given by*
(75) $$\begin{array}{cc} C_{n}^{fb}\left(\kappa \right)= & \underset{\frac{1}{n}E^{\bar{P}}\left\{\sum_{t=1}^{n}{\left(X_{t}\right)}^{2}\right\}\leq \kappa }{\sup}\sum_{t=1}^{n}H^{\bar{P}}\left(Y_{t}|Y^{t-1}\right)-H\left(V^{n}\right) \end{array}$$
(76) $$\begin{array}{cc} = & \underset{\frac{1}{n}E^{\bar{P}}\left\{\sum_{t=1}^{n}{\left(X_{t}\right)}^{2}\right\}\leq \kappa }{\sup}\sum_{t=1}^{n}\left\{H^{\bar{P}}\left(I_{t}\right)-H\left({\hat{I}}_{t}\right)\right\} \end{array}$$
  *where $I_{t}\;and\;{\hat{I}}_{t}$ are innovations processes defined by*
(77) $$\begin{array}{c} I_{t}\overset{▵}{=}Y_{t}-E^{\bar{P}}\left\{Y_{t}|Y^{t-1}\right\},\;{\hat{I}}_{t}\overset{▵}{=}V_{t}-E\left\{V_{t}|V^{t-1}\right\} \end{array}$$
  *where parts (b) and (c) hold, and where the supremum is over all $(\Gamma_{t}^{1},\Gamma_{t}^{2},K_{Z_{t}}),t=1,\ldots ,n$ of the realization of part (c), which induces the distribution ${\bar{P}}_{t}\left(dx_{t}|v^{t-1},y^{t-1}\right),t=1,\ldots ,n$.*

**Proof.**  See Appendix A.1.  □

**Remark 2.**  *For the code of Definition 3 that assumes knowledge of the initial state $S_{1}=s$, it is easy to verify that $C_{n}^{fb}\left(\kappa ,s\right)$ is directly obtained from Theorem 1, as a degenerate case (an independent derivation is easily produced following the derivation of Corollary 11, with slight variations).*

By utilizing Theorem 1, we can derive the converse coding theorems stated below for the feedback codes of Definitions 1 and 3.

**Theorem 2.**  *Converse coding theorems for codes of Definitions 1 and 3*

*Consider the AGN channel (1).*

*(a) Any achievable rate R for the code of Definition 1 satisfies*
(78) $$\begin{array}{cc} R\leq & C^{fb}\left(\kappa \right)\overset{▵}{=}\lim_{n⟶\infty }\frac{1}{n}C_{n}^{fb}\left(\kappa \right), \end{array}$$
(79) $$\begin{array}{cc} C_{n}^{fb}\left(\kappa \right)= & \underset{{\bar{P}}_{t}\left(dx_{t}|v^{t-1},y^{t-1}\right),t=1,\ldots ,n:\;\frac{1}{n}E^{\bar{P}}\left\{\sum_{t=1}^{n}{\left(X_{t}\right)}^{2}\right\}\leq \kappa }{\sup}\sum_{t=1}^{n}H^{\bar{P}}\left(Y_{t}|Y^{t-1}\right)-H\left(V^{n}\right) \end{array}$$
  *provided the supremum exists and the limit exists, where the right-hand side of (79) is given in Theorem 1(d).*
*(b) Any achievable rate R for the code of Definition 3 (with initial state $S_{1}=s$) satisfies*
(80) $$\begin{array}{cc} R\leq & C^{fb}\left(\kappa ,s\right)\overset{▵}{=}\lim_{n⟶\infty }\frac{1}{n}C_{n}^{fb}\left(\kappa ,s\right), \end{array}$$
(81) $$\begin{array}{cc} C_{n}^{fb}\left(\kappa ,s\right)= & \underset{{\bar{P}}_{t}\left(dx_{t}|v^{t-1},y^{t-1},s\right),t=1,\ldots ,n:\;\frac{1}{n}E_{s}^{\bar{P}}\left\{\sum_{t=1}^{n}{\left(X_{t}\right)}^{2}|S_{1}\right\}\leq \kappa }{\sup}\sum_{t=1}^{n}H^{\bar{P}}\left(Y_{t}|Y^{t-1},s\right)-H\left(V^{n}|s\right). \end{array}$$
  *where $E_{s}^{\bar{P}}\left\{\cdot \right\}$ means the expectation is for a fixed $S_{1}=s$, provided the supremum exists and the limit exists, and where the right-hand side of (81) is obtained from Theorem 1, part (d), by replacing all conditional distributions, entropies, etc., for fixed initial state $S_{1}=s$ (see Notation 1).*

**Proof.**  Following on from standard arguments, we use Fano’s inequality (see also [5]) and Theorem 1.  □

In the next remark, we clarify the equivalence of Theorem 1(d) to Cover and Pombra [5].

**Remark 3.**  *Relation of Theorem 1 and Cover and Pombra [5]*

*(a) From the realization of $X^{n}$ given by (68), we can recover the Cover and Pombra [5] realization (35) by recursive substitution of $Y^{t-1}$ into the right-hand side of (68), as follows:*
(82) $$\begin{array}{cc} X_{t}= & \sum_{j=1}^{t-1}\Gamma_{t,j}^{1}V_{j}+\sum_{j=1}^{t-1}\Gamma_{t,j}^{2}Y_{j}+Z_{t} \end{array}$$
(83) $$\begin{array}{cc} = & \sum_{j=1}^{t-1}\Gamma_{t,j}^{1}V_{j}+\sum_{j=1}^{t-2}\Gamma_{t,j}^{2}Y_{j}+\Gamma_{t,t-1}^{2}\left(X_{t-1}+Z_{t-1}\right)+Z_{t} \end{array}$$
(84) $$\begin{array}{cc} = & \sum_{j=1}^{t-1}B_{t,j}V_{j}+{\bar{Z}}_{t},\;by\;recursive\;substitution\;of\;X_{1},\ldots ,X_{t-1},Y_{1},\ldots ,Y_{t-2} \end{array}$$
  *for some ${\bar{Z}}_{t}\in \left(0,K_{{\bar{Z}}_{t}}\right)$ which is jointly correlated, and some nonrandom $B_{t,j}$, as given by (35) and (36).*
*(b) Unlike the Cover and Pombra [5] realization of $X^{n}$, i.e., (35), the realization of $X^{n}$ given by (68) or in vector form by (69) is such that, at each time t, $X_{t}$ depends on $\left(V^{t-1},Y^{t-1},Z_{t}\right)$ or in vector form on $\left(V^{t-1},Y^{t-1},Z_{t}\right)$, where $Z^{t}$ is an innovations or orthogonal process, i.e., (71) holds.*
*(c) In subsequent parts of the paper, we derive an equivalent sequential characterization of the Cover and Pombra n–FTFI capacity (34), which is simplified further with the use of a sufficient statistic (that satisfies a Markov recursion).*

To characterize $C_{n}^{fb}\left(\kappa \right)$ using Theorem 1, part (d), we need to compute the (differential) entropy $H(V^{n})$ of $V^{n}$. The following lemma is useful in this respect:

**Lemma 1.**  *Entropy $H(V^{n})$ calculation from generalized Kalman filter of the PO-SS noise realization.*

*Consider the PO-SS realization of $V^{n}$ of Definition 2. Define the conditional covariance and conditional mean of $S_{t}$ given $V^{t-1}$ by*

(85) $$\begin{array}{cc} \Sigma_{t}\overset{▵}{=} & cov(S_{t},S_{t}|V^{t-1})=E\left\{\left(S_{t}-{\hat{S}}_{t}\right){\left(S_{t}-{\hat{S}}_{t}\right)}^{T}|V^{t-1}\right\},\;{\hat{S}}_{t}\overset{▵}{=}E\left\{S_{t}|V^{t-1}\right\},\;t=2,\ldots ,n, \end{array}$$

(86) $$\begin{array}{cc} \Sigma_{1}\overset{▵}{=} & cov(S_{1},S_{1})=K_{S_{1}},\;{\hat{S}}_{1}\overset{▵}{=}\mu_{S_{1}}. \end{array}$$

*Then, the following hold:*

*(a) The conditional distribution of $V_{t}$ conditioned on $V^{t-1}$ is Gaussian, i.e.,*
(87) $$\begin{array}{c} P_{V_{t}|V^{t-1}}\in N\left(\mu_{V_{t}|V^{t-1}},K_{V_{t}|V^{t-1}}\right),\;t=1,\ldots ,n\; \end{array}$$
  *where $\mu_{V_{t}|V^{t-1}}\overset{▵}{=}E\left\{V_{t}|V^{t-1}\right\},K_{V_{t}|V^{t-1}}\overset{▵}{=}cov(V_{t},V_{t}|V^{t-1})$.*
*(b) The conditional mean and covariance $\mu_{V_{t}|V^{t-1}},K_{V_{t}|V^{t-1}}$ are given by the generalized Kalman filter recursions, as follows:*
*(i) The optimal mean square error estimate ${\hat{S}}_{t}$ satisfies the generalized Kalman filter recursion*
(88) $$\begin{array}{cc} \; & {\hat{S}}_{t+1}=A_{t}{\hat{S}}_{t}+M_{t}\left(\Sigma_{t}\right){\hat{I}}_{t},\;{\hat{S}}_{1}=\mu_{S_{1}}, \end{array}$$
(89) $$\begin{array}{cc} \; & M_{t}\left(\Sigma_{t}\right)\overset{▵}{=}\left(A_{t}\Sigma_{t}C_{t}^{T}+B_{t}K_{W_{t}}N_{t}^{T}\right){\left(N_{t}K_{W_{t}}N_{t}^{T}+C_{t}\Sigma_{t}C_{t}^{T}\right)}^{-1}, \end{array}$$
(90) $$\begin{array}{cc} \; & {\hat{I}}_{t}\overset{▵}{=}V_{t}-E\left\{V_{t}|V^{t-1}\right\}=V_{t}-C_{t}{\hat{S}}_{t}=C_{t}\left(S_{t}-{\hat{S}}_{t}\right)+N_{t}W_{t},\;t=1,\ldots ,n, \end{array}$$
(91) $$\begin{array}{cc} \; & {\hat{I}}_{t}\in N\left(0,K_{{\hat{I}}_{t}}\right),\;t=1,\ldots ,n\;is\;an\;orthogonal\;innovations\;process,\;i.e.,\;{\hat{I}}_{t}\;is\;independent\;of\\ \; & {\hat{I}}_{s},\;for\;all\;t\neq s,\;and\;{\hat{I}}_{t}\;is\;independent\;ofV^{t-1}, \end{array}$$
(92) $$\begin{array}{cc} \; & K_{{\hat{I}}_{t}}\overset{▵}{=}cov\left({\hat{I}}_{t},{\hat{I}}_{t}\right)=C_{t}\Sigma_{t}C_{t}^{T}+N_{t}K_{W_{t}}N_{t}^{T}. \end{array}$$
  *(ii) The error $E_{t}\overset{▵}{=}S_{t}-{\hat{S}}_{t}$ satisfies the recursion*
(93) $$\begin{array}{cc} E_{t+1}= & M_{t}^{CL}\left(\Sigma_{t}\right)E_{t}+\left(B_{t}-M\left(\Sigma_{t}\right)N_{t}\right)W_{t},\;E_{1}=S_{1}-{\hat{S}}_{1},\;t=1,\ldots ,n, \end{array}$$
(94) $$\begin{array}{cc} M_{t}^{CL}\left(\Sigma_{t}\right)\overset{▵}{=} & A_{t}-M_{t}\left(\Sigma_{t}\right)C_{t}. \end{array}$$
  *(iii) The covariance of the error is such that $E\left\{E_{t}E_{t}^{T}\right\}=\Sigma_{t}$ and satisfies the generalized matrix DRE*
(95) $$\begin{array}{cc} \Sigma_{t+1}= & A_{t}\Sigma_{t}A_{t}^{T}+B_{t}K_{W_{t}}B_{t}^{T}-\left(A_{t}\Sigma_{t}C_{t}^{T}+B_{t}K_{W_{t}}N_{t}^{T}\right){\left(N_{t}K_{W_{t}}N_{t}^{T}+C_{t}\Sigma_{t}C_{t}^{T}\right)}^{-1}\\ \; & \;.{\left(A_{t}\Sigma_{t}C_{t}^{T}+B_{t}K_{W_{t}}N_{t}^{T}\right)}^{T},\;t=1,\ldots ,n,\;\Sigma_{1}=K_{S_{1}}⪰0,\;\Sigma_{t}⪰0. \end{array}$$
  *(iv) The conditional mean and covariance $\mu_{V_{t}|V^{t-1}},K_{V_{t}|V^{t-1}}$ are given by*
(96) $$\begin{array}{cc} \mu_{V_{t}|V^{t-1}}= & C_{t}{\hat{S}}_{t},\;t=1,\ldots ,n, \end{array}$$
(97) $$\begin{array}{cc} K_{V_{t}|V^{t-1}}= & K_{{\hat{I}}_{t}}=C_{t}\Sigma_{t}C_{t}^{T}+N_{t}K_{W_{t}}N_{t}^{T},\;t=1,\ldots ,n. \end{array}$$
  *(v) The entropy of $V^{n}$ is given by*
(98) $$\begin{array}{c} H\left(V^{n}\right)=\sum_{t=1}^{n}H\left({\hat{I}}_{t}\right)=\frac{1}{2}\sum_{t=1}^{n}\log\left(2\pi e\left[C_{t}\Sigma_{t}C_{t}^{T}+N_{t}K_{W_{t}}N_{t}^{T}\right]\right) \end{array}$$

**Proof.**  (a,b).(i–iv). The generalized Kalman filter of the PO-SS realization of $V^{n}$ and accompanied statements can be found in many textbooks, i.e., [16]. However, it is noted that ${\hat{I}}_{t},t=2,\ldots ,n$, ${\hat{I}}_{1}=V_{1}$ are all independent Gaussian. For example, to show (93), we write the recursion for $E_{t}=S_{t}-{\hat{S}}_{t}$ using part (i) and the realization of $S_{t}$, part (b). (v) By the chain rule of joint entropy, we have

(99) $$\begin{array}{cc} H(V^{n})= & H\left(V_{1}\right)+\sum_{t=2}^{n}H\left(V_{t}|V^{t-1}\right) \end{array}$$

(100) $$\begin{array}{cc} = & H\left(V_{1}\right)+\sum_{t=2}^{n}H\left(V_{t}-E\left\{V_{t}|V^{t-1}\right\}|V^{t-1}\right) \end{array}$$

(101) $$\begin{array}{cc} = & H\left(V_{1}\right)+\sum_{t=2}^{n}H\left({\hat{I}}_{t}\right),\;\text{by orthogonality of}\;{\hat{I}}_{t}\overset{▵}{=}V_{t}-E\left\{V_{t}|V^{t-1}\right\}\mathrm{and}V^{t-1} \end{array}$$

From (101) and (97), we have (98) from the entropy formula of Gaussian RVs.  □

The next corollary of the entropy $H(V^{n}|s)$ follows directly from Lemma 1 when $S_{1}=s$ is fixed.

**Corollary 1.**  *Conditional entropy $H\left(V^{n}|s\right),S_{1}=s$ of the PO-SS noise realization.*

*Consider the PO-SS realization of $V^{n}$ of Definition 2, for fixed $S_{1}=s$, and denote the state process generated by recursion (19) via (we often use the notation $S_{t}=S_{t}^{s}$ to emphasize that the $S_{t}$ process is generated for $S_{1}=S_{1}^{s}=s$ fixed) $S_{t}=S_{t}^{s},t=2,\ldots ,n,S_{1}=S_{1}^{s}=s$. Replace the conditional covariance and conditional mean (85) and (86) by*

(102) $$\begin{array}{cc} \Sigma_{t}^{s}\overset{▵}{=} & cov(S_{t}^{s},S_{t}^{s}|V^{t-1},S_{1}^{s})=E\left\{\left(S_{t}^{s}-{\hat{S}}_{t}^{s}\right){\left(S_{t}^{s}-{\hat{S}}_{t}^{s}\right)}^{T}|V^{t-1},S_{1}^{s}\right\}, \end{array}$$

(103) $$\begin{array}{cc} {\hat{S}}_{t}^{s}\overset{▵}{=} & E\left\{S_{t}^{s}|V^{t-1},S_{1}^{s}\right\},\;t=2,\ldots ,n,\;\;S_{1}^{s}=s,\;{\hat{S}}_{1}^{s}\overset{▵}{=}s,\;\Sigma_{1}^{s}\overset{▵}{=}cov(S_{1}^{s},S_{1}^{s}\left|S_{1}^{s}\right)=0. \end{array}$$

*Then, all statements of Lemma 1 hold, with the following changes:*

(104) $$\begin{array}{c} \Sigma_{t}⟼\Sigma_{t}^{s},\;\Sigma_{1}^{s}=0,\;P_{V_{t}|V^{t-1}}⟼P_{V_{t}|V^{t-1},S_{1}^{s}},\;{\hat{S}}_{t}⟼{\hat{S}}_{t}^{s},\;{\hat{S}}_{1}^{s}=s,\;etc,\;t=1,\ldots ,n. \end{array}$$

*In particular, the conditional entropy of the $V^{n}$ conditioned on $S_{1}=S_{1}^{s}=s$ is given by*

(105) $$\begin{array}{c} H\left(V^{n}|s\right)=\frac{1}{2}\sum_{t=1}^{n}\log\left(2\pi e\left[C_{t}\Sigma_{t}^{s}C_{t}^{T}+N_{t}K_{W_{t}}N_{t}^{T}\right]\right) \end{array}$$

*where $\Sigma_{t}^{s},t=2,\ldots ,n$ satisfies the generalized DRE (95) with initial condition $\Sigma_{1}^{s}=0$.*

**Proof.**  We continue directly from Lemma 1 and (102), (103).  □

Next, we introduce an example of a PO-SS realization of the noise that we often use in this paper.

**Example 1.**  *A time-varying PO-SS$\left(a_{t},c_{t},b_{t}^{1},b_{t}^{2},d_{t}^{1},d_{t}^{2}\right)$ noise realization is defined by*

(106) $$\begin{array}{cc} \; & S_{t+1}=a_{t}S_{t}+b_{t}^{1}W_{t}^{1}+b_{t}^{2}W_{t}^{2},\;t=1,2,\ldots ,n-1 \end{array}$$

(107) $$\begin{array}{cc} \; & V_{t}=c_{t}S_{t}+d_{t}^{1}W_{t}^{1}+d_{t}^{2}W_{t}^{2},\;t=1,\ldots ,n, \end{array}$$

(108) $$\begin{array}{cc} \; & S_{1}\in N\left(\mu_{S_{1}},K_{S_{1}}\right),\;K_{S_{1}}\geq 0,\;W_{t}^{i}\in N\left(0,K_{W_{t}^{i}}\right),\;K_{W_{t}^{i}}\geq 0,\;i=1,2,\;t=1,\ldots ,n, \end{array}$$

(109) $$\begin{array}{cc} \; & W^{1,n}\;and\;W^{2,n}indep.\;seq.\;and\;indep.\;of\;S_{1}, \end{array}$$

(110) $$\begin{array}{cc} \; & a_{t}\in R,\;c_{t}\in R,\;b_{t}^{i}\in R,\;d_{t}^{i}\in R,\;i=1,2,\forall t\;are\;nonrandom, \end{array}$$

(111) $$\begin{array}{cc} \; & b_{t}\circ b_{t}\overset{▵}{=}{\left(b_{t}^{1}\right)}^{2}K_{W_{t}^{1}}+{\left(b_{t}^{2}\right)}^{2}K_{W_{t}^{2}},\;b_{t}\circ d_{t}\overset{▵}{=}b_{t}^{1}K_{W_{t}^{1}}d_{t}^{1}+b_{t}^{2}K_{W_{t}^{2}}d_{t}^{2}, \end{array}$$

(112) $$\begin{array}{cc} \; & d_{t}\circ d_{t}\overset{▵}{=}{\left(d_{t}^{1}\right)}^{2}K_{W_{t}^{1}}+{\left(d_{t}^{2}\right)}^{2}K_{W_{t}^{2}}>0,\;\forall t. \end{array}$$

The next corollary is an application of Lemma 1 to the time-varying PO-SS noise of Example 1.

**Corollary 2.**  *The entropy $H(V^{n})$ of the PO-SS$\left(a_{t},c_{t},b_{t}^{1},b_{t}^{2},d_{t}^{1},d_{t}^{2}\right)$ noise of Example 1 is computed from Lemma 1 with the following changes:*

(113) $$\begin{array}{cc} \; & C_{t}⟼c_{t},\;A_{t}⟼a_{t},\;B_{t}K_{W_{t}}N_{t}^{T}⟼b_{t}\circ d_{t},\\ \; & B_{t}K_{W_{t}}B_{t}^{T}⟼b_{t}\circ b_{t},\;N_{t}K_{W_{t}}N_{t}^{T}⟼d_{t}\circ d_{t},\;t=1,\ldots ,n. \end{array}$$

**Proof.**  This is easily verified.  □

From Corollary 2, we have the following observations:

**Remark 4.**  *Consider the PO-SS$\left(a_{t},c_{t},b_{t}^{1},b_{t}^{2},d_{t}^{1},d_{t}^{2}\right)$ noise of Example 1. Then, the following hold.*

*(a) Consider the code of Definition 2. At each time t, the optimal channel input process $X^{n}$ is either realized by (35), or equivalently by (68), i.e.,*
(114) $$\begin{array}{c} X_{t}=\sum_{j=1}^{t-1}B_{t,j}V_{t,j}+{\bar{Z}}_{t}=\sum_{j=1}^{t-1}\Gamma_{t,j}^{1}V_{j}+\sum_{j=1}^{t-1}\Gamma_{t,j}^{2}Y_{j}+Z_{t},\;t=1,2,\ldots ,n. \end{array}$$
  *Moreover, $X_{t}$ cannot be expressed in terms of the state $S^{t}$ because, by (106) and (107), the noise sequence $V^{t-1}$ does not specify $S^{t}$ for t=1,…,n.*
*(b) Consider the code of Definition 3, i.e., with a fixed initial state $S_{1}=S_{1}^{s}=s$. With Corollary 1 using (113) $H(V^{n}|s)$ is computed from Lemma 1, with $\Sigma_{1}=\Sigma_{1}^{s}=0$, and (105) reduces to*
(115) $$\begin{array}{c} H\left(V^{n}|s\right)=\frac{1}{2}\sum_{t=1}^{n}\log\left(2\pi e\left[{\left(c_{t}\right)}^{2}\Sigma_{t}^{s}+d_{t}\circ d_{t}\right]\right) \end{array}$$
  *where $\Sigma_{t}^{s}$ is the solution of (95) with $\Sigma_{1}=\Sigma_{1}^{s}=0$ (using (113)).*

We also apply our results to various versions of the autoregressive moving average (ARMA) noise model, such as the double-side and single-sided, stationary version of the ARMA noise, previously analyzed in [4] and in many other papers, to illustrate fundamental differences of Case (I) and Case (II) formulations.

**Example 2.**  *The time-invariant ARMA(a,c) noise*

*(a) The time-invariant one-sided, stable or unstable, autoregressive moving average (ARMA(a,c),a∈(−∞,∞),c∈(−∞,∞)) noise is defined by*
(116) $$\begin{array}{cc} \; & V_{t}=cV_{t-1}+W_{t}-aW_{t-1},\;\forall t\in Z_{+}\overset{▵}{=}\left\{1,2,\ldots \right\}, \end{array}$$
(117) $$\begin{array}{cc} \; & V_{0}\in N\left(0,K_{V_{0}}\right),\;K_{V_{0}}\geq 0,\;W_{0}\in N\left(0,K_{W_{0}}\right),\;K_{W_{0}}\geq 0, \end{array}$$
(118) $$\begin{array}{cc} \; & W_{t}\in N\left(0,K_{W}\right),\;K_{W}>0,\;\forall t\in Z_{+},\;\left\{W_{0},W_{1},\ldots ,W_{n}\right\}\;indep.\;seq.\;and\;indep.\;of\;V_{0}, \end{array}$$
(119) $$\begin{array}{cc} \; & c\in (-\infty ,\infty ),\;a\in (-\infty ,\infty ),\;c\neq a. \end{array}$$
  *To express the AR(a,c) in state space form, we define the state variable of the noise by*
(120) $$\begin{array}{c} S_{t}\overset{▵}{=}\frac{cV_{t-1}-aW_{t-1}}{c-a},\;\forall t\in Z_{+} \end{array}$$
  *Then, the state space realization of $V^{n}$ is*
(121) $$\begin{array}{cc} \; & S_{t+1}=cS_{t}+W_{t},\;\forall t\in Z_{+}, \end{array}$$
(122) $$\begin{array}{cc} \; & V_{t}=\left(c-a\right)S_{t}+W_{t},\;\forall t\in Z_{+}, \end{array}$$
(123) $$\begin{array}{cc} \; & K_{S_{1}}=\frac{{\left(c\right)}^{2}K_{V_{0}}+{\left(a\right)}^{2}K_{W_{0}}}{{\left(c-a\right)}^{2}},\;K_{V_{0}}\geq 0,\;K_{W_{0}}\geq 0\;both\;given. \end{array}$$
  *We note that the AR(a,c) is not necessarily stationary or asymptotically stationary.*
*A special case of the AR(a,c) is the AR(c) noise (i.e., with a=0) defined by*
(124) $$\begin{array}{cc} \; & V_{t}=cV_{t-1}+W_{t},\;t=1,2,\ldots ,\;K_{V_{0}}\geq 0,\;K_{W}>0. \end{array}$$
  *(b) Double-sided wide-sense stationary ARMA(a,c),a∈[−1,1],c∈(−1,1) noise. A double-sided wide-sense stationary ARMA(a,c) noise is defined by*
(125) $$\begin{array}{c} V_{t}=cV_{t-1}+W_{t}-aW_{t-1},\;\forall t\in Z\overset{▵}{=}\left\{\ldots ,-1,0,1,\ldots \right\},\;|a|\leq 1,\;|c|<1. \end{array}$$
  *where $W_{t},\forall t\in Z$ is an independent and identically distributed Gaussian sequence, i.e., $W_{t}\in N\left(0,K_{W}\right)$, ∀t. The power spectral density (PSD) of the wide-sense stationary noise (this corresponds to [4] (Equation (43) with L=1)) is given by*
(126) $$\begin{array}{cc} S_{V}\left(e^{j\theta }\right)\overset{▵}{=} & K_{W}\frac{\left(1-ae^{i\theta }\right)\left(1-ae^{-i\theta }\right)}{\left(1-ce^{i\theta }\right)\left(1-ce^{-i\theta }\right)},\;|c|<1,\;|a|\leq 1,\;c\neq a,\;K_{W}>0. \end{array}$$
  *We define the state process by*
(127) $$\begin{array}{c} S_{t}\overset{▵}{=}\frac{cV_{t-1}-aW_{t-1}}{c-a},\;\forall t\in Z. \end{array}$$
  *Then, the stationary state space realization of $V_{t},\forall t\in Z$ is*
(128) $$\begin{array}{cc} \; & S_{t+1}=cS_{t}+W_{t},\;\forall t\in Z, \end{array}$$
(129) $$\begin{array}{cc} \; & V_{t}=\left(c-a\right)S_{t}+W_{t},\;\forall t\in Z \end{array}$$
  *provided that the initial covariances $cov\left(S_{t},S_{t}\right),cov\left(S_{t},V_{t}\right),cov\left(V_{t},V_{t}\right)$ are chosen appropriately to ensure stationarity (see Proposition 1).*
*(c) One-sided wide-sense stationary ARMA(a,c),a∈[−1,1],c∈(−1,1). The one-sided wide-sense stationary ARMA(a,c) noise is defined as in part (b) with $\forall t\in Z\overset{▵}{=}\left\{\ldots ,-1,0,1,\ldots ,\right\}$ replaced by $\forall t\in Z_{+}\overset{▵}{=}\left\{1,2,\ldots ,\right\}$ and (127)–(129), which hold, $\forall t\in Z_{+}$, provided that the initial covariances are chosen appropriately (see Proposition 1).*

For the AR(a,c) noise, we clarify differences of the feedback codes of Definitions 1 and 3 in the next remark, as well as of the Case (I) formulation versus the Case (II) formulation (and we discuss implication to results in [4,7,9,11,12]).

**Remark 5.**  *ARMA(a,c) noise of Example 2*

*(a) Consider any of the AR(a,c) of Example 2. For the code of Definition 2, the channel input process $X^{n}$ cannot be expressed in terms of the state $S^{n}$ (also see Remark 4(a)).*
*(b) Consider the non-stationary AR(a,c),a∈(−∞,∞),c∈(−∞,∞) of Example 2(a).*
*(i) Assume the code of Definition 3, with initial state $V_{0}=v_{0}$ known to the encoder. By (120),*
(130) $$\begin{array}{c} S_{1}=S_{1}^{v_{0}}=\frac{cv_{0}-aW_{0}}{c-a},\;V_{0}=v_{0}; \end{array}$$
  *hence, knowledge of $V_{0}=v_{0}$ at the encoder does not determine $S_{1}^{v_{0}}$ because the encoder requires knowledge of $W_{0}$ for this to hold. It then follows that $H(V^{n}|v_{0})$ is computed from Corollary 1 as follows:*
(131) $$\begin{array}{c} \Sigma_{1}=\Sigma_{1}^{v_{0}}=\frac{{\left(a\right)}^{2}K_{W_{0}}}{{\left(c-a\right)}^{2}},\;\text{(105)}\;reduces\;to\;H\left(V^{n}|v_{0}\right)=\frac{1}{2}\sum_{t=1}^{n}\log\left(2\pi e\left[{\left(c\right)}^{2}\Sigma_{t}^{v_{0}}+K_{W}\right]\right) \end{array}$$
  *where $\Sigma_{t}^{v_{0}}$ is the solution of (95) with initial data $\Sigma_{1}=K_{S_{1}}=\Sigma_{1}^{v_{0}},K_{W_{0}}\geq 0$.*
*(ii) Assume the code of Definition 3, with initial state $S_{1}=s$ or $\left(V_{0},W_{0}\right)=\left(v_{0},w_{0}\right)$, known to the encoder. Then, by Corollary 1,*
(132) $$\begin{array}{c} H\left(V^{n}|v_{0},w_{0}\right)=\frac{1}{2}\sum_{t=1}^{n}\log\left(2\pi eK_{W}\right). \end{array}$$
  *By (120), $S_{1}\overset{▵}{=}\frac{cV_{0}-aW_{0}}{c-a}$, and a necessary condition for Conditions 1 of Section 1.1 to hold is the following: both $\left(V_{0},W_{0}\right)=\left(v_{0},w_{0}\right)$ are known to the encoder and the decoder.*
*(c) The statements of parts (a) and (b) also hold for the double-sided and the one-sided wide-sense stationary AR(a,c),a∈[−1,1],c∈(−1,1) of Example 2(b,c).*
*(d) The Case (II) formulation discussed in Section 1.1 requires Conditions 1 and 2 to hold. For any of the AR(a.c) noise models, Conditions 1 and 2 hold if and only if $S_{1}=s_{1}$ or $\left(V_{0},W_{0}\right)=\left(v_{0},w_{0}\right)$ are known to the encoder. Clearly, the values of $H(V^{n})$ under the Case (I) formulation are fundamentally different from the values of $H\left(V^{n}|s\right),S_{1}=s$ under the Case (II) formulation. Consequently, in general, $C_{n}^{fb}\left(\kappa \right)$ given by (75) is fundamentally different from $C_{n}^{fb}\left(\kappa ,s\right)$, i.e., it corresponds to a fixed initial state $S_{1}=s$, known to the encoder and the decoder, and to the channel input distribution.*
*(e) From parts (a–d), the characterization of feedback capacity for the stationary ARMA(a,c), a∈[−1,1],c∈(−1,1) given in [4] (Theorem 6.1, $C_{FB}$) (which is derived based on [4] (Lemma 6.1)) presupposed the encoder and the decoder assumed knowledge of $S_{1}=S_{1}^{s}=s$,*

*In fact, the formulas of capacity in [4,7,8] use $H\left(V^{n}|v_{0},w_{0}\right)=\frac{1}{2}\sum_{t=1}^{n}\log\left(2\pi eK_{W}\right)$.*

In the next proposition, we state conditions for the stable realizations of Example 2(a), i.e., AR(a,c),a∈[−1,1],c∈(−1,1) to be asymptotically stationary, and for the realizations of Example 2(b,c) to be stationary. We should emphasize that for stationary noise, we need to determine the initial conditions of the generalized Kalman filter of Lemma 1 to correspond to the stationary noise.

**Proposition 1.**  *Asymptotically stationary and stationary ARMA(a,c) noises of Example 2*

*(a) The realization of the double-sided ARMA(a,c),a∈[−1,1],c∈(−1,1) noise of Example 2(b) is stationary if the following conditions hold:*
(133) $$\begin{array}{cc} \; & d_{11}\overset{▵}{=}cov\left(S_{t},S_{t}\right)=K_{S_{t}},\;d_{12}\overset{▵}{=}cov\left(S_{t},V_{t}\right)=K_{S_{t},V_{t}},\\ \; & d_{22}\overset{▵}{=}cov\left(V_{t},V_{t}\right)=K_{V_{t}},\;are\;constant\;\forall t\in Z. \end{array}$$
  *where the constants $\left(d_{11},d_{12},d_{22}\right)$ are given by*
(134) $$\begin{array}{cc} \; & d_{11}=\frac{K_{W}}{1-c^{2}},\;d_{12}=\frac{\left(c-a\right)K_{W}}{1-c^{2}},\;d_{22}=\frac{{\left(c-a\right)}^{2}K_{W}}{1-c^{2}}+K_{W}. \end{array}$$
  *Similarly, the one-sided ARMA(a,c),a∈[−1,1],c∈(−1,1) noise of Example 2(c) is stationary if the above equations hold $\forall t\in Z_{+}\overset{▵}{=}\left\{1,2,\ldots \right\}$.*
*(b) The realization of the ARMA(a,c) noise of Example 2(a) is asymptotically stationary if a∈[−1,1],c∈(−1,1).*
*(c) For the stationary realization of part (a), the optimal conditional variance and conditional mean of $S_{t}$ from $\left(V_{0},V_{1},V_{2},\ldots ,V_{t-1}\right)$, i.e., $\Sigma_{t}\overset{▵}{=}cov(S_{t},S_{t}|V^{t-1},V_{0}),{\hat{S}}_{t}\overset{▵}{=}E\left\{S_{t}|V^{t-1},V_{0}\right\}$ are defined by the generalized Kalman filter given by*
(135) $$\begin{array}{cc} \; & {\hat{S}}_{t+1}=c{\hat{S}}_{t}+\left({\left(c\right)}^{2}\Sigma_{t}+K_{W}\right){\left(K_{W}+{\left(c-a\right)}^{2}\Sigma_{t}\right)}^{-1}\left(V_{t}-\left(c-a\right){\hat{S}}_{t}\right),\;t=1,2,\ldots , \end{array}$$
(136) $$\begin{array}{cc} \; & \Sigma_{t+1}={\left(c\right)}^{2}\Sigma_{t}+K_{W_{t}}-{\left(c\left(a-c\right)\Sigma_{t}+K_{W}\right)}^{2}{\left(K_{W}+{\left(c(a-c)\right)}^{2}\Sigma_{t}\right)}^{-1} \end{array}$$
  *initialized at the initial data*
(137) $$\begin{array}{cc} \; & {\hat{S}}_{1}\overset{▵}{=}E\left\{S_{1}|V_{0}\right\}=\frac{cd_{12}+K_{W}}{d_{22}}V_{0}, \end{array}$$
(138) $$\begin{array}{cc} \; & \Sigma_{1}\overset{▵}{=}cov\left(S_{1},S_{1}|V_{0}\right)=d_{11}-\frac{{\left(d_{12}\right)}^{2}}{d_{22}}. \end{array}$$
  *(i) If the conditioning information is $\left(V_{-N},\ldots ,V_{0},V_{1},V_{2},\ldots ,V_{t-1}\right)$, then the generalized Kalman filters (135) and (136) still hold and are initialized at the initial data*
(139) $$\begin{array}{cc} \; & {\hat{S}}_{-N+1}=\frac{cd_{12}+K_{W}}{d_{22}}V_{-N}, \end{array}$$
(140) $$\begin{array}{cc} \; & \Sigma_{-N+1}=d_{11}-\frac{{\left(d_{12}\right)}^{2}}{d_{22}}. \end{array}$$
  *(ii) If the inital data $V_{0}$ are not available, then the generalized Kalman filter is initialized at initial data ${\hat{S}}_{1}=0$, $\Sigma_{1}=cov\left(S_{1},S_{1}\right)=d_{11}$.*

**Proof.**  See Appendix A.2.  □

**Remark 6.**  *Consider the stationary double-sided or one-sided ARMA(a,c),a∈[−1,1],c∈(−1,1) of Example 2. From Proposition 1 and, in particular, the initial data ${\hat{S}}_{1},\Sigma_{1}$ stated in (137) and (138), it is clear that even if the encoder and the decoder know the initial state $V_{0}$. Thus, $H\left(V^{n}|v_{0}\right)\neq \frac{1}{2}\sum_{t=1}^{n}\log\left(2\pi eK_{W}\right)$. In this case, the value of $C_{n}^{fb}\left(\kappa ,v_{0}\right)$ defined by (81) is fundamentally different from the formulation in [4,7,8], leading to the characterization of feedback capacity [4] (Theorem 6.1).*

In the next corollary, we further clarify the difference between Case (I) formulation and Case (II) formulation, by stating the analog of Theorem 1 for the code of Definition 3, i.e., when $S_{1}=S_{1}^{s}=s$ is fixed.

**Corollary 3.**  *n–FTFI capacity for feedback code of Definition 3*

*Consider the time-varying AGN channel defined by (1), driven by a noise with the PO-SS realization of Definition 2, and the code of Definition 3, with initial state $S_{1}=S_{1}^{s}=s$ being fixed.*
*Then, the following hold.*
*(a) The n–FTFI capacity $C_{n}^{fb}\left(\kappa ,s\right)$ is given by*
(141) $$\begin{array}{cc} C_{n}^{fb}\left(\kappa ,s\right)\overset{▵}{=} & \underset{\frac{1}{n}E_{s}^{\bar{P}}\left\{\sum_{t=1}^{n}{\left(X_{t}\right)}^{2}|S_{1}\right\}\leq \kappa }{\sup}H^{\bar{P}}\left(Y^{n}|s\right)-H\left(V^{n}|s\right). \end{array}$$
(142) $$\begin{array}{cc} X_{t}= & \Gamma^{0}s+\sum_{j=1}^{t-1}\Gamma_{t,j}^{1}V_{j}+\sum_{j=1}^{t-1}\Gamma_{t,j}^{2}Y_{j}+Z_{t},\;t=1,\ldots ,n. \end{array}$$
  *where the supremum is over all $(\Gamma^{0},\Gamma_{t,j}^{1},\Gamma_{t,j}^{2},K_{Z_{t}}),j=1,\ldots ,t-1,t=1,\ldots ,n$ of the realization of $X^{n}$, which induces the distribution ${\bar{P}}_{t}\left(dx_{t}|v^{t-1},y^{t-1},s\right),t=1,\ldots ,n$, and all statements of Theorem 1 and Lemma 1 hold, with the conditional distributions, expectations, and entropies replaced by the corresponding expressions with a fixed $S_{1}=S_{1}^{s}=s$.*
*(b) A necessary condition for Condition 2 of Section 1.1 to hold is as follows:*
*(i) $N_{t}W_{t}$ uniquely defines $C_{t+1}B_{t}W_{t},\forall t$.*
*Moreover, if (i) holds, then the entropy $H(V^{n}|s)$ of part (a) is given by*
(143) $$\begin{array}{c} H\left(V^{n}|s\right)=\frac{1}{2}\sum_{t=1}^{n}\log\left(2\pi eN_{t}K_{W_{t}}N_{t}^{T}\right). \end{array}$$
  *The stable, time-invariant PO-SS realization of Definition 2, which is considered in [4,7], satisfies $W_{t}:\Omega \to R,N_{t}=1,\forall t$, i.e., $n_{w}=1$. Moreover, for this realization, (i) always holds.*

**Proof.**  See Appendix A.3.  □

In the next remark, we illustrate that $H(V^{n}|s)$ given by (143) follows directly from Lemma 1 by fixing $S_{1}=S_{1}^{s}=s$ and assuming that $N_{t}W_{t}$ uniquely defines $C_{t+1}B_{t}W_{t},\forall t$.

**Remark 7.**  *The n–FTFI capacity for code of Definition 1 versus code of Definition 3.*

*Consider the generalized Kalman filter of the PO-SS noise realization, of Lemma 1, and assume the following:*

*(i) The initial state of the noise $S_{1}$ is known, i.e., $S_{1}=S_{1}^{s}=s$ or $S_{1}=S_{1}^{s}=s=0$, and $N_{t}W_{t}$ uniquely defines $C_{t+1}B_{t}W_{t},\forall t$.*

*Then, all statements of Lemma 1 hold, by replacing $\left(\Sigma_{t},{\hat{S}}_{t}\right)$ with $\left(\Sigma_{t}^{s},{\hat{S}}_{t}^{s}\right)$ for t=1,2,…. Since $\Sigma_{t}^{s}$ satisfies the generalized DRE (95) with initial condition $\Sigma_{1}^{s}=0$, then it is easy to deduce that $\Sigma_{t}^{s}=0$ for t=1,2,…,n is a solution. By substituting $\Sigma_{t}^{s}=0,t=1,2,\ldots ,n$ in (98), we obtain (143), as expected, which is precisely the entropy of the noise that appeared in [4,7].*

*On the other hand, for the code of Definition 1, by Theorem 1(d), the right-hand side of the n–FTFI capacity $C_{n}^{fb}\left(\kappa \right)$ involves $H(V^{n})$, which is computed using the generalized Kalman filter of Lemma 1.*

### 2.3. A Sufficient Statistic Approach to the Characterization of *n*–FTFI Capacity of AGN Channels Driven by PO-SS Noise Realizations

The characterization of the *n*–FTFI capacity via (34) (which is equivalently given in Theorem 1(d)), although compactly represented, is not very practical because the input process $X^{n}$ is not expressed in terms of a *sufficient statistic* that summarizes the information of the channel input strategy [39].

In this section, we wish to identify a *sufficient statistic* for the input process $X_{t}$, given by (68), called the *state of the input*, which summarizes the information contained in $\left(V^{t-1},Y^{t-1}\right)$. It will then become apparent that the characterization of the *n*–FTFI capacity for the Cover and Pombra formulation and code of Definition 1 can be expressed as a functional of *two generalized matrix DREs*.

First, we invoke Theorem 1 and Lemma 1 to show that for each time *t*, $X_{t}$ is expressed as

(144) $$\begin{array}{cc} \; & X_{t}=\Lambda_{t}\left({\hat{S}}_{t}-E\left\{{\hat{S}}_{t}|Y^{t-1}\right\}\right)+Z_{t},\;t=1,\ldots ,n, \end{array}$$

(145) $$\begin{array}{cc} \; & {\hat{S}}_{t}\overset{▵}{=}E\left\{S_{t}|V^{t-1}\right\},\;{\hat{\hat{S}}}_{t}\overset{▵}{=}E\left\{{\hat{S}}_{t}|Y^{t-1}\right\} \end{array}$$

which means, at each time *t*, the state of the channel input process $X_{t}$ is $\left({\hat{S}}_{t},{\hat{\hat{S}}}_{t}\right)$. We show that ${\hat{\hat{S}}}_{t}$ satisfies another generalized Kalman filter recursion.

Now, we prepare to prove (144) and the main theorem. We start with preliminary calculations.

(146) $$\begin{array}{cc} P\left\{Y_{t}\in dy|Y^{t-1},X^{t}\right\}= & P_{t}\left(dy|X_{t},V^{t-1}\right),\;\;t=2,\ldots ,n,\;\text{by channel definition} \end{array}$$

(147) $$\begin{array}{cc} = & P_{t}\left(dy|X_{t},V^{t-1},{\hat{S}}^{t}\right),\;\mathrm{by}\;{\hat{S}}_{t}=E\left\{S_{t}|V^{t-1}\right\} \end{array}$$

(148) $$\begin{array}{cc} = & P_{t}\left(dy|X_{t},V^{t-1},{\hat{S}}_{t},{\hat{I}}^{t-1}\right),\;\mathrm{by}\;\text{(90)},\;i.e.,V_{t}=C_{t}{\hat{S}}_{t}+{\hat{I}}_{t} \end{array}$$

(149) $$\begin{array}{cc} = & P_{t}\left(dy|X_{t},{\hat{S}}_{t}\right),\;\mathrm{by}\;Y_{t}=X_{t}+V_{t}=X_{t}+C_{t}{\hat{S}}_{t}+{\hat{I}}_{t},\text{(91)}. \end{array}$$

At t=1, we also have $P\left\{Y_{1}\in dy|X_{1}\right\}=P_{1}\left(dy|X_{1}\right)$. By (149), it follows that the conditional distribution of $Y_{t}$ given $Y^{t-1}=y^{t-1}$ is

(150) $$\begin{array}{cc} P_{t}\left(dy_{t}|y^{t-1}\right)= & \int P_{t}\left(dy|x_{t},{\hat{s}}_{t}\right)P_{t}\left(dx_{t}|{\hat{s}}_{t},y^{t-1}\right)P_{t}\left(d{\hat{s}}_{t}|y^{t-1}\right),\;t=2,\ldots ,n, \end{array}$$

(151) $$\begin{array}{cc} P_{1}\left(dy_{1}\right)= & \int P_{1}\left(dy|x_{t},{\hat{s}}_{1}\right)P_{1}\left(dx_{1}|{\hat{s}}_{1}\right)P_{1}\left(d{\hat{s}}_{1}\right). \end{array}$$

From the above distributions, at each time *t*, the distribution of $X_{t}$ conditioned on $\left(V^{t-1},Y^{t-1}\right)$, given in Theorem 1, is also expressed as a linear functional of $\left({\hat{S}}_{t},Y^{t-1}\right)$, for t=1,…,n.

The next theorem further shows that for each *t*, the dependence of $X_{t}$ on $Y^{t-1}$ is expressed in terms of $E\left\{{\hat{S}}_{t}|Y^{t-1}\right\}$ for t=1,…,n, and this dependence gives rise to an equivalent sequential characterization of the Cover and Pombra *n*–FTFI capacity, $C_{n}^{fb}\left(\kappa \right)$.

**Theorem 3.**  *Equivalent characterization of n–FTFI capacity $C_{n}^{fb}\left(\kappa \right)$ for PO-SS noise realizations*

*Consider the time-varying AGN channel defined by (1), driven by a noise with the PO-SS realization of Definition 2, and the code of Definition 1. Also consider the generalized Kalman filter of Lemma 1.*
*Define the conditional covariance and conditional mean of ${\hat{S}}_{t}$, given $Y^{t-1}$, by*
(152) $$\begin{array}{cc} K_{t}\overset{▵}{=} & cov\left({\hat{S}}_{t},{\hat{S}}_{t}|Y^{t-1}\right)=E\left\{\left({\hat{S}}_{t}-{\hat{\hat{S}}}_{t}\right){\left({\hat{S}}_{t}-{\hat{\hat{S}}}_{t}\right)}^{T}|Y^{t-1}\right\},\;\;t=2,\ldots ,n, \end{array}$$
(153) $$\begin{array}{cc} {\hat{\hat{S}}}_{t}\overset{▵}{=} & E\left\{{\hat{S}}_{t}|Y^{t-1}\right\},\;{\hat{\hat{S}}}_{1}\overset{▵}{=}\mu_{S_{1}},\;K_{1}\overset{▵}{=}0. \end{array}$$
  *Then, the following hold.*
*(a) An equivalent characterization of the n–FTFI capacity $C_{n}^{fb}\left(\kappa \right)$, defined by (34) and (35), is*
(154) $$\begin{array}{c} C_{n}^{fb}\left(\kappa \right)=\underset{P_{\left[1,n\right]}^{\hat{S}}\left(\kappa \right)}{\sup}\sum_{t=1}^{n}H\left(Y_{t}|Y^{t-1}\right)-H\left(V^{n}\right) \end{array}$$
  *where $\left(X^{n},Y^{n}\right)$ is jointly Gaussian, and*
(155) $$\begin{array}{cc} \; & H\left(V^{n}\right)\;is\;the\;entropy\;of\;V^{n}\;given\;in\;Lemma\;1,i.e.,\text{(98)}, \end{array}$$
(156) $$\begin{array}{cc} \; & {\hat{I}}^{n}\;is\;the\;innovations\;process\;of\;V^{n}\;given\;in\;Lemma\;1; \end{array}$$
(157) $$\begin{array}{cc} \; & Y_{t}=X_{t}+V_{t},\;t=1,\ldots ,n; \end{array}$$
(158) $$\begin{array}{cc} \; & V_{t}=C_{t}{\hat{S}}_{t}+{\hat{I}}_{t}; \end{array}$$
(159) $$\begin{array}{cc} \; & P_{t}\left(dy_{t}|y^{t-1}\right)=\int P_{t}\left(dy|x_{t},{\hat{s}}_{t}\right)P_{t}\left(dx_{t}|{\hat{s}}_{t},y^{t-1}\right)P_{t}\left(d{\hat{s}}_{t}|y^{t-1}\right),\;t=2,\ldots ,n; \end{array}$$
(160) $$\begin{array}{cc} \; & P_{1}\left(dy_{1}\right)=\int P_{1}\left(dy|x_{t},{\hat{s}}_{1}\right)P_{1}\left(dx_{1}|{\hat{s}}_{1}\right)P_{1}\left(d{\hat{s}}_{1}\right); \end{array}$$
(161) $$\begin{array}{cc} \; & P_{t}\left(dy_{t}|y^{t-1}\right)\in N\left(\mu_{Y_{t}|Y^{t-1}},K_{Y_{t}|Y^{t-1}}\right); \end{array}$$
(162) $$\begin{array}{cc} \; & \mu_{Y_{t}|Y_{t-1}}\;is\;linear\;in\;Y^{t-1}\;and\;K_{Y_{t}|Y^{t-1}}\;is\;nonrandom; \end{array}$$
(163) $$\begin{array}{cc} \; & P_{t}\left(dx_{t}|{\hat{s}}_{t},y^{t-1}\right)\in N\left(\mu_{X_{t}|{\hat{S}}_{t},Y^{t-1}},K_{X_{t}|{\hat{S}}_{t},Y^{t-1}}\right); \end{array}$$
(164) $$\begin{array}{cc} \; & \mu_{X_{t}|{\hat{S}}_{t},Y^{t-1}}\;is\;linear\;in\;\left({\hat{S}}_{t},Y^{t-1}\right)\;and\;K_{X_{t}|{\hat{S}}_{t},Y^{t-1}}is\;nonrandom; \end{array}$$
(165) $$\begin{array}{cc} \; & P_{\left[1,n\right]}^{\hat{S}}\left(\kappa \right)\overset{▵}{=}\left\{P_{t}\left(dx_{t}|{\hat{s}}_{t},y^{t-1}\right),t=1,\ldots ,n:\frac{1}{n}E\left(\sum_{t=1}^{n}{\left(X_{t}\right)}^{2}\right)\leq \kappa \right\}. \end{array}$$
*(b) The optimal jointly Gaussian process $\left(X^{n},Y^{n}\right)$ of part (a) is represented as a function of a sufficient statistic by*
(166) $$\begin{array}{cc} \; & X_{t}=\Lambda_{t}\left({\hat{S}}_{t}-{\hat{\hat{S}}}_{t}\right)+Z_{t},\;t=1,\ldots ,n, \end{array}$$
(167) $$\begin{array}{cc} \; & Z_{t}\in N\left(0,K_{Z_{t}}\right)\;independent\;of\;\left(X^{t-1},V^{t-1},{\hat{S}}^{t},\hat{{\hat{S}}^{t}},{\hat{I}}^{t},Y^{t-1}\right),\;t=1,\ldots ,n; \end{array}$$
(168) $$\begin{array}{cc} \; & {\hat{I}}_{t}\in N\left(0,K_{{\hat{I}}_{t}}\right)\;independent\;of\;\left(X^{t-1},V^{t-1},{\hat{S}}^{t},Y^{t-1},\hat{{\hat{S}}^{t}}\right),\;t=1,\ldots ,n; \end{array}$$
(169) $$\begin{array}{cc} \; & Y_{t}=\Lambda_{t}\left({\hat{S}}_{t}-{\hat{\hat{S}}}_{t}\right)+Z_{t}+V_{t},\;t=1,\ldots ,n; \end{array}$$
(170) $$\begin{array}{cc} \; & \;\;=\Lambda_{t}\left({\hat{S}}_{t}-{\hat{\hat{S}}}_{t}\right)+C_{t}{\hat{S}}_{t}+{\hat{I}}_{t}+Z_{t}; \end{array}$$
(171) $$\begin{array}{cc} \; & \frac{1}{n}E\left\{\sum_{t=1}^{n}{\left(X_{t}\right)}^{2}\right\}=\frac{1}{n}\sum_{t=1}^{n}\left(\Lambda_{t}K_{t}\Lambda_{t}^{T}+K_{Z_{t}}\right). \end{array}$$
  *where $\Lambda_{t}$ is nonrandom.*
*The conditional mean and covariance, ${\hat{\hat{S}}}_{t}$ and $K_{t}$, are given by generalized Kalman filter equations, as follows:*
*(i) ${\hat{\hat{S}}}_{t}$ satisfies the Kalman filter recursion*
(172) $$\begin{array}{cc} \; & {\hat{\hat{S}}}_{t+1}=A_{t}{\hat{\hat{S}}}_{t}+F_{t}\left(\Sigma_{t},K_{t}\right)I_{t},\;{\hat{\hat{S}}}_{1}=\mu_{S_{1}}; \end{array}$$
(173) $$\begin{array}{cc} \; & F_{t}\left(\Sigma_{t},K_{t}\right)\overset{▵}{=}\left(A_{t}K_{t}{\left(\Lambda_{t}+C_{t}\right)}^{T}+M_{t}\left(\Sigma_{t}\right)K_{{\hat{I}}_{t}}\right){\left\{K_{{\hat{I}}_{t}}+K_{Z_{t}}+\left(\Lambda_{t}+C_{t}\right)K_{t}{\left(\Lambda_{t}+C_{t}\right)}^{T}\right\}}^{-1}; \end{array}$$
(174) $$\begin{array}{cc} \; & I_{t}\overset{▵}{=}Y_{t}-E\left\{Y_{t}|Y^{t-1}\right\}=Y_{t}-C_{t}{\hat{\hat{S}}}_{t}=\left(\Lambda_{t}+C_{t}\right)\left({\hat{S}}_{t}-{\hat{\hat{S}}}_{t}\right)+{\hat{I}}_{t}+Z_{t},\;t=1,\ldots ,n; \end{array}$$
(175) $$\begin{array}{cc} \; & I_{t}\in N\left(0,K_{I_{t}}\right),\;t=1,\ldots ,n\;is\;an\;orthogonal\;innovations\;process,\\ \; & \;\;i.e.,\;I_{t}\;is\;independent\;of\;I_{s},\;for\;all\;t\neq s,\;and\;I_{t}\;is\;independent\;of\;V^{t-1}; \end{array}$$
(176) $$\begin{array}{cc} \; & K_{Y_{t}|Y^{t-1}}=K_{I_{t}}\overset{▵}{=}cov\left(I_{t},I_{t}\right)=\left(\Lambda_{t}+C_{t}\right)K_{t}{\left(\Lambda_{t}+C_{t}\right)}^{T}+K_{{\hat{I}}_{t}}+K_{Z_{t}}; \end{array}$$
(177) $$\begin{array}{cc} \; & K_{{\hat{I}}_{t}}\;given\;by\;\text{(92)}. \end{array}$$
*(ii) The error ${\hat{E}}_{t}\overset{▵}{=}{\hat{S}}_{t}-{\hat{\hat{S}}}_{t}$ satisfies the recursion*
$$\begin{array}{cc} {\hat{E}}_{t+1}= & F_{t}^{CL}\left(\Sigma_{t},K_{t}\right){\hat{E}}_{t}+\left(M_{t}\left(\Sigma_{t}\right)-F_{t}\left(\Sigma_{t},K_{t}\right)\right){\hat{I}}_{t} \end{array}$$
(178) $$\begin{array}{cc} \; & -F_{t}\left(\Sigma_{t}.K_{t}\right)Z_{t},\;{\hat{E}}_{1}={\hat{S}}_{1}-{\hat{\hat{S}}}_{1}=0,\;t=1,\ldots ,n; \end{array}$$
(179) $$\begin{array}{cc} F_{t}^{CL}(\Sigma_{t}, & K_{t})\overset{▵}{=}A_{t}-F_{t}\left(\Sigma_{t},K_{t}\right)\left(\Lambda_{t}+C_{t}\right). \end{array}$$
  *(iii) $K_{t}=E\left\{{\hat{E}}_{t}{\hat{E}}_{t}^{T}\right\}$ satisfies the generalized DRE*
(180) $$\begin{array}{cc} \; & K_{t+1}=A_{t}K_{t}A_{t}^{T}-\left(A_{t}K_{t}{\left(\Lambda_{t}+C_{t}\right)}^{T}+M_{t}\left(\Sigma_{t}\right)K_{{\hat{I}}_{t}}\right)(K_{{\hat{I}}_{t}}+K_{Z_{t}}\\ \; & +\left(\Lambda_{t}+C_{t}\right)K_{t}{\left(\Lambda_{t}+C_{t}\right)}^{T}{)}^{-1}{\left(A_{t}K_{t}{\left(\Lambda_{t}+C_{t}\right)}^{T}+M_{t}\left(\Sigma_{t}\right)K_{{\hat{I}}_{t}}\right)}^{T}\\ \; & +M_{t}\left(\Sigma_{t}\right)K_{{\hat{I}}_{t}}{\left(M_{t}\left(\Sigma_{t}\right)\right)}^{T},\;K_{t}⪰0,\;t=1,\ldots ,n,\;K_{1}=0. \end{array}$$
  *(c) An equivalent characterization of the n–FTFI capacity $C_{n}^{fb}\left(\kappa \right)$, defined by (33) and (34), using the sufficient statistics of part (b) is*
(181) $$\begin{array}{cc} C_{n}^{fb}\left(\kappa \right)= & \underset{\left(\Lambda_{t},K_{Z_{t}}\right),t=1,\ldots ,n:\;\frac{1}{n}E\left\{\sum_{t=1}^{n}{\left(X_{t}\right)}^{2}\right\}\leq \kappa }{\sup}\frac{1}{2}\sum_{t=1}^{n}\log\frac{K_{Y_{t}|Y^{t-1}}}{K_{V_{t}|V^{t-1}}} \end{array}$$
(182) $$\begin{array}{cc} = & \underset{\left(\Lambda_{t},K_{Z_{t}}\right),t=1,\ldots ,n:\;\frac{1}{n}E\left\{\sum_{t=1}^{n}{\left(X_{t}\right)}^{2}\right\}\leq \kappa }{\sup}\frac{1}{2}\sum_{t=1}^{n}\log\frac{K_{I_{t}}}{K_{{\hat{I}}_{t}}}\\ = & \underset{\left(\Lambda_{t},K_{Z_{t}}\right),t=1,\ldots ,n:\;\frac{1}{n}\sum_{t=1}^{n}\left(\Lambda_{t}K_{t}\Lambda_{t}^{T}+K_{Z_{t}}\right)\leq \kappa }{\sup}\frac{1}{2}\{ \end{array}$$
(183) $$\begin{array}{cc} \; & \sum_{t=1}^{n}\log\left(\frac{\left(\Lambda_{t}+C_{t}\right)K_{t}{\left(\Lambda_{t}+C_{t}\right)}^{T}+K_{{\hat{I}}_{t}}+K_{Z_{t}}}{K_{{\hat{I}}_{t}}}\right)\}. \end{array}$$

**Proof.**  See Appendix A.4.  □

**Remark 8.**  *On the characterization of n–FTFI capacity of Theorem 3*

*The characterization of the n–FTFI capacity $C_{n}^{fb}\left(\kappa \right)$ given by (183) involves the generalized matrix DRE $K_{t}$, which is also a functional of the generalized matrix DRE $\Sigma_{t}$ of the error covariance of the state $S^{n}$ from the noise output $V^{n}$. This feature is not part of the analysis in [4] and recent studies [4,7,9,11,12].*

The next corollary follows directly from Theorem 3 as a degenerate case.

**Corollary 4.**  *Equivalent characterization of n–FTFI capacity $C_{n}^{fb}\left(\kappa ,s\right)$ for PO-SS noise realizations Consider the time-varying AGN channel defined by (1), driven by a noise with the PO-SS realization of Definition 2, and the code of Definition 3, with initial state $S_{1}=S_{1}^{s}=s$ fixed, and replace (152) and (153) by*

(184) $$\begin{array}{cc} K_{t}=K_{t}^{s}\overset{▵}{=} & cov\left({\hat{S}}_{t}^{s},{\hat{S}}_{t}^{s}|Y^{t-1},S_{1}=s\right)=E\left\{\left({\hat{S}}_{t}^{s}-\hat{{\hat{S}}_{t}^{s}}\right){\left({\hat{S}}_{t}^{s}-\hat{{\hat{S}}_{t}^{s}}\right)}^{T}\right\}, \end{array}$$

(185) $$\begin{array}{cc} {\hat{\hat{S}}}_{t}= & \hat{{\hat{S}}_{t}^{s}}\overset{▵}{=}E\left\{{\hat{S}}_{t}^{s}|Y^{t-1},S_{1}=s\right\},\;t=2,\ldots ,n,\;\hat{{\hat{S}}_{1}}=\hat{{\hat{S}}_{1}^{s}}\overset{▵}{=}s,\;K_{1}=K_{1}^{s}=0. \end{array}$$

*Then, the characterization of n–FTFI capacity, (3), is*

(186) $$\begin{array}{cc} \; & C_{n}^{fb}\left(\kappa ,s\right)=\underset{P_{\left[1,n\right]}^{\hat{{\hat{S}}^{s}}}\left(\kappa \right)}{\sup}\sum_{t=1}^{n}H\left(Y_{t}|Y^{t-1},s\right)-H\left(V^{n}|s\right), \end{array}$$

(187) $$\begin{array}{cc} \; & P_{\left[1,n\right]}^{\hat{{\hat{S}}^{s}}}\left(\kappa \right)\overset{▵}{=}\left\{P_{t}\left(dx_{t}|\hat{{\hat{s}}_{t}^{s}},y^{t-1},s\right),t=1,\ldots ,n:\frac{1}{n}E\left(\sum_{t=1}^{n}{\left(X_{t}\right)}^{2}|S_{1}^{s}=s\right)\leq \kappa \right\} \end{array}$$

*where $H(V^{n}|s)$ is given by Corollary 1, and the statements of Theorem 3 hold with the above changes, i.e., (184), (185), and all conditional entropies, distributions, expectations, etc., are defined for fixed $S_{1}=S_{1}^{s}=s$.*

**Proof.**  It is easily verified from the derivation of Theorem 3 by fixing $S_{1}=S_{1}^{s}=s$.  □

**Remark 9.**  *On the characterization of n–FTFI capacity of Corollary 4*

*The characterization of the n–FTFI capacity $C_{n}^{fb}\left(\kappa ,s\right)$ given in Corollary 4 (similar to Theorem 3) involves two generalized matrix DREs, because it does not assume that Conditions 1 and 2 hold. This distinction is not part of the analysis in [4,7,9,11,12].*

### 2.4. Application Examples

In this section, we apply Theorem 3 to specific examples.

First, we consider the application example of the AGN channel driven by the PO-SS$\left(a_{t},c_{t},b_{t}^{1},b_{t}^{2},d_{t}^{1},d_{t}^{2}\right)$ noise.

**Corollary 5.**  *The n–FTFI capacity $C_{n}^{fb}\left(\kappa \right)$ of the AGN channel driven by the PO-SS$(a_{t},c_{t},b_{t}^{1},b_{t}^{2}$, $d_{t}^{1},d_{t}^{2})$ noise is obtained from Lemma 1 and Theorem 3 by using (113).*

**Proof.**  This is easily verified, as in Corollary 2.  □

In the next corollary, we apply Theorem 3 to the stable and unstable ARMA(a,c) noise to obtain the characterization of the *n*–FTFI capacity $C_{n}^{fb}\left(\kappa \right)$ and $C_{n}^{fb}\left(\kappa ,s\right)$. It is then obvious that for the stable ARMA(a,c),a∈[−1,1],c∈(−1,1) noise, the characterization of $C_{n}^{fb}\left(\kappa \right)$ involves two generalized DREs, contrary to the analysis in [4,7,9,11,12], for the same noise model.

**Corollary 6.**  *Characterization of n–FTFI capacity $C_{n}^{fb}\left(\kappa \right)$ for the ARMA(a,c),a∈(−∞,∞),c∈(−∞,∞)*

*Consider the time-varying AGN channel defined by (1) and the code of Definition 1.*
*(a) For the non-stationary ARMA(a,c),a∈(−∞,∞),c∈(−∞,∞) noise of Example 2(a), the characterization of the n–FTFI capacity, $C_{n}^{fb}\left(\kappa \right)$, is*
(188) $$\begin{array}{cc} \; & C_{n}^{fb}\left(\kappa \right)=\underset{\left(\Lambda_{t},K_{Z_{t}}\right),t=1,\ldots ,n:\;\frac{1}{n}\sum_{t=1}^{n}\left({\left(\Lambda_{t}\right)}^{2}K_{t}+K_{Z_{t}}\right)\leq \kappa }{\sup}\frac{1}{2}\sum_{t=1}^{n}\log\left(\frac{{\left(\Lambda_{t}+c-a\right)}^{2}K_{t}+K_{{\hat{I}}_{t}}+K_{Z_{t}}}{K_{{\hat{I}}_{t}}}\right) \end{array}$$
  *subject to the constraints*
$$\begin{array}{cc} K_{t+1}= & {\left(c\right)}^{2}K_{t}+{\left(M_{t}\left(\Sigma_{t}\right)\right)}^{2}K_{{\hat{I}}_{t}}-{\left(cK_{t}\left(\Lambda_{t}+c-a\right)+M_{t}\left(\Sigma_{t}\right)K_{{\hat{I}}_{t}}\right)}^{2} \end{array}$$
(189) $$\begin{array}{cc} \; & \;.{\left(K_{{\hat{I}}_{t}}+K_{Z_{t}}+{\left(\Lambda_{t}+c-a\right)}^{2}K_{t}\right)}^{-1},\;K_{1}=0,\;t=1,\ldots ,n, \end{array}$$
(190) $$\begin{array}{cc} K_{Z_{t}}\geq & 0,\;K_{t}\geq 0,\;c\neq a,\;K_{W}>0,\;t=1,\ldots ,n \end{array}$$
  *and where*
(191) $$\begin{array}{cc} \; & M_{t}\left(\Sigma_{t}\right)\overset{▵}{=}\left(c\Sigma_{t}\left(c-a\right)+K_{W}\right){\left(K_{W}+{\left(c-a\right)}^{2}\Sigma_{t}\right)}^{-1}, \end{array}$$
(192) $$\begin{array}{cc} \; & K_{{\hat{I}}_{t}}={\left(c-a\right)}^{2}\Sigma_{t}+K_{W},\;t=1,\ldots ,n, \end{array}$$
(193) $$\begin{array}{cc} \; & \Sigma_{t+1}={\left(c\right)}^{2}\Sigma_{t}+K_{W}-{\left(c\Sigma_{t}\left(c-a\right)+K_{W}\right)}^{2}{\left(K_{W}+{\left(c-a\right)}^{2}\Sigma_{t}\right)}^{-1},\;t=1,\ldots ,n, \end{array}$$
(194) $$\begin{array}{cc} \; & \Sigma_{1}=K_{S_{1}}=\frac{{\left(c_{0}\right)}^{2}K_{S_{0}}+{\left(a_{0}\right)}^{2}K_{W_{0}}}{{\left(c_{0}-a_{0}\right)}^{2}}. \end{array}$$
  *The optimal jointly Gaussian process $\left(X^{n},Y^{n}\right)$ is obtained from Theorem 3(b) by invoking*
(195) $$\begin{array}{c} A_{t}⟼c,\;C_{t}⟼c-a,\;B_{t}⟼1,\;N_{t}⟼1,\;t=1,2,\ldots ,n. \end{array}$$
  *Special Case. If $\Sigma_{1}=0$ or the initial state is fixed, $S_{1}=S_{1}^{s}=s$, then*
(196) $$\begin{array}{c} \Sigma_{t}=\Sigma_{t}^{s}=0,\;K_{{\hat{I}}_{t}}=K_{W},\;M_{t}\left(\Sigma_{t}\right)=M_{t}\left(\Sigma_{t}^{s}\right)=1,\;t=1,2,\ldots \end{array}$$
  *and $C_{n}^{fb}\left(\kappa \right)$ reduces to*
(197) $$\begin{array}{cc} C_{n}^{fb}\left(\kappa \right)= & C_{n}^{fb}\left(\kappa ,s\right)=\underset{\left(\Lambda_{t},K_{Z_{t}}\right),t=1,\ldots ,n:\;\frac{1}{n}\sum_{t=1}^{n}\left({\left(\Lambda_{t}\right)}^{2}K_{t}^{s}+K_{Z_{t}}\right)\leq \kappa }{\sup}\{\\ \; & \frac{1}{2}\sum_{t=1}^{n}\log\left(\frac{{\left(\Lambda_{t}+c-a\right)}^{2}K_{t}^{s}+K_{W}+K_{Z_{t}}}{K_{W}}\right)\} \end{array}$$
  *subject to the constraints*
(198) $$\begin{array}{cc} \; & K_{t+1}^{s}={\left(c\right)}^{2}K_{t}^{s}+K_{W}-{\left(cK_{t}^{s}\left(\Lambda_{t}+c-a\right)+K_{W}\right)}^{2}\\ \; & .{\left(K_{Z_{t}}+{\left(\Lambda_{t}+c-a\right)}^{2}K_{t}^{s}+K_{W}\right)}^{-1},\;K_{1}^{s}=0,\;\;K_{t}^{s}\geq 0,\;K_{Z_{t}}\geq 0,\;t=1,\ldots ,n. \end{array}$$
  *(This special case is precisely the application example analyzed in [4,7,8]).*

*(b) For the non-stationary AR(c),c∈(−∞,∞) noise of Example 2(c), the characterization of the n–FTFI capacity $C_{n}^{fb}\left(\kappa \right)$ is obtained from part (a) by setting a=0, i.e.,*

(199) $$\begin{array}{cc} \; & C_{n}^{fb}\left(\kappa \right)=\underset{\left(\Lambda_{t},K_{Z_{t}}\right),t=1,\ldots ,n:\;\frac{1}{n}\sum_{t=1}^{n}\left({\left(\Lambda_{t}\right)}^{2}K_{t}+K_{Z_{t}}\right)\leq \kappa }{\sup}\frac{1}{2}\sum_{t=1}^{n}\log\left(\frac{{\left(\Lambda_{t}+c\right)}^{2}K_{t}+{\left(c\right)}^{2}\Sigma_{t}+K_{W}+K_{Z_{t}}}{{\left(c\right)}^{2}\Sigma_{t}+K_{W}}\right) \end{array}$$

*subject to the constraints of $K_{t},\Sigma_{t}$ are the non-negative solutions of the generalized RDEs:*

$$\begin{array}{cc} K_{t+1}= & {\left(c\right)}^{2}K_{t}+{\left(c\right)}^{2}\Sigma_{t}+K_{W}-{\left(cK_{t}\left(\Lambda_{t}+c\right)+{\left(c\right)}^{2}\Sigma_{t}+K_{W}\right)}^{2} \end{array}$$

(200) $$\begin{array}{cc} \; & .{\left({\left(c\right)}^{2}\Sigma_{t}+K_{W}+K_{Z_{t}}+{\left(\Lambda_{t}+c\right)}^{2}K_{t}\right)}^{-1},\;K_{1}=0,\;t=1,\ldots ,n, \end{array}$$

(201) $$\begin{array}{cc} \Sigma_{t+1}= & {\left(c\right)}^{2}\Sigma_{t}+K_{W}-{\left({\left(c\right)}^{2}\Sigma_{t}+K_{W}\right)}^{2}{\left(K_{W}+{\left(c\right)}^{2}\Sigma_{t}\right)}^{-1},\;\Sigma_{1}=K_{S_{1}}=K_{S_{0}}\geq 0. \end{array}$$

*(c) For the non-stationary AR(c),c∈(−∞,∞) noise of Example 2(c), with $\Sigma_{1}=0$ or a fixed initial state $S_{1}=S_{1}^{s}=s$, then (196) holds, i.e., $\Sigma_{t}=\Sigma_{t}^{s}=0,K_{{\hat{I}}_{t}}=K_{W},M_{t}\left(\Sigma_{t}\right)=M_{t}\left(\Sigma_{t}^{s}\right)=1,t=1,2,\ldots$, and $C_{n}^{fb}\left(\kappa \right)$ reduces to*

(202) $$\begin{array}{cc} \; & C_{n}^{fb}\left(\kappa \right)=C_{n}^{fb}\left(\kappa ,s\right)\\ \; & =\underset{\left(\Lambda_{t},K_{Z_{t}}\right),t=1,\ldots ,n:\;\frac{1}{n}\sum_{t=1}^{n}\left({\left(\Lambda_{t}\right)}^{2}K_{t}^{s}+K_{Z_{t}}\right)\leq \kappa }{\sup}\frac{1}{2}\sum_{t=1}^{n}\log\left(\frac{{\left(\Lambda_{t}+c\right)}^{2}K_{t}^{s}+K_{W}+K_{Z_{t}}}{K_{W}}\right) \end{array}$$

*subject to the constraint*

(203) $$\begin{array}{cc} \; & K_{t+1}^{s}={\left(c\right)}^{2}K_{t}^{s}+K_{W}\\ \; & -{\left(cK_{t}^{s}\left(\Lambda_{t}+c\right)+K_{W}\right)}^{2}{\left(K_{W}+K_{Z_{t}}+{\left(\Lambda_{t}+c\right)}^{2}K_{t}^{s}\right)}^{-1},\;K_{1}^{s}=0,\;t=1,\ldots ,n. \end{array}$$

**Proof.**  (a) The first part follows directly from Theorem 3 by using (195). The last part is obtained as follows. If $\Sigma_{1}=0$ or $S_{1}=S_{1}^{s}=s$ is fixed, then, by (193), it follows that $\Sigma_{t}=\Sigma_{t}^{s}=0,\forall t=2,\ldots$. Moreover, by (191) and (192), it follows that $M_{t}\left(\Sigma_{t}\right)=M_{t}\left(\Sigma_{t}^{s}\right)=1,K_{{\hat{I}}_{t}}={\left(c-a\right)}^{2}\Sigma_{t}^{s}+K_{W}=K_{W},t=1,2,\ldots$. By substituting into (188) and (189), we obtain (197) and (180). (b) From part (a), let a=0. Then,

(204) $$\begin{array}{cc} \; & M_{t}\left(\Sigma_{t}\right)=\left({\left(c\right)}^{2}\Sigma_{t}+K_{W}\right){\left(K_{W}+{\left(c\right)}^{2}\Sigma_{t}\right)}^{-1},\;K_{{\hat{I}}_{t}}={\left(c\right)}^{2}\Sigma_{t}+K_{W},\;t=1,\ldots ,n. \end{array}$$

By substituting into the equations of part (a), we obtain (200) and (201). (c) This is a special case of parts (a) and (b).  □

**Remark 10.**  *By Corollary 6(a), it is obvious that if $\Sigma_{1}=0$, i.e., $K_{S_{0}}=K_{W_{0}}=0$, which means $S_{1}=S_{1}^{s}=s$ is fixed, $\left(V_{0},W_{0}\right)=\left(v_{0},w_{0}\right)$ is fixed (and known to the encoder and the decoder)—see (120). Then, $\Sigma_{1}=\Sigma_{1}^{s}=0$ and $C_{n}^{fb}\left(\kappa \right)=C_{n}^{fb}\left(\kappa ,s\right)$, which depends on the initial state $S_{1}=S_{1}^{s}=s$. To ensure that we obtain a large enough n, the rate $\frac{1}{n}C_{n}\left(\kappa ,s\right)$ is independent of s. Moreover, it is necessary to identify conditions for convergence of solutions $K_{t}^{s},t=1,2,\ldots$ of the generalized DRE (180) to a unique limit, $\lim_{n⟶\infty }K_{n}^{s}=K^{\infty }\geq 0$, which does not depend on the initial data $K_{1}^{s}=0$. We address this problem in Section 3.*

### 2.5. Case (II) Formulation: A Degenerate of Case (I) Formulation

Theorem 3 gives the *n*–FTFI capacity for the Case (I) formulation. However, since the Case (II) formulation is a special case of the Case (I) formulation, we expect that we can recover the characterization of the *n*–FTFI capacity for the Case (II) formulation from Theorem 3, i.e., when the code is $\left(s,2^{nR},n\right)$, n=1,2,…, and Conditions 1 and 2 of Section 1.1 hold. We show this in the next corollary.

**Corollary 7.**  *The degenerate n–FTFI capacity $C_{n}^{fb}\left(\kappa \right)$ of Theorem 3 for the Case (II) formulation*

*Consider the time-varying AGN channel defined by (1), driven by a noise with PO-SS realization of Definition 2, and suppose that the following hold:*
*(1) The code is $\left(s,2^{nR},n\right)$, n=1,2,…;*
*(2) Conditions 1 and 2 of Section 1.1 hold.*

*Then, the following hold:*
*(a) Corollary 1 holds, i.e., all statements of Lemma 1 hold with $\left(\Sigma_{t},{\hat{S}}_{t}\right)$ replaced by $\left(\Sigma_{t}^{s},{\hat{S}}_{t}^{s}\right)$ as defined by (102) and (103). In particular, $\left(\Sigma_{t}^{s},{\hat{S}}_{t}^{s}\right)=\left(0,S_{t}^{s}\right)$ for t=1,2,…, and $H\left(V^{n}\right)=H\left(V^{n}|s\right)$ is given by (143).*
*(b) All statements of Theorem 3 hold with $\left(\Sigma_{t},{\hat{S}}_{t}\right)$ replaced by $\left(\Sigma_{t}^{s},{\hat{S}}_{t}^{s}\right)$, as in part (a), and $\left(K_{t},{\hat{\hat{S}}}_{t}\right)$ defined by (152) and (153) reduces to*
(205) $$\begin{array}{ccc} & & K_{t}=K_{t}^{s}=cov\left(S_{t}^{s},S_{t}^{s}|Y^{t-1},S_{1}^{s}=s\right),\; \end{array}$$
(206) $$\begin{array}{ccc} & & {\hat{\hat{S}}}_{t}={\hat{S}}_{t}^{s}=E\left\{S_{t}^{s}|Y^{t-1},S_{1}^{s}=s\right\},\;K_{1}^{s}=0,{\hat{S}}_{1}^{s}=s,\;t=2,\ldots ,n \end{array}$$
  *In particular, the optimal input process $X^{n}$ of Theorem 3(c) degenerates to*
(207) $$\begin{array}{cc} \; & X_{t}=\Lambda_{t}\left(S_{t}^{s}-{\hat{S}}_{t}^{s}\right)+Z_{t},\;X_{1}=Z_{t},\;t=2,\ldots ,n. \end{array}$$
  *(c) The characterization of the n–FTFI capacity, $C_{n}^{fb}\left(\kappa \right)$ of Theorem 3, degenerates to $C_{n}^{fb,S}\left(\kappa ,s\right)$. It is defined by*
(208) $$\begin{array}{cc} C_{n}^{fb}\left(\kappa \right)= & C_{n}^{fb,S}\left(\kappa ,s\right)\overset{▵}{=}\underset{\left(\Lambda_{t},K_{Z_{t}}\right),t=1,\ldots ,n:\;\frac{1}{n}E_{s}\left\{\sum_{t=1}^{n}{\left(X_{t}\right)}^{2}\right\}\leq \kappa }{\sup}\sum_{t=1}^{n}\log\frac{K_{Y_{t}|Y^{t-1},s}}{K_{V_{t}|V^{t-1},s}} \end{array}$$
(209) $$\begin{array}{cc} = & \underset{\left(\Lambda_{t},K_{Z_{t}}\right),t=1,\ldots ,n:\;\frac{1}{n}\sum_{t=1}^{n}\left(\Lambda_{t}K_{t}^{s}\Lambda_{t}^{T}+K_{Z_{t}}\right)\leq \kappa }{\sup}\{\\ \; & \frac{1}{2}\sum_{t=1}^{n}\log\left(\frac{\left(\Lambda_{t}+C_{t}\right)K_{t}^{s}{\left(\Lambda_{t}+C_{t}\right)}^{T}+N_{t}K_{W_{t}}N_{t}^{T}+K_{Z_{t}}}{N_{t}K_{W_{t}}N_{t}^{T}}\right)\}. \end{array}$$
  *$K_{t}=K_{t}^{s}=E_{s}\left\{E_{t}^{s}{\left(E_{t}^{s}\right)}^{T}\right\}$ satisfies the generalized DRE*
(210) $$\begin{array}{cc} \; & K_{t+1}^{s}=A_{t}K_{t}A_{t}^{T}+B_{t}K_{W_{t}}B_{t}^{T}-\left(B_{t}K_{W_{t}}N_{t}^{T}+A_{t}K_{t}^{s}{\left(\Lambda_{t}+C_{t}\right)}^{T}\right)\{N_{t}K_{W_{t}}N_{t}^{T}+K_{Z_{t}}\\ \; & +\left(\Lambda_{t}+C_{t}\right)K_{t}^{s}{\left(\Lambda_{t}+C_{t}\right)}^{T}{\}}^{-1}{\left(B_{t}K_{W_{t}}N_{t}^{T}+A_{t}K_{t}^{s}\left(\Lambda_{t}+C_{t}\right)\right)}^{T},\;K_{t}^{s}⪰0,\;K_{1}^{s}=0,\;t=1,\ldots ,n. \end{array}$$
  *and the statements of parts (a) and (b) hold.*

**Proof.**  (a) The statements about Lemma 1 follow from Remark 7. (b) The statements about Theorem 3 are easily verified by replacing all conditional expectations, distributions, etc., for a fixed initial state $S_{1}=S_{1}^{s}=s$, and by using part (a), i.e., $\left(\Sigma_{t}^{s},{\hat{S}}_{t}^{s}\right)=\left(0,S_{t}^{s}\right)$, t=1,2,…. Part (c) follows from parts (a) and (b).  □

### 2.6. Comments on Past Studies

It is easily verified that Yang, Kavcic and Tatikonda [8] analyzed $C_{n}^{fb}\left(\kappa ,s\right)$, which is defined by (81), under the Case (II) formulation, i.e., Conditions 1 and 2 of Section 1.1 hold, as discussed in the next remark.

**Remark 11.**  *Prior studies on the time-invariant stationary noise of PSD (41)*

*Yang, Kavcic and Tatikonda [8] analyzed the AGN channel driven by a stationary noise with PSD defined by (41) (see [8] (Theorem 1)). The special case of (126) is found in [8] (Section VI.B, Theorem 7).*
*The analysis in [8] presupposed the following formulation:*
*(i) The code is $\left(s,2^{nR},n\right)$, n=1,2,…, where $S_{1}=S_{1}^{s}=s$ is the initial state of the noise, known to the encoder and the decoder, as discussed in Definition 3;*
*(ii) Conditions 1 and 2 of Section 1.1 hold;*
*(iii) The n–FTFI capacity formula is $C_{n}^{fb}\left(\kappa ,s\right)$, defined by (81).*
*We emphasize that in [8] (Section II.C), a specific realization of the PSD is considered to ensure that Conditions 1 and 2 hold, i.e., the analysis in [8] presupposed a stationary noise and the Case (II) formulation.*

Now, we ask the following: Given the PSD of the noise defined by (41), and the double-sided realization [4] (Equation (58)), i.e., the analog of time-invariant version of the PO-SS realization of Definition 2, or its analogous one-sided realization, what are the necessary conditions for the feedback capacity of [4] (Theorem 6.1) to be valid?

The answer to this question is as follows: Conditions 1 and 2 of Section 1.1 are necessary conditions. We show this in the next proposition.

**Proposition 2.**  *Conditions for validity of the feedback capacity characterization of [4] (Theorem 6.1)*

*Consider the AGN channel (1) driven by a stationary noise with PSD defined by (41) with the double-sided or one-sided realization [4] (Equation (58)), (i.e., analog of time invariant of Definition 2).*
*Then, a necessary condition for [4] (Theorem 6.1) to hold is*
(211) $$\begin{array}{c} P_{X_{t}|X^{t-1},Y_{-\infty }^{t-1}}=P_{X_{t}|S^{t},Y_{-\infty }^{t-1}},\;t=1,\ldots , \end{array}$$
  *Further, Conditions 1 and 2 of Section 1.1 are necessary and sufficient for Equality (211) to hold.*

**Proof.**  See Appendix A.5.  □

The next remark is our final observation on prior studies.

**Remark 12.**  *Comparison of Cover and Pombra Characterization and current literature*

*From Corollary 7 and Proposition 2, we have the following:*
*The characterization of feedback capacity given in [4] (Theorem 6.1, $C_{FB}$) corresponds to the Case (II) formulation and not to the Case (I) formulation. Further, the optimization problem of [4] (Theorem 6.1, $C_{FB}$) is precisely the optimization problem investigated in [8] (Section VI), with the additional restriction that the innovations’ part of the channel input is taken to be asymptotically zero in [4] (Theorem 6.1, $C_{FB}$), i.e., see [4] (Lemma 6.1 and comments above it). Recent studies [7,9,11,12] should be read with caution because the results therein often build on [4] (Theorems 4.1 and 6.1).*

## 3. Asymptotic Analysis for Case (I) Formulation

In this section, we address the asymptotic per unit time limit of the *n*–FTFI capacity. Our analysis includes the following:

(1) Fundamental differences of entropy rates of jointly Gaussian stable versus unstable noise processes.

(2) Necessary and/or sufficient conditions for existence of entropy rates of unstable (and stable) $Y^{n},n=1,2,\ldots$, and $V^{n},n=1,2,\ldots$, expressed in terms of detectability and stabilizability or unit circle controllability conditions of generalized DREs [16,17], and asymptotic stationarity of the optimal input process $X^{n},n=1,2,\ldots$ (and output process $Y^{n},n=1,2,\ldots$, if the noise is stable).

This section also reconfirms that, in general, the asymptotic analysis of the *n*–FTFI capacity of a feedback code that depends on the initial state of the channel, i.e., $S_{1}=S_{1}^{s}=s$ is fundamentally different from code that does does not depend on the initial state.

Closed-form expressions of the asymptotic per unit time limit of $C_{n}^{fb}\left(\kappa ,s\right)$ of AGN channels driven by AR(c),c∈(−∞,∞) noise, i.e., stable and unstable, are found in [1].

Closed-form expressions of the asymptotic per unit time limit of $C_{n}^{fb}\left(\kappa \right)$ of AGN channels driven by ARMA(a,c),a∈(−∞,∞),c∈(−∞,∞) noise are found in [3].

We consider the following definition of rate, often used for nonfeedback capacity of stationary processes; however, our formulations does not assume stationarity.

**Definition 4.**  *Per unit time limit of $C_{n}^{fb,o}\left(\kappa \right)$ and $C_{n}^{fb,o}\left(\kappa ,s\right)$*

*Consider the AGN channel defined by (1), driven by the time-invariant PO-SS realization of Definition 2.*
*(a) For the code of Definition 1, define the per unit time limit*
(212) $$\begin{array}{cc} C^{fb,o}\left(\kappa \right) & \overset{▵}{=}\underset{\lim_{n⟶\infty }\frac{1}{n}E\left\{\sum_{t=1}^{n}{\left(X_{t}\right)}^{2}\right\}\leq \kappa }{\sup}\lim_{n⟶\infty }\frac{1}{n}\left\{H\left(Y^{n}\right)-H\left(V^{n}\right)\right\} \end{array}$$
(213) $$\begin{array}{cc} \; & \leq C^{fb}\left(\kappa \right)\overset{▵}{=}\lim_{n⟶\infty }\frac{1}{n}C_{n}^{fb}\left(\kappa \right) \end{array}$$
  *where, for problem $C^{fb,o}\left(\kappa \right)$, the supremum is taken over all asymptotically time-invarinat distributions with feedback $P_{X_{t}|X^{t-1},Y^{t-1}}^{o}=P_{X_{t}|V^{t-1},Y^{t-1}}^{o},t=1,2,\ldots$, such that the limits exists and the supremum exists and it is finite.*
*(b) For the code of Definition 3, i.e., $\left(s,2^{nR},n\right)$, n=1,2,…, with initial state $S_{1}=S_{1}^{s}=s$, $C^{fb,o}\left(\kappa \right)$ is replaced by $C^{fb,o}\left(\kappa ,s\right)$, defined by (212), with differential entropies, conditional expectations, and conditional distributions, defined for fixed $S_{1}^{s}=s$.*

The rate definition, $C^{fb,o}\left(\kappa \right)$, i.e., the interchange of limit and supremum, is consistent with the definition of rates considered in [4,7,9,11,12]. However, unlike [4,7,9,11,12], we treat the general time-invariant, stable and unstable PO-SS noise realization of Definition 2, which is not necessarily stationary or asymptotically stationary.

We should emphasize that, in general, and irrespective of whether the noise is stable or unstable, the entropy rates that appear in (212) and (213) may not exist. To show existence of the limits $C^{fb,o}\left(\kappa \right),C^{fb}\left(\kappa \right)$ and $C^{fb,o}\left(\kappa ,s\right)$, we identify necessary and/or sufficient conditions, using the characterization of Theorem 3, when the channel input strategies are restricted to asymptotically time-invariant strategies $\lim_{n⟶\infty }\Lambda_{n}=\Lambda^{\infty },\lim_{n⟶\infty }K_{Z_{n}}=K_{Z}^{\infty }$. Clearly, by (212), whether the limit as, n⟶∞ and the supremum over channel input distributions exist, depend on the convergence properties of the coupled generalized matrix DREs, $\Sigma_{n},K_{n}$, as n⟶∞.

### 3.1. Entropy Rates of Gaussian Processes

First, we recall the following definition, which is standard and found in many textbooks:

**Definition 5.**  *Entropy rate of continuous-valued random processes*

*Let $X_{t}:\Omega \to R^{n_{z}},n_{x}\in Z_{+}$ be a random process defined on some probability space (Ω,F,P). The entropy rate (differential) is defined by*
(214) $$\begin{array}{c} H_{R}\left(X^{\infty }\right)\overset{▵}{=}\lim_{n⟶\infty }\frac{1}{n}H\left(X_{1},X_{2},\ldots ,X_{n}\right) \end{array}$$
  *when the limit exists.*

The next theorem quantifies the existence of entropy rates of stationary Gaussian processes [16].

**Theorem 4.**  *The entropy rate of stationary zero-mean full-rank Gaussian process [16]*

*Let $X_{t}:\Omega \to R^{n_{x}},n_{x}\in Z_{+},\forall t\in Z_{+}$ be a stationary Gaussian process, with a zero mean, and full-rank covariance of $X^{n}$. Let $H_{t}^{X}$ denote the Hilbert space of RVs generated by $\left\{X_{t}:s\leq t,s,t\in Z_{+}\right\}$, and define the innovations process by*
(215) $$\begin{array}{c} \Sigma_{t}\overset{▵}{=}E\left\{\left(X_{t}-E\left\{X_{t}|H_{t-1}^{X}\right\}\right){\left(X_{t}-E\left\{X_{t}|H_{t-1}^{X}\right\}\right)}^{T}\right\}≻0 \end{array}$$
  *and its limit*
(216) $$\begin{array}{c} \Sigma \overset{▵}{=}\lim_{n⟶\infty }\Sigma_{n} \end{array}$$
  *Then, the entropy rate is given by*
(217) $$\begin{array}{cc} H_{R}\left(X^{\infty }\right)= & \frac{n_{x}}{2}\log\left(2\pi e\right)+\frac{1}{2}\lim_{n⟶\infty }\frac{1}{n}\sum_{t=1}^{n}\log\left|\Sigma_{t}\right| \end{array}$$
(218) $$\begin{array}{cc} = & \frac{n_{x}}{2\pi }\log\left(2\pi e\right)+\frac{1}{2}\log\left|\Sigma \right| \end{array}$$
  *when it exists.*

An application of Theorem 4 is given in the next proposition [15].

**Proposition 3.**  *Entropy rate of Gaussian process described by PSD (41)*

*Let $V_{t},\forall t\in Z_{+}$ be a real, scalar-valued, stationary Gaussian noise with PSD (41), with a corresponding time-invariant stationary realization (similar to Definition 2). Then, the entropy rate is given by*
(219) $$\begin{array}{c} H_{R}\left(V^{\infty }\right)=\frac{1}{2}\log\left(2\pi eK_{W}\right). \end{array}$$

**Proof.**  This is shown in [15] by using the Szego formula and Poisson’s integral formula.  □

The next remark is trivial; it is introduced for a subsequent comparison.

**Remark 13.**  *Let $V_{t},\forall t\in Z_{+}$ be the non-stationary ARMA(a,c),a∈(−∞,∞),c∈(−∞,∞) noise of Example 2. Then, the conditional entropy of $V^{n}$ for fixed initial state $S_{1}=S_{1}^{s}=s$ is given by*

(220) $$\begin{array}{c} H_{R}\left(V^{\infty }|s\right)\overset{▵}{=}\lim_{n⟶\infty }\frac{1}{n}H\left(V^{n}|s\right)=\lim_{n⟶\infty }\frac{1}{n}\sum_{t=1}^{n}\frac{1}{2}\log\left(2\pi eK_{W}\right)=\frac{1}{2}\log\left(2\pi eK_{W}\right). \end{array}$$

The next lemma identifies fundamental conditions for the existence of the entropy rate of the time-varying PO-SS noise realization of Definition 2 (if $S_{1}=S_{1}^{s}=s$ is not fixed) and includes the entropy rate $H_{R}\left(V^{\infty }\right)$ of the non-stationary ARMA(a,c),a∈(−∞,∞),c∈(−∞,∞) noise of Remark 13.

**Lemma 2.**  *Entropy rate of the time-varying PO-SS noise realization of Definition 2*

*Consider the time-varying PO-SS noise realization of Definition 2. Then, the following hold:*
*(a) The joint entropy of $V^{n}$, when it exists, is given by*
(221) $$\begin{array}{c} H\left(V^{n}\right)=\sum_{t=1}^{n}H\left({\hat{I}}_{t}\right)=\frac{1}{2}\sum_{t=1}^{n}\log\left(2\pi eK_{{\hat{I}}_{t}}\right) \end{array}$$
  *where ${\hat{I}}_{t},t=1,\ldots ,n$ is a zero-mean covariance $K_{{\hat{I}}_{t}}\overset{▵}{=}cov\left({\hat{I}}_{t},{\hat{I}}_{t}\right)$, Gaussian orthogonal innovation process of $V^{n}$ that is defined by*
(222) $$\begin{array}{cc} {\hat{I}}_{t}\overset{▵}{=} & \;V_{t}-E\left\{V_{t}|V^{t-1}\right\},\;t=1,\ldots ,n \end{array}$$
  *that is, ${\hat{I}}_{t}$ is independent of ${\hat{I}}_{k},\forall k\neq t$.*
*(b) Suppose that the sequence $K_{{\hat{I}}_{t}},t=1,2,\ldots ,n$ is such that*
(223) $$\begin{array}{c} \lim_{n⟶\infty }K_{{\hat{I}}_{n}}=K_{\hat{I}}^{\infty }≻0. \end{array}$$
  *Then, the entropy rate of $V_{t},\forall t\in Z_{+}$ is given by*
(224) $$\begin{array}{c} H_{R}\left(V^{\infty }\right)=\lim_{n⟶\infty }\frac{1}{n}\sum_{t=1}^{n}H\left({\hat{I}}_{t}\right)=\frac{1}{2}\log\left(2\pi eK_{\hat{I}}^{\infty }\right). \end{array}$$

**Proof.**  See Appendix A.6.  □

**Remark 14.**  *Entropy rate of non-stationary Gaussian noise*

*By Lemma 2, a necessary condition for existence of the entropy rate of non-stationary Gaussian process $V^{n}$ is the convergence of the covariance of the Gaussian orthogonal innovations process of $V^{n}$, i.e., of $K_{{\hat{I}}_{t}}\overset{▵}{=}cov\left({\hat{I}}_{t},{\hat{I}}_{t}\right)$, since $\lim_{n\to \infty }\frac{1}{n}H\left(V^{n}\right)=\lim_{n\to \infty }\frac{1}{n}\sum_{t=1}^{n}H\left({\hat{I}}_{t}\right)$. We can determine such necessary and/or sufficient conditions from the convergence properties of the generalized Kalman filter equations [16,17] of Lemma 1.*

### 3.2. Convergence Properties of Generalized Matrix DREs to AREs

To address the asymptotic properties of estimation errors generated by the recursions of generalized Kalman filters, such as ${\hat{E}}_{t},t=1,2,\ldots$ of Theorem 3, generated by (178), we need to introduce the stabilizing solutions of generalized AREs. The next definition is useful in this respect.

**Definition 6.**  *Stabilizing solutions of generalized matrix AREs*

*Let $\left(A,G,Q,S,R,C\right)\in R^{q\times q}\times R^{q\times k}\times R^{k\times k}\times R^{k\times p}\times R^{p\times p}\times R^{p\times q}$.*
*Define the generalized time-invariant matrix DRE*
(225) $$\begin{array}{cc} \; & P_{t+1}=AP_{t}A^{T}+GQG^{T}-\left(AP_{t}C^{T}+GS\right){\left(R+CP_{t}C^{T}\right)}^{-1}.{\left(AP_{t}C^{T}+GS\right)}^{T},\;P_{1}=given,\\ \; & P_{t}\in S_{+}^{q\times q},\;t=1,\ldots ,\;R=R^{T}≻0,\\ \; & F^{CL}\left(P\right)\overset{▵}{=}A-\left(APC^{T}+GS\right){\left(R+CPC^{T}\right)}^{-1}C. \end{array}$$
  *Moreover, define the corresponding generalized matrix ARE as follows:*
(226) $$\begin{array}{cc} P= & APA^{T}+GQG^{T}-\left(APC^{T}+GS\right){\left(R+CPC^{T}\right)}^{-1}.{\left(APC^{T}+GS\right)}^{T},\;P\in S_{+}^{q\times q}. \end{array}$$
  *A solution $P=P^{T}⪰0$ to the generalized matrix ARE (226), assuming it exists, is called stabilizing if $spec\left(F^{CL}\left(P\right)\right)\in D_{o}$. In this case, we say that $F^{CL}\left(P\right)$ is asymptotically stable, i.e., the eigenvalues of $F^{CL}\left(P\right)$ are stable.*

With respect to any of the above generalized matrices DRE and ARE, we introduce the important notions of detectability, unit circle controllability, and stabilizability. We use these notions to characterize the convergence properties of solutions of generalized matrix DREs, $P_{n}$, as n⟶∞, to a unique symmetric, non-negative, stabilizing solution *P* of the generalized matrix ARE. These notions are used to identify necessary and/or sufficient conditions for the error recursions of generalized Kalman filters, such as ${\hat{E}}_{t},t=1,2,\ldots$ of Theorem 3, generated by (178), to converge in a mean square sense, to a unique limit. However, we should distinguish whether the convergence is uniform for all initial conditions, $P_{1}⪰0$ or only for $P_{1}≻0$.

**Definition 7.**  *Detectability, Stabilizability, and Unit Circle Controllability*

*Consider the generalized matrix ARE of Definition 6 and introduce the matrices*
(227) $$\begin{array}{c} A^{*}\overset{▵}{=}A-GSR^{-1}C,\;B^{*}\overset{▵}{=}Q-SR^{-1}S^{T},\;B^{*}=B^{*,\frac{1}{2}}{\left(B^{*,\frac{1}{2}}\right)}^{T}. \end{array}$$
  *(a) The pair $\left\{A,C\right\}$ is called detectable if there exists a matrix $K\in R^{q\times p}$ such that $spec\left(A-KC\right)\in D_{o}$, i.e., the eigenvalues λ of A−KC lie in $D_{o}$ (stable).*
(b) The pair $\left\{A^{*},GB^{*,\frac{1}{2}}\right\}$ is called unit circle controllable if there exists a $K\in R^{k\times q}$ such that $spec\left(A^{*}-GB^{*,\frac{1}{2}}K\right)\notin \left\{c\in C:|c|=1\right\}$, i.e., all eigenvalues λ of $A^{*}-GB^{*,\frac{1}{2}}K$ are such that |λ|≠1.
(c) The pair $\left\{A^{*},GB^{*,\frac{1}{2}}\right\}$ is called stabilizable if there exists a $K\in R^{k\times q}$ such that $spec\left(A^{*}-GB^{*,\frac{1}{2}}K\right)\in D_{o}$, i.e., all all eigenvalues λ of $A^{*}-GB^{*,\frac{1}{2}}K$ lie in $D_{o}$.
*(d) The pair $\left\{A,C\right\}$ is called observable if the rank condition holds:*
(228) $$\begin{array}{c} rank\left(O\right)=q,\;O\overset{▵}{=}\left[\begin{array}{c} C\\ CA\\ \vdots \\ CA^{q-1} \end{array}\right]. \end{array}$$
  (e) The pair $\left\{A^{*},GB^{*,\frac{1}{2}}\right\}$ is called controllable if the rank condition holds:
(229) $$\begin{array}{c} rank\left(C\right)=q,\;C\overset{▵}{=}\left[\begin{array}{cccc} GB^{*,\frac{1}{2}} & A^{*}GB^{*,\frac{1}{2}} & \ldots & {\left(A^{*}\right)}^{q-1}GB^{*,\frac{1}{2}} \end{array}\right]. \end{array}$$

**Remark 15.**  The following are well known [16]. If the pair $\left\{A,C\right\}$ is observable, then it is detectable. If the pair $\left\{A^{*},GB^{*,\frac{1}{2}}\right\}$ is controllable, then it is stabilizable.

The next theorem characterizes detectability, unit circle controllability, and stabilizability [17,40].

**Lemma 3**  ([17,40]). *Necessary and sufficient conditions for detectability, unit circle controllability, and stabilizability*

*(a) The pair $\left\{A,C\right\}$ is detectable if and only if there exists no eigenvalue and eigenvector {λ,x}, Ax=λx, such that |λ|≥1 and such that Cx=0.*
  (b) The pair $\left\{A^{*},GB^{*,\frac{1}{2}}\right\}$ is unit circle controllable if and only if there exists no eigenvalue and eigenvector {λ,x}, $x^{T}{\left(A^{*}\right)}^{T}=x^{T}\lambda$, such that |λ|=1 and such that $x^{T}GB^{*,\frac{1}{2}}=0$.
  (c) The pair $\left\{A^{*},GB^{*,\frac{1}{2}}\right\}$ is stabilizable if and only there exists no eigenvalue and eigenvector {λ,x}, $x^{T}{\left(A^{*}\right)}^{T}=x^{T}\lambda$, such that |λ|≥1 and such that $x^{T}GB^{*,\frac{1}{2}}=0$.

In the next theorem, we summarize known results on sufficient and/or necessary conditions for the convergence of solutions $\left\{P_{t},t=1,2,\ldots ,n\right\}$ of the generalized time-invariant DRE (225), as n⟶∞, to a symmetric, non-negative P⪰0, stabilizing solution of the corresponding generalized ARE (226). We recall that the pair $\left\{A,C\right\}$ is detectable, which is a necessary condition forthe convergence of the sequence $\left\{P_{t},t=1,2,\ldots ,n\right\}$ as n⟶∞ to a non-negative *P*, which is a stabilizing solution of a corresponding generalized ARE. However, it is not sufficient. To have a sufficient condition, it is necessary that the pair $\left\{A^{*},GB^{*,\frac{1}{2}}\right\}$ is unit circle controllable; however, the limiting *P* is not necessarily the unique solution of P⪰0 of the generalized ARE. There may be multiple solutions depending on the initial condition $P_{1}⪰0$.

**Theorem 5**  ([16,17]). *Convergence of time-invariant generalized DRE*

*Let $\left\{P_{t},t=1,2,\ldots ,n\right\}$ denote a sequence that satisfies the time-invariant generalized DRE (225) with arbitrary initial condition $P_{1}⪰0$. The following hold:*
  (1) Consider the generalized DRE (225) with a zero initial condition, i.e., $P_{1}=0$, and assume that the pair $\left\{A,C\right\}$ is detectable and that the pair $\left\{A^{*},GB^{*,\frac{1}{2}}\right\}$ is unit circle controllable.
  Then, the sequence $\left\{P_{t}:t=1,2,\ldots ,n\right\}$ that satisfies the generalized DRE (225), with a zero initial condition $P_{1}=0$, converges to P, i.e., $\lim_{n⟶\infty }P_{n}=P$, where P satisfies the generalized matrix ARE (226) if and only if the pair $\left\{A^{*},GB^{*,\frac{1}{2}}\right\}$ is stabilizable.
  (2) Assume that the pair $\left\{A,C\right\}$ is detectable and that the pair $\left\{A^{*},GB^{*,\frac{1}{2}}\right\}$ is unit circle controllable. Then there exists a unique stabilizing solution P⪰0 to the generalized ARE (226), i.e., such that $spec\left(F^{CL}\left(P\right)\right)\in D_{o}$ if and only if $\left\{A^{*},GB^{*,\frac{1}{2}}\right\}$ is stabilizable.
  (3) If {A,C} is detectable and $\left\{A^{*},GB^{*,\frac{1}{2}}\right\}$ is stabilizable, then any solution $P_{t},t=1,2,\ldots ,n$ to the generalized matrix DRE (225) with arbitrary initial condition $P_{1}⪰0$ is such that $\lim_{n⟶\infty }P_{n}=P$, where P⪰0 is the unique solution of the generalized matrix ARE (226) with $spec\left(F^{CL}\left(P\right)\right)\in D_{o}$, i.e., it is stabilizing.
  (4) {A,C} is detectable and $\left\{A^{*},GB^{*,\frac{1}{2}}\right\}$ unit circle controllable, which are necessary and sufficient conditions for any solution $P_{t},t=2,\ldots ,n$ to the generalized DRE (225) to converge, $\lim_{n⟶\infty }P_{n}=P$, from some initial condition $P_{1}⪰0$, where P⪰0 is a stabilizing solution of the generalized ARE (226), but it may not be unique (i.e., (226) may have multiple solutions P⪰0).

**Proposition 4.**  *Generalizations to asymptotic-time invariant coefficients*

*Suppose that the coefficients of the generalized matrix DRE (225), (A,C,G,Q,S) are replaced by $\left(A_{t},C_{t},G_{t},Q_{t},S_{t}\right)$, t=1,2,…,n, and they are asymptotically time-invariant, i.e.,*
(230) $$\begin{array}{c} \lim_{n⟶\infty }\left(A_{n},C_{n},G_{n},Q_{n},S_{n}\right)=\left(A,C,G,Q,S\right). \end{array}$$
  *Then, Theorem 5 remains valid.*

**Proof.**  This is due to the well-known continuity properties of matrix DREs with respect to their coefficients, i.e., the convergence properties are characterized by the limiting pairs, $\left\{A,C\right\}$ and $\left\{A^{*},GB^{*,\frac{1}{2}}\right\}$.  □

### 3.3. Feedback Rates

Now, we return to the feedback rates of Definition 4. The next corollary is an application of Theorem 5 to the generalized Kalman filter of Lemma 1 (for the time-invariant PO-SS realization); it identifies conditions for existence of the entropy rate $H_{R}\left(V^{\infty }\right)$, irrespective of whether the noise is stable or unstable.

**Corollary 8.**  *The entropy rate of PO-SS noise realization based on the generalized Kalman filter*

*Let $\Sigma_{t}^{o}=\Sigma_{t},t=1,2,\ldots$ denote the solution of the generalized matrix DRE (95) of the generalized Kalman filter of Lemma 1 of the time-invariant PO-SS realization of $V^{n}$ of Definition 2, i.e., $\left(A_{t},B_{t},C_{t},N_{t},K_{W_{t}}\right)=\left(A,B,C,N,K_{W}\right),\forall t$, generated by*
$$\begin{array}{cc} \Sigma_{t+1}^{o}= & A\Sigma_{t}^{o}A^{T}+BK_{W}B^{T}-\left(A\Sigma_{t}^{o}C^{T}+BK_{W}N^{T}\right){\left(NK_{W}N^{T}+C\Sigma_{t}^{o}C^{T}\right)}^{-1} \end{array}$$
(231) $$\begin{array}{cc} \; & \;.{\left(A\Sigma_{t}^{o}C^{T}+BK_{W}N^{T}\right)}^{T},\;\Sigma_{t}^{o}⪰0,\;t=1,\ldots ,n,\;\Sigma_{1}^{o}=K_{S_{1}}⪰0. \end{array}$$
(232) $$\begin{array}{cc} M^{CL}\left(\Sigma^{o}\right)\overset{▵}{=} & A-M\left(\Sigma^{o}\right)C,\;M\left(\Sigma^{o}\right)\overset{▵}{=}\left(A\Sigma^{o}C^{T}+B_{t}K_{W}N^{T}\right){\left(NK_{W}N^{T}+C\Sigma^{o}C^{T}\right)}^{-1}. \end{array}$$
  *Let $\Sigma^{\infty }=\Sigma^{\infty ,T}⪰0$ be a solution of the corresponding generalized ARE*
(233) $$\begin{array}{cc} \Sigma^{\infty }= & A\Sigma^{\infty }A^{T}+BK_{W}B^{T}\\ \; & -\left(A\Sigma^{\infty }C^{T}+BK_{W}N^{T}\right){\left(NK_{W}N^{T}+C\Sigma^{\infty }C^{T}\right)}^{-1}{\left(A\Sigma^{\infty }C^{T}+BK_{W}N^{T}\right)}^{T}. \end{array}$$
  *Define the matrices*
(234) $$\begin{array}{cc} \; & GQG^{T}\overset{▵}{=}BK_{W}B^{T},\;GS\overset{▵}{=}BK_{W}N^{T},\;R\overset{▵}{=}NK_{W}N^{T}\;⟹\;G\overset{▵}{=}B,\;Q\overset{▵}{=}K_{W},\;S\overset{▵}{=}K_{W}N^{T}, \end{array}$$
(235) $$\begin{array}{cc} \; & A^{*}\overset{▵}{=}A-BK_{W}N^{T}{\left(NK_{W}N^{T}\right)}^{-1}C,\;B^{*}\overset{▵}{=}K_{W}-K_{W}N^{T}{\left(NK_{W}N^{T}\right)}^{-1}{\left(K_{W}N^{T}\right)}^{T}. \end{array}$$
  *(a) All statements of Theorem 5 hold with (G,Q,S,R) as defined by (234) and (235).*
*In particular, suppose the following:*
*(i) {A,C} is detectable;*
(ii) $\left\{A^{*},GB^{*,\frac{1}{2}}\right\}$ is stabilizable.
*Then, any solution $\Sigma_{t}^{o},t=1,2,\ldots ,n$ to the generalized matrix DRE (231) with arbitrary initial condition $\Sigma_{1}^{o}⪰0$ is such that $\lim_{n⟶\infty }\Sigma_{n}^{o}=\Sigma^{\infty }$, where $\Sigma^{\infty }⪰0$ is the unique and stabilizing solution of the generalized matrix ARE (233), i.e., with $spec\left(M^{CL}\left(\Sigma^{\infty }\right)\right)\in D_{o}$.*
*(b) Suppose that (i) and (ii) hold. The entropy rate of $V^{n}$ is given by*
(236) $$\begin{array}{cc} H_{R}\left(V^{\infty }\right)= & \lim_{n⟶\infty }\frac{1}{2n}\sum_{t=1}^{n}\log\left(2\pi e\left[C\Sigma_{t}^{o}C^{T}+NK_{W}N^{T}\right]\right) \end{array}$$
(237) $$\begin{array}{cc} = & H\left({\hat{I}}_{t}^{\infty }\right)\overset{▵}{=}\frac{1}{2}\log\left(2\pi e\left[C\Sigma^{\infty }C^{T}+NK_{W}N^{T}\right]\right),\;\forall \Sigma_{1}^{o}⪰0,\;\forall t \end{array}$$
  *where*
(238) $$\begin{array}{c} {\hat{I}}_{t}^{\infty }\overset{▵}{=}C\left(S_{t}-{\hat{S}}_{t}^{\infty }\right)+NW_{t}\in N\left(0,C\Sigma^{\infty }C^{T}+NK_{W}N^{T}\right),\;t=1,2,\ldots , \end{array}$$
  *is the stationary Gaussian innovations process, i.e., with $\Sigma_{t}^{o}$ replaced by $\Sigma^{\infty }$, and the entropy rate $H_{R}\left(V^{\infty }\right)$ is independent of the initial data $\Sigma_{1}^{o}⪰0$.*
(c) Suppose in parts (a) and (b), the condition $\left\{A^{*},GB^{*,\frac{1}{2}}\right\}$ is stabilizable is replaced by
(iii) $\left\{A^{*},GB^{*,\frac{1}{2}}\right\}$ is unit circle controllable.
*Then, the statements of parts (a) and (b) hold, for some $\Sigma_{1}^{o}⪰0$, but not for all $\Sigma_{1}^{o}⪰0$. Moreover, ${\hat{I}}_{t}^{\infty }$ is not necessarily a stationary process, i.e., it depends on the value of $\Sigma_{1}^{o}⪰0$.*

**Proof.**  (a) These are direct applications of Theorem 5. (b) This follows from Lemma 2. (c) This is due to Theorem 5(4).  □

Next, we apply Corollary 8 to the non-stationary AR(a,c),a∈(−∞,∞),c∈(−∞,∞) noise.

**Lemma 4.**  *Properties of solutions of DREs and AREs of AR(a,c),a∈(−∞,∞),c∈(−∞,∞) noise and entropy rate $H_{R}\left(V^{\infty }\right)$*

*Consider the AR(a,c),a∈(−∞,∞),c∈(−∞,∞) noise of Example 2(a), and the DRE $\Sigma_{t}^{o}\overset{▵}{=}\Sigma_{t},t=1,\ldots ,n$, generated by Corollary 6(a), i.e.,*
(239) $$\begin{array}{cc} \; & \Sigma_{t+1}^{o}={\left(c\right)}^{2}\Sigma_{t}^{o}+K_{W}-{\left(c\Sigma_{t}^{o}\left(c-a\right)+K_{W}\right)}^{2}{\left(K_{W}+{\left(c-a\right)}^{2}\Sigma_{t}^{o}\right)}^{-1},\;t=1,\ldots ,n, \end{array}$$
(240) $$\begin{array}{cc} \; & \Sigma_{1}^{o}=K_{S_{1}}=\frac{{\left(c_{0}\right)}^{2}K_{S_{0}}+{\left(a_{0}\right)}^{2}K_{W_{0}}}{{\left(c_{0}-a_{0}\right)}^{2}}\geq 0. \end{array}$$
  *where $K_{W}>0,c\neq a$, $K_{S_{0}}\geq 0,K_{W_{0}}\geq 0$. Let $\Sigma^{\infty }\geq 0$ be a solution of the corresponding generalized ARE, as follows:*
(241) $$\begin{array}{cc} \; & \Sigma^{\infty }={\left(c\right)}^{2}\Sigma^{\infty }+K_{W}-{\left(c\Sigma^{\infty }\left(c-a\right)+K_{W}\right)}^{2}{\left(K_{W}+{\left(c-a\right)}^{2}\Sigma^{\infty }\right)}^{-1}. \end{array}$$
  *Then, the detectability and stabilizability pairs are*
(242) $$\begin{array}{c} \left\{A,C\right\}=\left\{c,c-a\right\},\;\left\{A^{*},GB^{*,\frac{1}{2}}\right\}=\left\{a,0\right\}. \end{array}$$
  *and the following hold:*
*(1) The pair {A,C}={c,c−a} is detectable ∀c∈(−∞,∞),a∈(−∞,∞) (the restriction c≠a is always assumed).*
(2) The pair $\left\{A^{*},GB^{*,\frac{1}{2}}\right\}=\left\{a,0\right\}$ is unit circle controllable if and only if |a|≠1 (∀c∈(−∞,∞)).
(3) The pair $\left\{A^{*},GB^{*,\frac{1}{2}}\right\}=\left\{a,0\right\}$ is stabilizable if and only if a∈(−1,1) (∀c∈(−∞,∞)).
(4) Suppose c∈(−∞,∞) and a∈(−∞,∞). The sequence $\left\{\Sigma_{t}^{o},t=1,2,\ldots ,n\right\}$ that satisfies the generalized DRE with any initial condition, $\Sigma_{1}^{o}\geq 0$, converges to $\Sigma^{\infty }$, i.e., $\lim_{n⟶\infty }\Sigma_{n}^{o}=\Sigma^{\infty }$, where $\Sigma^{\infty }\geq 0$ satisfies the ARE (241) if and only if the $\left\{A^{*},GB^{*,\frac{1}{2}}\right\}=\left\{a,0\right\}$ is unit circle controllable, equivalently, |a|≠1. Moreover, the solutions of the quadratic Equation (241), without imposing $\Sigma^{\infty }\geq 0$ are
(243) $$\begin{array}{c} \Sigma^{\infty }=\left\{\begin{array}{cc} 0 & unique,stabilizing,\Sigma^{\infty }\geq 0\;sol.\;of\;\text{(241)}\;for\;c\in \left(-\infty ,\infty \right),\left|a\right|<1\\ \frac{K_{W}\left(a^{2}-1\right)}{{\left(c-a\right)}^{2}}>0 & maximal,stabilizing,\Sigma^{\infty }>0\;sol.\;of\;\text{(241)}\;for\;c\in \left(-\infty ,\infty \right),\left|a\right|>1\\ \frac{K_{W}\left(a^{2}-1\right)}{{\left(c-a\right)}^{2}}<0 & non\text{-}stabilizing,\Sigma^{\infty }<0\;sol.\;of\;\text{(241)}\;for\;c\in \left(-\infty ,\infty \right),\left|a\right|<1. \end{array}\right. \end{array}$$
  *That is, $\lim_{n⟶\infty }\Sigma_{n}^{o}=\Sigma^{\infty }=0,\forall \Sigma_{1}^{o}\geq 0$, is the unique and stabilizing solution $\Sigma^{\infty }\geq 0$ of (241), i.e., such that $|M^{CL}\left(\Sigma^{\infty }\right)|<1$, if and only if |a|<1, and*
*$\lim_{n⟶\infty }\Sigma_{n}^{0}=\Sigma^{\infty }=\frac{K_{W}\left(a^{2}-1\right)}{{\left(c-a\right)}^{2}}>0,\forall \Sigma_{1}^{o}>0$ is the maximal and stabilizing solution $\Sigma^{\infty }$ of (241), i.e., such that $|M^{CL}\left(\Sigma^{\infty }\right)|<1$, if and only if |a|>1.*
*(5) Suppose c∈(−∞,∞) and |a|<1. Then, any solution $\Sigma_{t}^{o},t=1,2,\ldots ,n$ to the generalized DRE (239) with an arbitrary initial condition, $\Sigma_{1}^{o}\geq 0$, is such that $\lim_{n⟶\infty }\Sigma_{n}^{o}=\Sigma^{\infty }$, where $\Sigma^{\infty }\geq 0$ is the unique solution of the generalized ARE (241) with $M^{CL}\left(\Sigma^{\infty }\right)\in \left(-1,1\right)$, i.e., it is stabilizing. Moreover, $\Sigma^{\infty }=0$.*
*(6) (i) Suppose c∈(−∞,∞) and |a|<1. The entropy rate of $V_{t},\forall t\in Z_{+}$ is given by*
(244) $$\begin{array}{cc} H_{R}\left(V^{\infty }\right)= & \lim_{n⟶\infty }\frac{1}{n}\sum_{t=1}^{n}\frac{1}{2}\log\left(2\pi e\left[{\left(c-a\right)}^{2}\Sigma_{t}^{o}+K_{W}\right]\right) \end{array}$$
(245) $$\begin{array}{cc} = & \frac{1}{2}\log\left(2\pi eK_{W}\right),\;\forall \Sigma_{1}^{o}\geq 0. \end{array}$$
  *(ii) Suppose c∈(−∞,∞) and |a|>1. The entropy rate of $V_{t},\forall t\in Z_{+}$ is given by*
(246) $$\begin{array}{cc} H_{R}\left(V^{\infty }\right)= & \lim_{n⟶\infty }\frac{1}{n}\sum_{t=1}^{n}\frac{1}{2}\log\left(2\pi e\left[{\left(c-a\right)}^{2}\Sigma_{t}^{o}+K_{W}\right]\right) \end{array}$$
(247) $$\begin{array}{cc} = & \frac{1}{2}\log\left(2\pi eK_{W}a^{2}\right),\;\forall \Sigma_{1}^{o}>0. \end{array}$$

**Proof.**  See Appendix A.7.  □

**Remark 16.**  *Lemma 4(4) emphasizes the fact that in the asymptotic analysis of $\left\{\Sigma_{t}^{o},t=1,2,\ldots ,\right\}$, which satisfies the DREs (239) and (240), its limiting value, $\lim_{n⟶\infty }\Sigma_{n}^{o}=\Sigma^{\infty }$, where $\Sigma^{\infty }\geq 0$ satisfies the ARE (241), with two solutions $\Sigma^{\infty }=0$ and $\Sigma^{\infty }=\frac{K_{W}\left(a^{2}-1\right)}{{\left(c-a\right)}^{2}}$. For any c∈(−∞,∞), it is clear that for |a|<1, the unique and stabilizing solution is $\Sigma^{\infty }=0$, $\forall \Sigma_{1}^{o}\geq 0$, since the other solution $\Sigma^{\infty }=\frac{K_{W}\left(a^{2}-1\right)}{{\left(c-a\right)}^{2}}<0$ is negative. On the other hand, for any c∈(−∞,∞) and |a|>1, the stabilizing solution is the maximal solution, $\Sigma^{\infty }=\frac{K_{W}\left(a^{2}-1\right)}{{\left(c-a\right)}^{2}}>0$, provided $\Sigma_{1}^{o}>0$.*

To gain additional insights, we discuss the application of Lemma 4 to the AR(c),c∈(−∞,∞) noise in the next remark.

**Remark 17.**  *Entropy rate $H_{R}\left(V^{\infty }\right)$ of the AR(c),c∈(−∞,∞) noise*

*Consider the non-stationary AR(c),c∈(−∞,∞) noise defined by (124). Then, from Lemma 4, $\Sigma_{t}^{o},t=1,\ldots ,n$ is the solution of (239) and (240), with a=0 (see Corollary 6(b), (201)), and (241) degenerates to the ARE, as follows:*
(248) $$\begin{array}{c} \Sigma^{\infty }={\left(c\right)}^{2}\Sigma^{\infty }+K_{W}-{\left({\left(c\right)}^{2}\Sigma^{\infty }+K_{W}\right)}^{2}{\left(K_{W}+{\left(c\right)}^{2}\Sigma^{\infty }\right)}^{-1} \end{array}$$
  For a=0, by (242), the pair {A,C}={c,c} is detectable, and the pair $\left\{A^{*},GB^{*,\frac{1}{2}}\right\}=\left\{0,0\right\}$ is stabilizable. The two solutions of the ARE (248), without imposing $\Sigma^{\infty }\geq 0$, are
(249) $$\begin{array}{c} \Sigma^{\infty }=\left\{\begin{array}{cc} 0 & the\;unique,\;stabilizing,\;non\text{-}negative\;solution\;of\;the\;ARE\\ -\frac{K_{W}}{c^{2}}<0 & the\;non\text{-}stabilizing,\;negative\;solution\;of\;the\;ARE. \end{array}\right. \end{array}$$
  *That is, $\lim_{n⟶\infty }\Sigma_{n}^{o}=\Sigma^{\infty }\geq 0$, where $\Sigma^{\infty }=0$ is the unique (stabilizing) solution of the ARE and corresponds to the stable eigenvalue of the error equation (see (93), i.e., $M^{CL}\left(\Sigma^{\infty }\right)=c-\frac{K_{W}}{K_{W}}c=0$.*

Next, we compute the entropy rate $H_{R}\left(V^{\infty }\right)$ of the time-invariant non-stationary PO-SS$\left(a,c,b^{1},b^{2},d^{1},d^{2}\right)$ noise of Corollary 2 to show fundamental differences from the entropy rate $H_{R}\left(V^{\infty }\right)$ of the AR(a,c) noise of Lemma 4.

**Lemma 5.**  *Properties of solutions of DREs and AREs of PO-SS$\left(a,c,b^{1}=b,b^{2}=0,d^{1}=0,d^{2}=d\right)$ noise and entropy rate $H_{R}\left(V^{\infty }\right)$*

*Consider the the time-invariant non-stationary PO-SS$\left(a,c,b^{1},b^{2}=0,d^{1}=0,d^{2}=d\right)$ noise of Example 1, i.e., given by*
(250) $$\begin{array}{cc} \; & S_{t+1}=aS_{t}+bW_{t}^{1},\;t=1,2,\ldots ,n-1 \end{array}$$
(251) $$\begin{array}{cc} \; & V_{t}=cS_{t}+dW_{t}^{2},\;t=1,\ldots ,n, \end{array}$$
  *and the sequence $\Sigma_{t}^{o}\overset{▵}{=}\Sigma_{t},t=1,\ldots ,n$, generated by the DRE of Lemma 1 (see (113), i.e.,*
(252) $$\begin{array}{cc} \; & \Sigma_{t+1}^{o}={\left(a\right)}^{2}\Sigma_{t}^{o}+{\left(b\right)}^{2}K_{W^{1}}\\ \; & -{\left(a\Sigma_{t}^{o}c\right)}^{2}{\left({\left(d\right)}^{2}K_{W^{2}}+{\left(c\right)}^{2}\Sigma_{t}^{o}\right)}^{-1},\;t=1,\ldots ,n,\;\Sigma_{1}^{o}=K_{S_{1}}\geq 0,\;\Sigma_{t}^{o}\geq 0 \end{array}$$
  *where ${\left(b\right)}^{2}K_{W^{1}}\geq 0,{\left(d\right)}^{2}K_{W}^{2}>0$. Let $\Sigma^{\infty }\geq 0$ be the corresponding solution of generalized ARE:*
(253) $$\begin{array}{c} \Sigma^{\infty }={\left(a\right)}^{2}\Sigma^{\infty }+{\left(b\right)}^{2}K_{W^{1}}-{\left(a\Sigma^{\infty }c\right)}^{2}{\left({\left(d\right)}^{2}K_{W^{2}}+{\left(c\right)}^{2}\Sigma^{\infty }\right)}^{-1}. \end{array}$$
  *Then, the detectability and stabilizability pairs are*
(254) $$\begin{array}{c} \left\{A,C\right\}=\left\{a,c\right\},\;\left\{A^{*},GB^{*,\frac{1}{2}}\right\}=\left\{a,b{\left(K_{W^{1}}\right)}^{\frac{1}{2}}\right\}. \end{array}$$
  *and the following hold:*
*(1) The pair {A,C}={a,c} is detectable ∀c∈(−∞,∞),a∈(−∞,∞),c≠0. If c=0 the pair {A,C}={a,0} is detectable if and only if |a|<1.*
(2) The pair $\left\{A^{*},GB^{*,\frac{1}{2}}\right\}=\left\{a,b{\left(K_{W^{1}}\right)}^{\frac{1}{2}}\right\}$ is unit circle controllable if and only if $|b{\left(K_{W^{1}}\right)}^{\frac{1}{2}}|\neq 1$, ∀a∈(−∞,∞),c∈(−∞,∞).
(3) The pair $\left\{A^{*},GB^{*,\frac{1}{2}}\right\}=\left\{a,b{\left(K_{W^{1}}\right)}^{\frac{1}{2}}\right\}$ is stabilizable if $b{\left(K_{W^{1}}\right)}^{\frac{1}{2}}\neq 0$, ∀a∈(∞,∞),c∈(−∞,∞). If $b{\left(K_{W^{1}}\right)}^{\frac{1}{2}}=0$ the pair $\left\{A^{*},GB^{*,\frac{1}{2}}\right\}=\left\{a,0\right\}$ is stabilizable if and only if |a|<1.
*(4) Define the set*
(255) $$\begin{array}{cc} L^{\infty }\overset{▵}{=}\{\left(a,c,{\left(b\right)}^{2}K_{W^{1}}\right)\in & {\left(-\infty ,\infty \right)}^{2}\times \left[0,\infty \right):\;(i)\;the\;pair\;\{A,C\}=\{a,c\}\;is\;detectable,\;and\\ \; & (ii)\;the\;pair\;\left\{A^{*},GB^{*,\frac{1}{2}}\right\}=\left\{a,b{\left(K_{W^{1}}\right)}^{\frac{1}{2}}\right\}\;is\;stabilizable\}. \end{array}$$
  *For any $\left(a,c,b{\left(K_{W^{1}}\right)}^{\frac{1}{2}}\right)\in L^{\infty }$, any solution $\Sigma_{t}^{o},t=1,2,\ldots ,n$ to the (classical) DRE (252) with an arbitrary initial condition, $\Sigma_{1}^{o}\geq 0$ is such that $\lim_{n⟶\infty }\Sigma_{n}^{o}=\Sigma^{\infty }$, where $\Sigma^{\infty }\geq 0$ is the unique solution of the (classical) ARE (253) with $M^{CL}\left(\Sigma^{\infty }\right)\in \left(-1,1\right)$, i.e., it is stabilizing.*
*(5) For any $\left(a,c,b^{2}K_{W^{1}}\right)\in L^{\infty }$ of part (4), the entropy rate of $V_{t},\forall t\in Z_{+}$ is given by*
(256) $$\begin{array}{cc} H_{R}\left(V^{\infty }\right)= & \lim_{n⟶\infty }\frac{1}{n}\sum_{t=1}^{n}\frac{1}{2}\log\left(2\pi e\left[{\left(c\right)}^{2}\Sigma_{t}^{o}+{\left(d\right)}^{2}K_{W^{2}}\right]\right) \end{array}$$
(257) $$\begin{array}{cc} = & \frac{1}{2}\log\left(2\pi e\left[{\left(c\right)}^{2}\Sigma^{\infty }+{\left(d\right)}^{2}K_{W^{2}}\right]\right),\;\forall \Sigma_{1}^{o}\geq 0. \end{array}$$

**Proof.**  Follows from Theorem 5.  □

Next, we turn our attention to the convergence properties of the entropy rate $H_{R}\left(Y^{\infty }\right)$, which is needed for the characterization of $C^{fb,o}\left(\kappa \right)$ of Definition 4.

**Theorem 6.**  *Asymptotic properties of entropy rate $H_{R}\left(Y^{\infty }\right)$ of Theorem 3*

*Let $K_{t}^{o},t=1,\ldots ,$ be the solution of the generalized DRE (180) of the generalized Kalman filter of Theorem 3, corresponding to the time-invariant PO-SS realization of $V^{n}$ of Definition 2, $\left(A_{t},B_{t},C_{t},N_{t},K_{W_{t}}\right)=\left(A,B,C,N,K_{W}\right),\forall t$, with time-invariant strategies $\left(\Lambda_{t},K_{Z_{t}}\right)=\left(\Lambda^{\infty },K_{Z}^{\infty }\right)$, ∀t, generated by*
(258) $$\begin{array}{cc} \; & K_{t+1}^{o}=AK_{t}^{o}A^{T}+M\left(\Sigma_{t}^{o}\right)K_{{\hat{I}}_{t}^{o}}{\left(M(\Sigma_{t}^{o})\right)}^{T}\\ \; & -\left(AK_{t}^{o}{\left(\Lambda^{\infty }+C\right)}^{T}+M\left(\Sigma_{t}^{o}\right)K_{{\hat{I}}_{t}^{o}}\right){\left(K_{{\hat{I}}_{t}^{o}}+K_{Z}^{\infty }+\left(\Lambda^{\infty }+C\right)K_{t}^{o}{\left(\Lambda^{\infty }+C\right)}^{T}\right)}^{-1}\\ \; & .{\left(AK_{t}^{o}{\left(\Lambda^{\infty }+C\right)}^{T}+M\left(\Sigma_{t}^{o}\right)K_{{\hat{I}}_{t}^{o}}\right)}^{T},\;K_{t}^{o}=K_{t}^{o,T}⪰0,\;t=1,\ldots ,n,\;K_{1}^{o}=0 \end{array}$$
  *where*
(259) $$\begin{array}{cc} \; & K_{{\hat{I}}_{t}^{o}}=C\Sigma_{t}^{o}C^{T}+NK_{W}N^{T},\;\Sigma_{t}^{o}\;is\;a\;solution\;of\;\text{(231)},\;M\left(\Sigma^{o}\right)\;is\;given\;by\;\text{(232)}, \end{array}$$
(260) $$\begin{array}{cc} \; & F^{CL}\left(\Sigma^{o},K^{o}\right)\overset{▵}{=}A-F\left(\Sigma^{o},K^{o}\right)\left(\Lambda^{\infty }+C\right), \end{array}$$
(261) $$\begin{array}{cc} \; & F\left(\Sigma^{o},K^{o}\right)\overset{▵}{=}\left(AK^{o}{\left(\Lambda^{\infty }+C\right)}^{T}+M\left(\Sigma^{o}\right)K_{{\hat{I}}^{o}}\right){\left\{K_{{\hat{I}}^{o}}+K_{Z^{\infty }}+\left(\Lambda^{\infty }+C\right)K^{o}{\left(\Lambda^{\infty }+C\right)}^{T}\right\}}^{-1}. \end{array}$$
  *Define the corresponding generalized ARE by*
(262) $$\begin{array}{cc} \; & K^{\infty }=AK^{\infty }A^{T}+M\left(\Sigma^{\infty }\right)K_{{\hat{I}}^{\infty }}{\left(M(\Sigma^{\infty })\right)}^{T}-\left(AK^{\infty }{\left(\Lambda^{\infty }+C\right)}^{T}+M\left(\Sigma^{\infty }\right)K_{{\hat{I}}^{\infty }}\right)(K_{{\hat{I}}^{\infty }}+K_{Z}^{\infty }\\ \; & +\left(\Lambda^{\infty }+C\right)K^{\infty }{\left(\Lambda^{\infty }+C\right)}^{T}{)}^{-1}{\left(AK^{\infty }{\left(\Lambda^{\infty }+C\right)}^{T}+M\left(\Sigma^{\infty }\right)K_{{\hat{I}}^{\infty }}\right)}^{T},\;K^{\infty }=K^{\infty ,T}⪰0. \end{array}$$
  *where*
(263) $$\begin{array}{cc} \; & K_{{\hat{I}}^{\infty }}=C\Sigma^{\infty }C^{T}+NK_{W}N^{T},\;\Sigma^{\infty }\;is\;a\;solution\;of\;\text{(233)},\;M\left(\Sigma^{\infty }\right)\;is\;given\;by\;\text{(232)}. \end{array}$$
  *Introduce the matrices*
(264) $$\begin{array}{cc} \; & C\left(\Lambda^{\infty }\right)\overset{▵}{=}\Lambda^{\infty }+C,\;GQG^{T}\overset{▵}{=}M\left(\Sigma^{\infty }\right)K_{{\hat{I}}^{\infty }}{\left(M(\Sigma^{\infty })\right)}^{T}, \end{array}$$
(265) $$\begin{array}{cc} \; & GS\overset{▵}{=}M\left(\Sigma^{\infty }\right)K_{{\hat{I}}^{\infty }},\;R\left(K_{Z}^{\infty }\right)\overset{▵}{=}K_{{\hat{I}}^{\infty }}+K_{Z}^{\infty }. \end{array}$$
(266) $$\begin{array}{cc} \; & ⟹\;\;G\overset{▵}{=}M\left(\Sigma^{\infty }\right),\;Q\overset{▵}{=}K_{{\hat{I}}^{\infty }},\;S\overset{▵}{=}K_{{\hat{I}}^{\infty }}, \end{array}$$
(267) $$\begin{array}{cc} \; & A^{*}\left(\Lambda^{\infty },K_{Z}^{\infty }\right)\overset{▵}{=}A-M\left(\Sigma^{\infty }\right)K_{{\hat{I}}^{\infty }}{\left(K_{{\hat{I}}^{\infty }}+K_{Z}^{\infty }\right)}^{-1}\left(\Lambda^{\infty }+C\right), \end{array}$$
(268) $$\begin{array}{cc} \; & B^{*}\left(K_{Z}^{\infty }\right)\overset{▵}{=}K_{{\hat{I}}^{\infty }}-K_{{\hat{I}}^{\infty }}{\left(K_{{\hat{I}}^{\infty }}+K_{Z}^{\infty }\right)}^{-1}K_{{\hat{I}}^{\infty }}. \end{array}$$
  *Suppose that the detectability and stabilizability conditions of Corollary 8(i,ii) hold.*
*Then, all statements of Theorem 5 hold with $\left(C\left(\Lambda^{\infty }\right),G,Q,S,R\left(K_{Z}^{\infty }\right)\right)$ as defined by (265).*
*In particular, suppose*
*(i) $\left\{A,C\left(\Lambda^{\infty }\right)\right\}=\left\{A,\Lambda^{\infty }+C\right\}$ is detectable;*
(ii) $\left\{A^{*}\left(\Lambda^{\infty },K_{Z}^{\infty }\right),GB^{*,\frac{1}{2}}\left(K_{Z}^{\infty }\right)\right\}$ is stabilizable.
*Then, any solution $K_{t}^{o},t=1,2,\ldots ,n$ to the generalized matrix DRE (258) with an arbitrary initial condition $K_{1}^{o}⪰0$ is such that $\lim_{n⟶\infty }K_{n}^{o}=K^{\infty }$, where $K^{\infty }⪰0$ is the unique solution of the generalized matrix ARE (262) with $spec\left(F^{CL}\left(K^{\infty },\Sigma^{\infty }\right)\right)\in D_{o}$, i.e., it is stabilizing.*
*Moreover, the entropy rate of $Y^{n}$ is given by*
(269) $$\begin{array}{cc} \; & H_{R}\left(Y^{\infty }\right)=\lim_{n⟶\infty }\frac{1}{n}\sum_{t=1}^{n}H\left(I_{t}^{o}\right) \end{array}$$
(270) $$\begin{array}{cc} \; & =\lim_{n⟶\infty }\frac{1}{2n}\sum_{t=1}^{n}\log\left(2\pi e\left[\left(\Lambda^{\infty }+C\right)K_{t}^{o}{\left(\Lambda^{\infty }+C\right)}^{T}+K_{{\hat{I}}_{t}^{o}}+K_{Z}^{\infty }\right]\right) \end{array}$$
(271) $$\begin{array}{cc} \; & =H\left(I_{t}^{\infty }\right)\overset{▵}{=}\frac{1}{2}\log\left(2\pi e\left[\left(\Lambda^{\infty }+C\right)K^{\infty }{\left(\Lambda^{\infty }+C\right)}^{T}+K_{{\hat{I}}^{\infty }}+K_{Z}^{\infty }\right]\right),\;\forall K_{1}^{o}⪰0,\;\forall t \end{array}$$
  *where $I_{t}^{o},t=1,\ldots ,n$ is the innovations process of Theorem 3 (with indicated changes of time-invariant strategies) and where*
(272) $$\begin{array}{cc} \; & I_{t}^{\infty }=\left(\Lambda^{\infty }+C\right)\left({\hat{S}}_{t}^{\infty }-\hat{{\hat{S}}_{t}^{\infty }}\right)+{\hat{I}}_{t}^{\infty }+Z_{t}, \end{array}$$
(273) $$\begin{array}{cc} \; & I_{t}^{\infty }\in N\left(0,\left(\Lambda^{\infty }+C\right)K^{\infty }{\left(\Lambda^{\infty }+C\right)}^{T}+K_{{\hat{I}}^{\infty }}+K_{Z}^{\infty }\right),\;t=1,2,\ldots , \end{array}$$
  *is the stationary Gaussian innovations process, i.e., with $\left(K_{t}^{o},\Sigma_{t}^{o}\right)$ replaced by $\left(K^{\infty },\Sigma^{\infty }\right)$.*

**Proof.**  Since the detectability and stabilizability conditions of Corollary 8 hold, then the statements of Corollary 8 hold. By the continuity property of the solutions of generalized difference Riccati equations, with respect to its coefficients (see [16]), and the convergence of the sequence $\lim_{n⟶\infty }\Sigma_{n}^{\infty }=\Sigma^{\infty }$, where $\Sigma^{\infty }⪰0$ is the unique stabilizing solution of (233), then the statements of Theorem 6 hold, as stated. In particular, under the detectability and stabilizability Conditions (i) and (ii), $\lim_{n⟶\infty }K_{n}^{o}=K^{\infty }$, where $K^{\infty }⪰0$ is the unique and stabilizing solution of (262).  □

In the next lemma, we apply Theorem 6 to the AR(a,c),a∈(−∞,∞),c∈(−∞,∞) noise of Example 2(a) using Lemma 4.

**Lemma 6.**  *Consider the AR(a,c),a∈(−∞,∞),c∈(−∞,∞) noise of Example 2(a), and the DRE $\Sigma_{t}^{o}\overset{▵}{=}\Sigma_{t},t=1,\ldots ,n$ and ARE of Lemma 4, (239)–(242).*

*Let $K_{t}^{o},t=1,\ldots ,n$ denote the solution of the DRE of Corollary 6(a), when $\Lambda_{t}=\Lambda^{\infty },K_{Z_{t}}=K_{Z}^{\infty },K_{t}=K_{t}^{o},\forall t$, i.e., given by*
$$\begin{array}{cc} K_{t+1}^{o}= & {\left(c\right)}^{2}K_{t}^{o}+{\left(M(\Sigma_{t}^{o})\right)}^{2}K_{{\hat{I}}_{t}^{o}}-{\left(cK_{t}^{o}\left(\Lambda^{\infty }+c-a\right)+M\left(\Sigma_{t}^{o}\right)K_{{\hat{I}}_{t}^{o}}\right)}^{2} \end{array}$$
(274) $$\begin{array}{cc} \; & \;.{\left(K_{{\hat{I}}_{t}^{o}}+K_{Z}^{\infty }+{\left(\Lambda^{\infty }+c-a\right)}^{2}K_{t}^{o}\right)}^{-1},\;K_{1}^{o}=0,\;t=1,\ldots ,n, \end{array}$$
(275) $$\begin{array}{cc} K_{Z}^{\infty }\geq & 0,\;K_{t}^{o}\geq 0,\;t=1,\ldots ,n \end{array}$$
  *and where*
(276) $$\begin{array}{cc} \; & M\left(\Sigma_{t}^{o}\right)\overset{▵}{=}\left(c\Sigma_{t}^{o}\left(c-a\right)+K_{W}\right){\left(K_{W}+{\left(c-a\right)}^{2}\Sigma_{t}^{o}\right)}^{-1}, \end{array}$$
(277) $$\begin{array}{cc} \; & K_{{\hat{I}}_{t}^{o}}={\left(c-a\right)}^{2}\Sigma_{t}^{o}+K_{W},\;t=1,\ldots ,n. \end{array}$$
  *Define the set*
(278) $$\begin{array}{cc} L^{\infty }\overset{▵}{=}\{\left(a,c\right)\in {\left(-\infty ,\infty \right)}^{2}, & a\neq c:\;(i)\;the\;pair\;\{A,C\}=\{a,c-a\}\;is\;detectable,\;and\\ \; & (ii)\;the\;pair\;\left\{A^{*},GB^{*,\frac{1}{2}}\right\}=\left\{a,0\right\}\;is\;stabilizable\}. \end{array}$$
  *For any $\left(a,c\right)\in L^{\infty }$, let $K^{\infty }\geq 0$ be a corresponding solution of the ARE (evaluated at $\lim_{n⟶\infty }\Sigma_{n}^{\infty }=\Sigma^{\infty }=0$),*
(279) $$\begin{array}{cc} K^{\infty }= & {\left(c\right)}^{2}K^{\infty }+K_{W}-{\left(cK^{\infty }\left(\Lambda^{\infty }+c-a\right)+K_{W}\right)}^{2}{\left(K_{W}+K_{Z}^{\infty }+{\left(\Lambda^{\infty }+c-a\right)}^{2}K^{\infty }\right)}^{-1}. \end{array}$$
(280) $$\begin{array}{cc} K_{Z}^{\infty }\geq & 0,\;K_{W}>0. \end{array}$$
  *and define the pairs*
(281) $$\begin{array}{cc} \; & \{A,C\left(\Lambda^{\infty }\right\}=\left\{c,\Lambda^{\infty }+c-a\right\}, \end{array}$$
(282) $$\begin{array}{cc} \; & \left\{A^{*}\left(\Lambda^{\infty },K_{Z}^{\infty }\right),GB^{*,\frac{1}{2}}\left(K_{Z}^{\infty }\right)\right\}\\ \; & =\{c-K_{W}{\left(K_{W}+K_{Z}^{\infty }\right)}^{-1}\left(\Lambda^{\infty }+c-a\right),{\left(K_{W}-{\left(K_{W}\right)}^{2}{\left(K_{W}+K_{Z}^{\infty }\right)}^{-1}\right)}^{\frac{1}{2}}\}. \end{array}$$
  *Then, the following hold:*
*(1) Suppose $\Lambda^{\infty }+c-a\neq 0$. Then, $\{A,C\left(\Lambda^{\infty }\right\}=\left\{c,\Lambda^{\infty }+c-a\right\}$ is detectable $\forall \left(a,c\right)\in {\left(-\infty ,\infty \right)}^{2}$.*
*(2) Suppose $\Lambda^{\infty }+c-a=0$. Then, $\{A,C\left(\Lambda^{\infty }\right\}=\left\{c,0\right\}$ is detectable for if and only if |c|<1∀a∈(−∞,∞).*
(3) Suppose $K_{Z}^{\infty }=0$. Then, the pair $\left\{A^{*},GB^{*,\frac{1}{2}}\right\}=\left\{-\Lambda^{\infty }+a,0\right\}$ is unit circle controllable if and only if |Λ−a|≠1∀a∈(−∞,∞).
(4) Suppose $K_{Z}^{\infty }=0$. Then, the pair $\left\{A^{*},GB^{*,\frac{1}{2}}\right\}=\left\{-\Lambda^{\infty }+a,0\right\}$ is stabilizable if and only if |Λ−a|<1∀a∈(−∞,∞).
*(5) Suppose $\Lambda^{\infty }+c-a\neq 0,\left|\Lambda -a\right|\neq 1$$\forall \left(a,c\right)\in {\left(-\infty ,\infty \right)}^{2}$, and $K_{Z}^{\infty }=0$, $\Sigma_{1}^{o}=0$. The sequence $K_{t}^{o},t=1,2,\ldots ,n$ that satisfies the generalized DRE (274) with a zero initial condition, $K_{1}^{o}=0$, converges to $K^{\infty }\geq 0$, i.e., $\lim_{n⟶\infty }K_{n}^{o}=K^{\infty }$, where $K^{\infty }$ satisfies the generalized ARE,*
(283) $$\begin{array}{cc} K^{\infty }= & {\left(c\right)}^{2}K^{\infty }+K_{W}-{\left(cK^{\infty }\left(\Lambda^{\infty }+c-a\right)+K_{W}\right)}^{2}{\left(K_{W}+{\left(\Lambda^{\infty }+c-a\right)}^{2}K^{\infty }\right)}^{-1},\;K^{\infty }\geq 0 \end{array}$$
  if and only if |a|<1 (by Lemma 4(4)), and the pair $\left\{A^{*},GB^{*,\frac{1}{2}}\right\}=\left\{-\Lambda^{\infty }+a,0\right\}$ is stabilizable, equivalently, $|\Lambda^{\infty }-a|<1$.
*Moreover, the solutions of the ARE (283), under the stabilizability condition, i.e., $|\Lambda^{\infty }-a|<1$, are*
(284) $$\begin{array}{c} K^{\infty }=\left\{\begin{array}{cc} 0 & the\;unique,\;stabilizing,\;K^{\infty }\geq 0\;sol.\;of\;\text{(283)}\;for\;\left|\Lambda^{\infty }-a\right|<1\\ \frac{K_{W}\left({\left(\Lambda^{\infty }-a\right)}^{2}-1\right)}{{\left(\Lambda^{\infty }+c-a\right)}^{2}}<0 & the\;non\text{-}stabilizing,\;K^{\infty }<0\;sol.\;of\;\text{(283)}\;for\;\left|\Lambda^{\infty }-a\right|<1. \end{array}\right. \end{array}$$
  *That is, $\lim_{n⟶\infty }\Sigma_{n}^{o}=\Sigma^{\infty }=0$ is the unique and stabilizing solution $\Sigma^{\infty }\geq 0$ of (283), i.e., such that $|M^{CL}\left(\Sigma^{\infty }\right)|<1$, if and only if $|\Lambda^{\infty }-a|<1,|a|<1$.*

**Proof.**  The statements follow from Lemma 4, Theorem 6 (and general properties of Theorem 5).  □

**Remark 18.**  *From Lemma 6(5), it follows that if $K_{Z}^{\infty }=0,\Sigma_{1}^{o}=0$, then the unique and stabilizing solution is $K^{\infty }=0$ and corresponds to $|\Lambda^{\infty }-a|<1,a\in \left(-1,1\right)$. This is an application of Theorem 5(1).*

In the next theorem, we characterize the asymptotic limit of Definition 4 by invoking Theorems 3 and 6 and Corollary 8.

**Theorem 7.**  *Feedback capacity $C^{fb,o}\left(\kappa \right)$ of Theorem 3 for time-invariant strategies*

*Consider $C^{fb,o}\left(\kappa \right)$ of Definition 4 corresponding to Theorem 3, i.e., the PO-SS realization of $V^{n}$ of Definition 2 is time-invariant, $\left(A_{t},B_{t},C_{t},N_{t},K_{W_{t}}\right)=\left(A,B,C,N,K_{W}\right),\forall t$, and the strategies are time-invariant, $\left(\Lambda_{t},K_{Z_{t}}\right)=\left(\Lambda^{\infty },K_{Z}^{\infty }\right),\forall t$.*
*Define the set*
(285) $$\begin{array}{cc} P^{\infty }\overset{▵}{=} & \{\left(\Lambda^{\infty },K_{Z}^{\infty }\right)\in \left(-\infty ,\infty \right)\times \left[0,\infty \right):\\ \; & (i)\{A,C\}\;of\;Corollary\;8\;is\;detectable;\\ \; & \left(ii\right)\left\{A^{*},GB^{*,\frac{1}{2}}\right\}\;of\;Corollary\;8\;is\;stabilizable,\;where\;\left(A^{*},B^{*}\right)\;is\;defined\;by\;\text{(235)};\\ \; & \left(iii\right)\left\{A,C\left(\Lambda^{\infty }\right)\right\}=\left\{A,\Lambda^{\infty }+C\right\}\;of\;Theorem\;6\;is\;detectable;\\ \; & \left(iv\right)\{A^{*}\left(\Lambda^{\infty },K_{Z}^{\infty }\right),GB^{*,\frac{1}{2}}\left(K_{Z}^{\infty }\right)\}\;of\;Theorem\;6\;is\;stabilizable;\\ \; & \left(A^{*}\left(\Lambda^{\infty },K_{Z}^{\infty }\right),B^{*}\left(K_{Z}^{\infty }\right)\right)is\;defined\;by\;\text{(268)}.\}. \end{array}$$
  *Then,*
$$\begin{array}{cc} \; & C^{fb,o}\left(\kappa \right)\overset{▵}{=}\underset{\left(\Lambda^{\infty },K_{Z}^{\infty }\right)\in P^{\infty }:\;\lim_{n⟶\infty }\frac{1}{n}\sum_{t=1}^{n}\left(\Lambda^{\infty }K_{t}^{o}{\left(\Lambda^{\infty }\right)}^{T}+K_{Z}^{\infty }\right)\leq \kappa }{\sup}\{ \end{array}$$
(286) $$\begin{array}{cc} \; & \lim_{n⟶\infty }\frac{1}{2n}\sum_{t=1}^{n}\log\left(\frac{\left(\Lambda^{\infty }+C\right)K_{t}^{o}{\left(\Lambda^{\infty }+C\right)}^{T}+C\Sigma_{t}^{o}C^{T}+NK_{W}N^{T}+K_{Z}^{\infty }}{C\Sigma_{t}^{o}C^{T}+NK_{W}N^{T}}\right)\} \end{array}$$
(287) $$\begin{array}{cc} = & \underset{\left(\Lambda^{\infty },K_{Z}^{\infty }\right)\in P^{\infty }\left(\kappa \right)}{\sup}\frac{1}{2}\log\left(\frac{\left(\Lambda^{\infty }+C\right)K^{\infty }{\left(\Lambda^{\infty }+C\right)}^{T}+C\Sigma^{\infty }C^{T}+NK_{W}N^{T}+K_{Z}^{\infty }}{C\Sigma^{\infty }C^{T}+NK_{W}N^{T}}\right) \end{array}$$
  *where*
(288) $$\begin{array}{cc} P^{\infty }\left(\kappa \right)\overset{▵}{=} & \{\left(\Lambda^{\infty },K_{Z}^{\infty }\right)\in P^{\infty }:K_{Z}^{\infty }\geq 0,\;\Lambda^{\infty }K^{\infty }{\left(\Lambda^{\infty }\right)}^{T}+K_{Z}^{\infty }\leq \kappa ,\\ \; & K^{\infty }\;is\;the\;unique\;and\;stabilizing\;solution\;of\;\text{(262)},\;i.e.,\;|F^{CL}\left(\Sigma^{\infty },K^{\infty }\right)|<1\\ \; & \Sigma^{\infty }\;is\;the\;unique,\;stabilizing\;solution\;of\;\text{(233)},\;i.e.,\;|M^{CL}\left(\Sigma^{\infty }\right)|<1,\} \end{array}$$
  *provided there exists κ∈[0,∞) such that the set $P^{\infty }\left(\kappa \right)$ is non-empty.*
*Moreover, the maximum element $\left(\Lambda^{\infty },K_{Z}^{\infty }\right)\in P^{\infty }\left(\kappa \right)$ is such that*
*(1) It induces asymptotic stationarity of the corresponding input and innovations processes (see Theorem 3 for specification);*
*(2) If $V^{n},n=1,2,\ldots$ is asymptotically stationary, then it induces asymptotic stationarity of the corresponding, input and output processes;*
*(3) For (i) and (ii), $C^{fb,o}\left(\kappa \right)$ is independent of the initial conditions $K_{1}^{o}⪰0,\Sigma_{1}^{o}⪰0$.*
Furthermore, if the set $P^{\infty }\left(\kappa \right)$ is empty, replace stabilizability of $\left\{A^{*},GB^{*,\frac{1}{2}}\right\}$ and $\left\{A^{*}\left(\Lambda^{\infty },K_{Z}^{\infty }\right),GB^{*,\frac{1}{2}}\left(K_{Z}^{\infty }\right)\right\}$ by unit circle controllablilty, i.e., the maximal and stabilizing solutions $K^{\infty },\Sigma^{\infty }$ of the AREs are utilized.

**Proof.**  By Definition 4, Theorems 3 and 6 and Corollary 8, (286) follows. We defined the set $P^{\infty }$ using the detectability and stabilizability conditions of Corollary 8 and Theorem 6 to ensure the convergence of solutions $\left\{\left(K_{t}^{o},\Sigma_{t}^{o}\right):t=1,2,\ldots ,n\right\}$ of the generalized matrix DREs to unique non-negative, stabilizing solutions of the corresponding generalized matrix AREs. Then, for any element $\left(\Lambda^{\infty },K_{Z}^{\infty }\right)\in P^{\infty }$, both summands in (286) converge. This establishes the characterization of the right-hand side of (287). Parts (1)–(3) follow from the asymptotic properties of the Kalman filter (due to the stabilizability and detectability conditions). The last statement follows due to the relaxation, Theorem 5(4).  □

**Remark 19.**  *In Theorem 7, if we replace stabilizability by unit circle controllability, then the supremum in (288) is over a larger set. However, the asymptotic limits $\left(\Sigma^{\infty },K^{\infty }\right)$ are not unique stabilizing solutions but are instead the maximal and stabilizing solutions.*

Theorem 7 also holds for asymptotically time-invariant strategies. We state this as a corollary.

**Corollary 9.**  *Feedback capacity $C^{fb,o}\left(\kappa \right)$ of Theorem 3 for asymptotically time-invariant strategies*

*Consider the problem statement of Theorem 7 with asymptotically time-invariant strategies, $\lim_{n⟶\infty }\left(\Lambda_{n},K_{Z_{n}}\right)=\left(\Lambda^{\infty },K_{Z}^{\infty }\right)$ and corresponding $K_{n},n=1,2,\ldots$.*
*Then,*
$$\begin{array}{cc} \; & C^{fb,o}\left(\kappa \right)\overset{▵}{=}\underset{\lim_{n⟶\infty }\left(\Lambda_{n},K_{Z_{n}}\right)=\left(\Lambda^{\infty },K_{Z}^{\infty }\right)\in P^{\infty }:\;\lim_{n⟶\infty }\frac{1}{n}\sum_{t=1}^{n}\left(\Lambda_{t}K_{t}{\left(\Lambda_{t}\right)}^{T}+K_{Z_{t}}\right)\leq \kappa }{\sup}\{ \end{array}$$
(289) $$\begin{array}{cc} \; & \lim_{n⟶\infty }\frac{1}{2n}\sum_{t=1}^{n}\log\left(\frac{\left(\Lambda_{t}+C\right)K_{t}{\left(\Lambda_{t}+C\right)}^{T}+C\Sigma_{t}C^{T}+NK_{W}N^{T}+K_{Z_{t}}}{C\Sigma_{t}C^{T}+NK_{W}N^{T}}\right)\} \end{array}$$
(290) $$\begin{array}{cc} = & \text{(287)} \end{array}$$
  *and the statements of Theorem 7 hold.*

**Proof.**  This follows from Proposition 4 and Theorem 7.  □

**Remark 20.**  *Explicit closed-form expressions of $C^{fb,o}\left(\kappa \right)$ are given in [1,2] for the stable and unstable AR and ARMA noise processes $V^{n}$. The expressions $C^{fb,o}\left(\kappa \right)$ consist of multiple regimes that depend on the parameters of noise, i.e., $\left(c,a,K_{W}\right)$ for the ARMA noise and value of κ. Moreover, for some regimes, $C^{fb,o}\left(\kappa \right)$ is achieved by an optimal $K_{Z}^{\infty }>0$, while for other regimes, it is achieved by $\lim_{n⟶\infty }K_{Z_{n}}=K_{Z}^{\infty }=0$, such that $K_{Z_{1}}>0,K_{Z_{t}}=0,t=2,3,\ldots ,n$.*

Next, we give the expression of feedback capacity $C^{fb}\left(\kappa \right)$, which is generally an upper bound on the expressions of Theorem 7 and Corollary 9.

**Theorem 8.**  *Feedback capacity $C^{fb}\left(\kappa \right)$ of Theorem 3 for asymptotically time-invariant noise and strategies*

*Consider $C^{fb}\left(\kappa \right)$ of Definition 4 corresponding to Theorem 3, where $(\Lambda_{t},K_{Z_{t}}),t=1,\ldots ,n$ and the coefficients of the PO-SS realization of $V^{n}$ of Definition 2 are asymptotically time-invariant, i.e.,*
(291) $$\begin{array}{c} \left(a\right)\;\lim_{n⟶\infty }\left(\Lambda_{n},K_{Z_{n}}\right)=\left(\Lambda^{\infty },K_{Z}^{\infty }\right);\;\left(b\right)\lim_{n⟶\infty }\left(A_{n},B_{n},C_{n},N_{n},K_{W_{n}}\right)=\left(A,B,C,N,K_{W}\right). \end{array}$$
  Let $P^{\infty ,ucc}$ correspond to $P^{\infty }=$ (285) with $\left\{A^{*},GB^{*,\frac{1}{2}}\right\}$ and $\left\{A^{*}\left(\Lambda^{\infty },K_{Z}^{\infty }\right),GB^{*,\frac{1}{2}}\left(K_{Z}^{\infty }\right)\right\}$ being replaced by unit circle controllablity.
*Let $P^{\infty ,ucc}\left(\kappa \right)$ correspond to $P^{\infty }\left(\kappa \right)=$ (288), with $K^{\infty }$ and $\Sigma^{\infty }$ being the maximal and stabilizing solution of (262) and the maximal and stabilizing solution of (233), respectively.*
*Then,*
$$\begin{array}{cc} \; & C^{fb}\left(\kappa \right)\overset{▵}{=}\lim_{n⟶\infty }\underset{{\left\{\Lambda_{t},K_{Z_{t}}\right\}}_{t=1}^{n}:\lim_{n⟶\infty }\left(\Lambda_{n},K_{Z_{n}}\right)=\left(\Lambda^{\infty },K_{Z}^{\infty }\right)\in P^{\infty ,ucc},\frac{1}{n}\sum_{t=1}^{n}\left(\Lambda_{t}K_{t}{\left(\Lambda_{t}\right)}^{T}+K_{Z_{t}}\right)\leq \kappa }{\sup}\{ \end{array}$$
(292) $$\begin{array}{cc} \; & \frac{1}{2n}\sum_{t=1}^{n}\log\left(\frac{\left(\Lambda_{t}+C_{t}\right)K_{t}{\left(\Lambda_{t}+C_{t}\right)}^{T}+C_{t}\Sigma_{t}C_{t}^{T}+N_{t}K_{W_{t}}N_{t}^{T}+K_{Z_{t}}}{C_{t}\Sigma_{t}C_{t}^{T}+N_{t}K_{W_{t}}N_{t}^{T}}\right)\} \end{array}$$
(293) $$\begin{array}{cc} = & \underset{\left(\Lambda^{\infty },K_{Z}^{\infty }\right)\in P^{\infty ,ucc}\left(\kappa \right)}{\sup}\frac{1}{2}\log\left(\frac{\left(\Lambda^{\infty }+C\right)K^{\infty }{\left(\Lambda^{\infty }+C\right)}^{T}+C\Sigma^{\infty }C^{T}+NK_{W}N^{T}+K_{Z}^{\infty }}{C\Sigma^{\infty }C^{T}+NK_{W}N^{T}}\right) \end{array}$$
(294) $$\begin{array}{cc} \geq & C^{fb,o}\left(\kappa \right). \end{array}$$

**Proof.**  First, we note that Theorem 7 continuous to hold, if we consider asymptotically time-invariant strategies and coefficients, i.e., (a) and (b) (by Proposition 4), and the stabilizability conditions are replaced by unit circle controllability conditions. Hence, (288) remains valid with the set $P^{\infty }\left(\kappa \right)$ replaced by the larger set $P^{\infty ,ucc}\left(\kappa \right)$, giving the higher value (293) ≥ (288). For the derivation of (292) = (293), it suffices to show we can interchange the $\lim_{n⟶\infty }$ and the supremum in (292). This can be completed by using the definition of the set $P^{\infty ,ucc}\left(\kappa \right)$ and Conditions (a) and (b). The procedure, although lengthy, is similar to the one described in [41]; hence, we omit it.  □

**Conclusion 1.**  *Degenerate versions of Theorem 7, Theorem 8 for feedback code of Definition 3, i.e., $\left(s,2^{nR},n\right)$, n=1,2,…*

*The characterizations of feedback capacity $C^{fb,o}\left(\kappa ,s\right)$ of the AGN channel (1) driven by a noise $V^{n}$ of Definition 2, for the code of Definition 3, i.e., $\left(s,2^{nR},n\right)$, n=1,2,…, are degenerate cases of Theorem 7 and Theorem 8, corresponding to $\Sigma_{t}=\Sigma_{t}^{s},t=1,\ldots ,\Sigma_{1}=\Sigma_{1}^{s}=0$. In particular, since Theorem 7 characterizes $C^{fb,o}\left(\kappa \right)$ for all initial data $\Sigma_{1}⪰0$, then it includes $\Sigma_{1}=\Sigma_{1}^{s}=0$. Moreover, it follows that $C^{fb,o}\left(\kappa \right)=C^{fb,o}\left(\kappa ,s\right)$, where $C^{fb,o}\left(\kappa ,s\right)$ is independent of the initial state $S_{1}^{s}=s$.*

We apply Theorem 7 to obtain $C^{fb,o}\left(\kappa \right)$ of the AR(a,c),a∈(−∞,∞),c∈(−∞,∞) noise.

**Corollary 10.**  *Consider the AR(a,c),a∈(−∞,∞),c∈(−∞,∞) noise of Example 2(a).*

*Define the set*
(295) $$\begin{array}{cc} P^{\infty }\overset{▵}{=} & \{\left(\Lambda^{\infty },K_{Z}^{\infty }\right)\in \left(-\infty ,\infty \right)\times \left[0,\infty \right):\\ \; & (i)c\in (-\infty ,\infty ),a\in (-1,1),c\neq a,\\ \; & \left(ii\right)\;the\;pair\;\left\{A,C\left(\Lambda^{\infty }\right)\right\}\overset{▵}{=}\left\{c,\Lambda^{\infty }+c-a\right\}\;is\;detectable,\\ \; & \left(ii\right)\;the\;pair\;\{A^{*}\left(\Lambda^{\infty },K_{Z}^{\infty }\right),GB^{*,\frac{1}{2}}\left(K_{Z}^{\infty }\right)\}\;is\;stabilizable,\;where\\ \; & A^{*}\left(\Lambda^{\infty },K_{Z}^{\infty }\right)\overset{▵}{=}\;c-K_{W}{\left(K_{W}+K_{Z}^{\infty }\right)}^{-1}\left(\Lambda^{\infty }+c-a\right),\\ \; & GB^{*,\frac{1}{2}}\left(K_{Z}^{\infty }\right)\overset{▵}{=}{\left(K_{W}-{\left(K_{W}\right)}^{2}{\left(K_{W}+K_{Z}^{\infty }\right)}^{-1}\right)}^{\frac{1}{2}}\}. \end{array}$$
  *Then,*
(296) $$\begin{array}{c} C^{fb,o}\left(\kappa \right)=\underset{\left(\Lambda^{\infty },K_{Z}^{\infty }\right)\in P^{\infty }\left(\kappa \right)}{\sup}\frac{1}{2}\log\left(\frac{{\left(\Lambda^{\infty }+c-a\right)}^{2}K^{\infty }+K_{W}+K_{Z}^{\infty }}{K_{W}}\right)=C^{fb,o}\left(\kappa ,s\right),\;\forall s \end{array}$$
  *where*
(297) $$\begin{array}{cc} P^{\infty } & \left(\kappa \right)\overset{▵}{=}\{\left(\Lambda^{\infty },K_{Z}^{\infty }\right)\in P^{\infty }:\;{\left(\Lambda^{\infty }\right)}^{2}K^{\infty }+K_{Z}^{\infty }\leq \kappa ,\\ \; & K^{\infty }\geq 0\;is\;the\;unique\;and\;stabilizing\;solution\;of\\ \; & K^{\infty }={\left(c\right)}^{2}K^{\infty }+K_{W}\\ \; & -{\left(cK^{\infty }\left(\Lambda^{\infty }+c-a\right)+K_{W}\right)}^{2}{\left(K_{W}+K_{Z}^{\infty }+{\left(\Lambda^{\infty }+c-a\right)}^{2}K^{\infty }\right)}^{-1}\} \end{array}$$
  *provided that there exists κ∈[0,∞) such that the set $P^{\infty }\left(\kappa \right)$ is non-empty.*
*Moreover, the maximum element $\left(\Lambda^{\infty },K_{Z}^{\infty }\right)\in P^{\infty }\left(\kappa \right)$, is such that,*
*(1) It induces asymptotic stationarity of the corresponding, input and innovations processes;*
*(2) If $V^{n},n=1,2,\ldots$ is asymptotically stationary, then it induces asymptotic stationarity of the corresponding, input and output processes;*
*(3) For (i) and (ii), $C^{fb,o}\left(\kappa \right)$ and $C^{fb,o}\left(\kappa ,s\right)$ are independent of $\Sigma_{1}\geq 0$ and s, respectively, and the following identities hold.*
(298) $$\begin{array}{c} C^{fb,o}\left(\kappa \right)=C^{fb,o}\left(\kappa ,s\right)=C^{fb,S,o}\left(\kappa ,s\right),\;\forall s \end{array}$$
  Furthermore, if the set $P^{\infty }\left(\kappa \right)$ is empty, replace stabilizability of $\left\{A^{*},GB^{*,\frac{1}{2}}\right\}$ by unit circle controllability, i.e., so that $K^{\infty }$ is the maximal and stabilizing solutions of the ARE.

**Proof.**  The first part is an application of Theorem 7, Lemmas 4 and 6. Parts (1)–(3) are due to the convergence properties of the Kalman filter (due to the stabilizability and detectability conditions). It remains to show (298). The equality $C^{fb,o}\left(\kappa \right)=C^{fb,o}\left(\kappa ,s\right),\forall s$ holds by Conclusion 1(a). The last equality holds due to the AR(a,c),a∈(−∞,∞),c∈(−∞,∞) noise. If the initial state $S_{1}=S_{1}^{s}=s$ is known to the encoder and the decoder, then Condition 1 of Section 1.1 holds. In addition, Condition 2 also holds, as can be easily verified from Equations (121) and (122).  □

**Remark 21.**  *From Corollary 10, we obtain the degenerate cases, AR(c),c∈(−∞,∞), noise, i.e., setting a=0. The various implications of the detectability and stabilizability conditions for the AR(c),c∈(−∞,∞) noise are found in [1]. The corresponding $C^{fb,o}\left(\kappa ,s\right)$ states that for stable AR(c) and time-invariant strategies, feedback does not increase capacity (because of the stronger condition of stabilizabily). However, if unit circle controllability is imposed instead, then feedback increases capacity.*

## 4. Sequential Characterization of *n*–FTFI Capacity for Case (II) Formulation

In this section, we consider the Case (II) formulation, and we derive the characterization of feedback capacity, $C_{n}^{fb,S}\left(\kappa ,s\right)$, of the AGN channel (1) driven by a noise $V^{n}$ of Definition 2, i.e., for the code of Definition 3, $\left(s,2^{nR},n\right)$, n=1,2,…, when Conditions 1 and 2 of Section 1.1 hold.

**Definition 8.**  *AGN channels driven by noise with invertible PO-SS realizations*

*The PO-SS realization of the noise of Definition 2 is called invertible if it satisfies the following condition:*
*(A1) Given the initial state $S_{1}=S_{1}^{s}=s$, the noise $V^{t-1}$ uniquely specifies the state $S^{t}$, for t=1,…,n, and vice versa.*

**Corollary 11.**  *Characterization of n–FTFI Capacity for the Case (II) formulation*

*Consider the AGN channel (1) driven by a noise $V^{n}$ of Definition 8, and the code of Definition 3, $\left(s,2^{nR},n\right)$, n=1,2,…, i.e., Conditions 1 and 2 of Section 1.1 hold.*
*Define the n–FTFI capacity for a fixed initial state $S_{1}=S_{1}^{s}=s$ by*
(299) $$\begin{array}{c} C_{n}^{fb,S}\left(\kappa ,s\right)=\underset{P_{\left[1,n\right]}^{s}\left(\kappa \right)}{\sup}H^{P}\left(Y^{n}|s\right)-H\left(V^{n}|s\right) \end{array}$$
  *where the set $P_{\left[1,n\right]}^{s}\left(\kappa \right)$ is defined by*
(300) $$\begin{array}{c} P_{\left[1,n\right]}^{s}\left(\kappa \right)\overset{▵}{=}\left\{P_{t}\left(dx_{t}|x^{t-1},y^{t-1},s\right),t=1,\ldots ,n:\frac{1}{n}E_{s}^{P}\left(\sum_{t=1}^{n}{\left(X_{t}\right)}^{2}\right)\leq \kappa \right\} \end{array}$$
  *and where $E_{s}^{P}$ means $S_{1}=S_{1}^{s}=s$ is fixed, and the joint distribution depends on the elements of $P_{\left[1,n\right]}^{s}\left(\kappa \right)$.*
*Then, the following hold:*
*(a) The n–FTFI capacity, for a fixed $S_{1}=s$, is characterized by*
(301) $$\begin{array}{cc} \; & C_{n}^{fb,S}\left(\kappa ,s\right)=\underset{{\bar{P}}_{\left[1,n\right]}^{s,M}\left(\kappa \right)}{\sup}\sum_{t=1}^{n}\left\{H^{{\bar{P}}^{M}}\left(Y_{t}|Y^{t-1},s\right)-H\left(V_{t}|V^{t-1},s\right)\right\} \end{array}$$
(302) $$\begin{array}{cc} = & \underset{{\bar{P}}_{\left[1,n\right]}^{s,M}\left(\kappa \right)}{\sup}\sum_{t=1}^{n}\left\{H^{{\bar{P}}^{M}}\left(Y_{t}-E^{{\bar{P}}^{M}}\left\{Y_{t}\left|\right|Y^{t-1},s\right\}|s\right)-H\left(V_{t}-E\left\{V_{t}|V_{t}^{t-1},s\right\}|s\right)\right\} \end{array}$$
  *where the ${\bar{P}}_{\left[1,n\right]}^{s,M}\left(\kappa \right)$ is defined by*
(303) $$\begin{array}{cc} \; & {\bar{P}}_{\left[1,n\right]}^{s,M}\left(\kappa \right)\overset{▵}{=}\left\{{\bar{P}}_{t}^{M}\left(dx_{t}|s_{t},y^{t-1},s\right),t=1,\ldots ,n:\frac{1}{n+1}E_{s}^{{\bar{P}}^{M}}\left(\sum_{t=1}^{n}{\left(X_{t}\right)}^{2}\right)\leq \kappa \right\} \end{array}$$
  *and where (18) is respected, ${\bar{P}}_{t}^{M}\left(dx_{t}|s_{t},y^{t-1},s\right)$, is conditionally Gaussian, with linear conditional mean and nonrandom conditional covariance, given by (the notation $S_{t}=S_{t}^{s},t=2,\ldots ,n$ means this sequence is generated from (19), when the initial state is fixed, $S_{1}=S_{1}^{s}=s$).*
(304) $$\begin{array}{cc} \; & E^{{\bar{P}}^{M}}\left\{X_{t}|S_{t}^{s},Y^{t-1},S_{1}^{s}=s\right\}=\left\{\begin{array}{cc} \Lambda_{t}\left(S_{t}^{s}-E^{{\bar{P}}^{M}}\left\{S_{t}^{s}|Y^{t-1},S_{1}^{s}=s\right\}\right), & t=2,\ldots ,n\\ 0, & t=1, \end{array}\right. \end{array}$$
(305) $$\begin{array}{cc} \; & K_{X_{t}|S_{t}^{s},Y^{t-1},S_{1}^{s}=s}\overset{▵}{=}cov\left(X_{t},X_{t}|S_{t}^{s},Y^{t-1},S_{1}^{s}=s\right)=K_{Z_{t}}⪰0,\;t=1,\ldots ,n. \end{array}$$
  *and $H^{\bar{P}}\left(Y_{t}|Y^{t-1},s\right)$ is evaluated with respect to the probability distribution $P_{t}^{{\bar{P}}^{M}}\left(dy_{t}|y^{t-1},s\right)$, defined by*
(306) $$\begin{array}{c} P_{t}^{{\bar{P}}^{M}}\left(dy_{t}|y^{t-1},s\right)=\int P_{t}\left(dy_{t}|x_{t},s_{t}\right)\;P_{t}^{{\bar{P}}^{M}}\left(dx_{t}|s_{t},y^{t-1},s\right)\;P_{t}^{{\bar{P}}^{M}}\left(ds_{t}|y^{t-1},s\right),\;t=1,\ldots ,n. \end{array}$$
  *(b) Define the conditional means and conditional covariance for a fixed $S_{1}=S_{1}^{s}=s$ by*
(307) $$\begin{array}{cc} K_{t}^{s}\overset{▵}{=} & cov(S_{t}^{s},S_{t}^{s}|Y^{t-1},S_{1}^{s}=s)=E_{s}^{{\bar{P}}^{M}}\left\{\left(S_{t}^{s}-{\hat{S}}_{t}^{s}\right){\left(S_{t}^{s}-{\hat{S}}_{t}^{s}\right)}^{T}\right\}, \end{array}$$
(308) $$\begin{array}{cc} {\hat{S}}_{t}^{s}\overset{▵}{=} & E_{s}^{{\bar{P}}^{M}}\left\{S_{t}^{s}|Y^{t-1},S_{1}^{s}=s\right\},\;t=2,\ldots ,n,\;K_{1}^{s}\overset{▵}{=}cov(S_{1}^{s},S_{1}^{s}\left|S_{1}^{s}=s\right)=0,\;{\hat{S}}_{1}^{s}\overset{▵}{=}s. \end{array}$$
  *The optimal channel input distribution of part (a) is induced by a jointly Gaussian process $X^{n}$, with a realization given by*
(309) $$\begin{array}{cc} \; & X_{t}=\Lambda_{t}\left(S_{t}^{s}-{\hat{S}}_{t}^{s}\right)+Z_{t},\;X_{1}=Z_{1},\;t=2,\ldots ,n, \end{array}$$
(310) $$\begin{array}{cc} \; & Z_{t}\in N\left(0,K_{Z_{t}}\right)\;independent\;of\;\left(S_{1},X^{t-1},V^{t-1},Y^{t-1}\right),\;t=1,\ldots ,n, \end{array}$$
(311) $$\begin{array}{cc} \; & Y_{t}=\Lambda_{t}\left(S_{t}^{s}-{\hat{S}}_{t}^{s}\right)+Z_{t}+V_{t},\;t=1,\ldots ,n, \end{array}$$
(312) $$\begin{array}{cc} \; & \;\;=\Lambda_{t}\left(S_{t}^{s}-{\hat{S}}_{t}^{s}\right)+C_{t}S_{t}^{s}+N_{t}W_{t}+Z_{t}, \end{array}$$
(313) $$\begin{array}{cc} \; & \frac{1}{n}E_{s}^{{\bar{P}}^{M}}\left\{\sum_{t=1}^{n}{\left(X_{t}\right)}^{2}\right\}=\frac{1}{n}\sum_{t=1}^{n}\left(\Lambda_{t}K_{t}^{s}\Lambda_{t}^{T}+K_{Z_{t}}\right)\leq \kappa \end{array}$$
  *where $\Lambda_{t}$ is nonrandom.*
*The conditional means and conditional covariance ${\hat{S}}_{t}^{s}$ and $K_{t}^{s}$ are given by the generalized Kalman filter, as follows:*
*(i) ${\hat{S}}_{t}^{s}$ satisfies the Kalman filter recursion*
(314) $$\begin{array}{cc} \; & {\hat{S}}_{t+1}^{s}=A_{t}{\hat{S}}_{t}^{s}+F_{t}\left(K_{t}^{s}\right)I_{t}^{s},\;{\hat{S}}_{1}=s,\\ \; & F_{t}\left(K_{t}^{s}\right)\overset{▵}{=}\left(A_{t}K_{t}^{s}{\left(\Lambda_{t}+C_{t}\right)}^{T}+B_{t}K_{W_{t}}N_{t}^{T}\right) \end{array}$$
(315) $$\begin{array}{cc} \; & .{\left\{N_{t}K_{W_{t}}N_{t}^{T}+K_{Z_{t}}+\left(\Lambda_{t}+C_{t}\right)K_{t}^{s}{\left(\Lambda_{t}+C_{t}\right)}^{T}\right\}}^{-1}, \end{array}$$
(316) $$\begin{array}{cc} \; & I_{t}^{s}\overset{▵}{=}Y_{t}-C_{t}{\hat{S}}_{t}^{s}=\left(\Lambda_{t}+C_{t}\right)\left(S_{t}^{s}-{\hat{S}}_{t}^{s}\right)+N_{t}W_{t}+Z_{t},\;t=1,\ldots ,n, \end{array}$$
(317) $$\begin{array}{cc} \; & I_{t}^{s}\in N\left(0,K_{I_{t}^{s}}\right),\;t=1,\ldots ,n\;an\;orthogonal\;innovations\;process,\;i.e.,\\ \; & \;\;I_{t}^{s}\;is\;independent\;of\;I_{k}^{s},\;for\;all\;t\neq k,\;and\;I_{t}^{s}\;is\;independent\;of\;Y^{t-1}, \end{array}$$
(318) $$\begin{array}{cc} \; & K_{Y_{t}|Y^{t-1},s}=K_{I_{t}^{s}}\overset{▵}{=}cov\left(I_{t},I_{t}|S_{1}^{s}=s\right)\\ \; & =\left(\Lambda_{t}+C_{t}\right)K_{t}^{s}{\left(\Lambda_{t}+C_{t}\right)}^{T}+N_{t}K_{W_{t}}N_{t}^{T}+K_{Z_{t}}. \end{array}$$
*(ii) The error $E_{t}^{s}\overset{▵}{=}S_{t}^{s}-{\hat{S}}_{t}^{s}$ satisfies the recursion*
(319) $$\begin{array}{cc} E_{t+1}^{s}= & F_{t}^{CL}\left(K_{t}^{s}\right)E_{t}^{s}+\left(B_{t}-F_{t}\left(K_{t}^{s}\right)N_{t}\right)W_{t}-F_{t}\left(K_{t}^{s}\right)Z_{t},\;E_{1}^{s}=S_{1}^{s}-{\hat{S}}_{1}^{s}=0,\;t=1,\ldots ,n, \end{array}$$
(320) $$\begin{array}{cc} F_{t}^{CL}( & K_{t}^{s})\overset{▵}{=}A_{t}-F_{t}\left(K_{t}^{s}\right)\left(\Lambda_{t}+C_{t}\right). \end{array}$$
  *(iii) $K_{t}^{s}=E\left\{E_{t}^{s}{\left(E_{t}^{s}\right)}^{T}\right\}$ satisfies the generalized DRE*
(321) $$\begin{array}{cc} \; & K_{t+1}^{s}=A_{t}K_{t}A_{t}^{T}+B_{t}K_{W_{t}}B_{t}^{T}-\left(B_{t}K_{W_{t}}N_{t}^{T}+A_{t}K_{t}^{s}{\left(\Lambda_{t}+C_{t}\right)}^{T}\right)\{N_{t}K_{W_{t}}N_{t}^{T}+K_{Z_{t}}\\ \; & +\left(\Lambda_{t}+C_{t}\right)K_{t}^{s}{\left(\Lambda_{t}+C_{t}\right)}^{T}{\}}^{-1}{\left(B_{t}K_{W_{t}}N_{t}^{T}+A_{t}K_{t}^{s}\left(\Lambda_{t}+C_{t}\right)\right)}^{T},\;K_{t}^{s}⪰0,\;K_{1}^{s}=0,\;t=1,\ldots ,n. \end{array}$$
  *(c) The characterization of the n–FTFI capacity of part (a) is*
(322) $$\begin{array}{cc} \; & C_{n}^{fb,S}\left(\kappa ,s\right)=\underset{\left(\Lambda_{t},K_{Z_{t}}\right),t=1,\ldots ,n:\;\frac{1}{n}E_{s}\left\{\sum_{t=1}^{n}{\left(X_{t}\right)}^{2}\right\}\leq \kappa }{\sup}\sum_{t=1}^{n}\log\frac{K_{Y_{t}|Y^{t-1},s}}{K_{V_{t}|V^{t-1},s}} \end{array}$$
(323) $$\begin{array}{cc} \; & =\underset{\left(\Lambda_{t},K_{Z_{t}}\right),t=1,\ldots ,n:\;\frac{1}{n}E_{s}\left\{\sum_{t=1}^{n}{\left(X_{t}\right)}^{2}\right\}\leq \kappa }{\sup}\sum_{t=1}^{n}\left\{H\left(I_{t}^{s}\right)-H\left(N_{t}W_{t}\right)\right\} \end{array}$$
(324) $$\begin{array}{cc} \; & =\underset{\left(\Lambda_{t},K_{Z_{t}}\right),t=1,\ldots ,n:\;\frac{1}{n}\sum_{t=1}^{n}\left(\Lambda_{t}K_{t}^{s}\Lambda_{t}^{T}+K_{Z_{t}}\right)\leq \kappa }{\sup}\{\\ \; & \frac{1}{2}\sum_{t=1}^{n}\log\left(\frac{\left(\Lambda_{t}+C_{t}\right)K_{t}^{s}{\left(\Lambda_{t}+C_{t}\right)}^{T}+N_{t}K_{W_{t}}N_{t}^{T}+K_{Z_{t}}}{N_{t}K_{W_{t}}N_{t}^{T}}\right)\}. \end{array}$$
  *and the statements of part (b) hold.*

**Proof.**  See Appendix A.8.  □

**Remark 22.**  *The asymptotic analysis of Section 3, based on Definition 4, applies naturally to Corollary 11.*

**Corollary 12.**  *Characterization of Feedback Capacity for the Case (II) formulation*

*Consider the statement of Corollary 11 for asymptotically time-invariant noise. The feedback capacity is given by*
(325) $$\begin{array}{cc} \; & C^{fb,S}\left(\kappa ,s\right)=\lim_{n⟶\infty }\frac{1}{n}C_{n}^{fb,S}\left(\kappa ,s\right) \end{array}$$
(326) $$\begin{array}{cc} \; & =\underset{\left(\Lambda^{\infty },K_{Z}^{\infty }\right)\in P^{\infty ,ucc,S}\left(\kappa \right)}{\sup}\frac{1}{2}\log\left(\frac{\left(\Lambda^{\infty }+C\right)K^{\infty }{\left(\Lambda^{\infty }+C\right)}^{T}+NK_{W}N^{T}+K_{Z}^{\infty }}{NK_{W}N^{T}}\right), \end{array}$$
(327) $$\begin{array}{cc} \; & P^{\infty ,ucc,S}\left(\kappa \right)\overset{▵}{=}\{\left(\Lambda^{\infty },K_{Z}^{\infty }\right):K_{Z}^{\infty }\geq 0,\;\Lambda^{\infty }K^{\infty }{\left(\Lambda^{\infty }\right)}^{T}+K_{Z}^{\infty }\leq \kappa ,\;K^{\infty }\;is\;the\;maximal\;and\\ \; & stabilizing\;solutionof\;\text{(328)},\;i.e.,\;detectability\;and\;unit\;circle\;controllability\;hold\}, \end{array}$$
(328) $$\begin{array}{cc} \; & K^{\infty }=AK^{\infty }A^{T}+BK_{W}B^{T}-\left(BK_{W}N^{T}+AK^{\infty }{\left(\Lambda^{\infty }+C\right)}^{T}\right)\{NK_{W}N^{T}+K_{Z}^{\infty }\\ \; & +\left(\Lambda^{\infty }+C\right)K^{\infty }{\left(\Lambda^{\infty }+C\right)}^{T}{\}}^{-1}{\left(BK_{W}N^{T}+AK^{\infty }\left(\Lambda^{\infty }+C\right)\right)}^{T}. \end{array}$$
*If the optimal solution is such that the stabilizability holds, then the feedback capacity is independent of the initial state $S_{1}=s$.*

**Proof.**  This is a special case of Theorem 7, with $\Sigma^{\infty }=0$.  □

**Corollary 13.**  *Feedback capacity of [4,7]*

*Consider the channels studied in [4,7], i.e., with time-invariant and stable realization.*
*(a) The time-domain n–FTFI capacity is $C_{n}^{fb,S}\left(\kappa ,s\right)$ given in Corollary 11.*
*(b) The time-domain feedback capacity is given by Corollary 12.*

**Proof.**  Since the noise in [4,7] is time-invariant and stable, according to Definition 8, and the code is that of Definition 3, i.e., $\left(s,2^{nR},n\right)$, n=1,2,…, then their results are special cases of Corollaries 11 and 12.  □

In the next remark, we clarify the relation of Corollary 11 and the analysis of [4,8].

**Remark 23.**  *Relations of Corollary 11 and [4,8]*

*(a) The state space problem analyzed in [8] is precisely $C_{n}^{fb,S}\left(\kappa ,s\right)$, when the noise is stationary and Gaussian, i.e., it corresponds to the Case (II) formulation. Corollary 11 is derived in [8] for the degenerate case of a time-invariant realization of the noise $V^{n}$, i.e., of Definition 8. However, the asymptotic analysis of [8] (Section VI) should be read with caution, because it did not impose the necessary and/or sufficient conditions for convergence of the sequence $K_{t}^{s},t=1,2,\ldots$ generated by the time-invariant version of the generalized DRE (321), i.e., $\lim_{n⟶\infty }K_{n}^{s}=K^{\infty }⪰0$, where $K^{\infty }⪰0$ is either the maximal or the unique and stabilizing solution of a corresponding generalized ARE.*
*(b) The problem analyzed [4] that led to [4] (Theorem 6.1, $C_{FB}$) is the per unit time limit of $C_{n}^{fb,S}\left(\kappa ,s\right)$, when the noise is stationary, two-sided or one-sided (asymptotically stationary) and Gaussian, i.e., it corresponds to the Case (II) formulation. The characterization of feedback capacity presented in [4] (Theorem 6.1, $C_{FB}$) presupposed that the following hold ((i)–(iii) are also assumed in [8] (Section VI)):*
*(i) The feedback code is Definition 3, i.e., $\left(s,2^{nR},n\right)$.*
*(ii) The noise is time-invariant and stable, and the PO-SS realization of the noise is invertible, as presented in Definition 8.*
*(iii) The definition of rate is $C^{fb,S,o}\left(\kappa ,s\right)$, with supremum and per unit time limit interchanged, and the supremum taken over using time-invariant channel input distributions.*
*(iv) The innovations covariance of the channel input process is asymptotically zero, i.e., $\lim_{n⟶\infty }K_{Z_{n}}=K_{Z}^{\infty }=0$. This implies the corresponding $\lim_{n⟶\infty }K_{n}^{s}=K^{\infty }\geq 0$ is the maximal and stabilizing solution of the corresponding matrix ARE, since detectability and unit circle controllability conditions hold, but not the stabilizability condition.*
*Items (i)–(iv) are confirmed from [4] (Lemma 6.1) (and comments above), which is used to derive [4] (Theorem 6.1, $C_{FB}$).*
*However, the characterization of feedback capacity in [4] (Theorem 6.1, $C_{FB}$) should be read with caution, because the stabilizability condition is violated, due to the requirement of the author, which states that $K_{Z}^{\infty }=0$ is optimal. By Theorem 5(1), for the choice $K_{Z}^{\infty }=0$, the only choice is the maximal and stabilizing solution of the generalized ARE presented in [4] (Theorem 6.1, $C_{FB}$).*
*However, it is easy to verify that [4] (Theorem 6.1, C_FB_) cannot be the capacity of asymptotically stationary noise because $C_{FB}$ depends on the covariance $K_{S_{1}}≻0$. Moreover, $K_{1}≻0$ is required.*
*Finally, we emphasize that the treatment of the ARMA noise in [2] clarifies the above issues.*

## 5. Conclusions

New equivalent sequential characterizations of Cover and Pombra [5] “*n*–block” feedback capacity formulas are derived using time-domain methods for additive Gaussian noise (AGN) channels driven by non-stationary Gaussian noise. New features of the equivalent characterizations encompass the representation of the optimal channel input process by a *sufficient statistic and Gaussian orthogonal innovations process*. The sequential characterizations of the *n*–block feedback capacity formula are expressed as a functional of two generalized matrix difference Riccati equations (DREs) of the filtering theory of Gaussian systems. The asymptotic analysis of the per unit time limit of the *n*–block”, called feedback capacity, is also presented for time-invariant and asymptotically time-invariant channel input distributions, using the tools from the theory of generalized matrix Riccati equations.

Prior analysis and characterizations of feedback capacity follows on from the analysis and derivation of the new sequential characterizations of feedback capacity, such as [4,7,11,12], who do not address the Cover and Pombra [5] feedback capacity problem, as the code definitions and noise assumptions in [4,7,11,12] (even under the restriction of stationary noise) are fundamentally different from those in [5]. This paper clarifies several of these points of confusion.

## Data Availability

No data are contained within this article.

## Appendix A

### Appendix A.1. Proof of Theorem 1

(a) Consider an element of ${\bar{E}}_{\left[0,n\right]}\left(\kappa \right)$. Then, the conditional entropies $H^{\bar{e}}\left(Y_{t}|Y^{t-1}\right),t=1,\ldots ,n$ are defined, provided that the conditional distributions of $Y_{t}$ conditioned on $Y^{t-1}$, i.e., $P_{t}^{\bar{e}}\left(dy_{t}|y^{t-1}\right)$, for t=1,…,n, are determined. By the reconditioning, using (60), we have

(A1) $$\begin{array}{cc} P_{t}^{\bar{e}} & \left(dy_{t}|y^{t-1}\right)=\int P_{t}^{\bar{e}}\left(dy_{t}|y^{t-1},w\right)\;P_{t}^{\bar{e}}\left(dw|y^{t-1}\right),\;t=0,\ldots ,n \end{array}$$

(A2) $$\begin{array}{cc} = & \int P_{t}\left(dy_{t}|{\bar{e}}_{t}\left(w,v^{t-1},y^{t-1}\right),v^{t-1}\right)\;P_{t}^{\bar{e}}\left(dw|y^{t-1}\right),\;\mathrm{by}\;\text{(60)},\;\text{(61)}. \end{array}$$

Hence, (64) is shown. Similarly, consider an element of ${\bar{P}}_{\left[0,n\right]}\left(\kappa \right)$. Then, the conditional entropies $H^{\bar{P}}\left(Y_{t}|Y^{t-1}\right),t=1,\ldots ,n$ are defined, provided that the conditional distributions of $Y_{t}$ conditioned on $Y^{t-1}$, i.e., $P_{t}^{\bar{P}}\left(dy_{t}|y^{t-1}\right)$ for t=1,…,n, are determined. By (52) and (53), (65) is obtained. Since ${\bar{E}}_{\left[0,n\right]}\left(\kappa \right)\subseteq {\bar{P}}_{\left[0,n\right]}\left(\kappa \right)$, Inequality (63) follows.

(b) This part is followed by the maximum entropy principle of Gaussian distributions. That is, under the restriction (18), a conditional Gaussian element of $\left\{\bar{P}\left(dx_{t}|v^{t-1},y^{t-1}\right),t=1,\ldots ,n\right\}\in {\bar{P}}_{\left[0,n\right]}\left(\kappa \right)$ with linear conditional mean and nonrandom conditional covariance induces a jointly Gaussian distribution of the process $\left(X^{n},Y^{n}\right)$, such that the marginal distribution of $Y^{n}$ is jointly Gaussian. Below, we provide alternative proof that uses the Cover and Pombra characterization of the *n*–FTFI capacity, given by (34) and (35). Consider (35) and define the process

(A3) $$\begin{array}{cc} \;Z_{1}\overset{▵}{=} & {\bar{Z}}_{1}-E\left\{{\bar{Z}}_{1}\right\}, \end{array}$$

(A4) $$\begin{array}{cc} Z_{t}\overset{▵}{=} & {\bar{Z}}_{t}-E^{\bar{P}}\left\{{\bar{Z}}_{t}|X^{t-1},V^{t-1},Y^{t-1}\right\},\;t=2,\ldots ,n, \end{array}$$

(A5) $$\begin{array}{cc} = & {\bar{Z}}_{t}-E^{\bar{P}}\left\{{\bar{Z}}_{t}|V^{t-1},Y^{t-1}\right\},\;\mathrm{since}X^{t-1}\mathrm{is}\mathrm{uniquely}\mathrm{defined}\mathrm{by}\left(V^{t-1},Y^{t-1}\right). \end{array}$$

Then, $Z_{t}$ is a Gaussian orthogonal innovations process, independent of $\left(X^{t-1},V^{t-1},Y^{t-1}\right)$, for t=2,…,n, and $E\left\{Z_{t}\right\}=0$, for t=1,…,n. By (35), we re-write $X_{t},t=1,\ldots ,n$ as,

(A6) $$\begin{array}{cc} X_{t}= & \sum_{j=1}^{t-1}B_{t,j}V_{j}+{\bar{Z}}_{t},\;t=1,\ldots ,n, \end{array}$$

(A7) $$\begin{array}{cc} = & \sum_{j=1}^{t-1}B_{t,j}V_{j}+E^{\bar{P}}\left\{{\bar{Z}}_{t}|V^{t-1},Y^{t-1}\right\}+Z_{t},\;by() \end{array}$$

(A8) $$\begin{array}{cc} \overset{\left(a\right)}{=} & \sum_{j=1}^{t-1}B_{t,j}V_{j}+{\bar{\Gamma }}_{t}\left(\begin{array}{c} V^{t-1}\\ Y^{t-1} \end{array}\right)+Z_{t},\;\mathrm{for}\mathrm{some}{\bar{\Gamma }}_{t}\mathrm{nonrandom} \end{array}$$

(A9) $$\begin{array}{cc} = & \sum_{j=1}^{t-1}\Gamma_{t,j}^{1}V_{j}+\sum_{j=1}^{t-1}\Gamma_{t,j}^{2}Y_{j}+Z_{t},\;\mathrm{for}\mathrm{some}(\Gamma_{\cdot ,\cdot }^{1},\Gamma_{\cdot ,\cdot }^{2}) \end{array}$$

(A10) $$\begin{array}{cc} = & \Gamma_{t}^{1}V^{t-1}+\Gamma_{t}^{2}Y^{t-1}+Z_{t},\;\mathrm{by}\mathrm{definition} \end{array}$$

where (a) is due to the by joint Gaussianity of $\left(Z^{n},X^{n},Y^{n}\right)$. From (A10) and the independence of $Z_{t}$ and $\left(X^{t-1},V^{t-1},Y^{t-1}\right)$, for t=2,…,n, (66) then follows, as well as (67).

(c) The statements follow directly from the representation of part (b), while the independence of $Z^{n}$ and $V^{n}$ is due to the code definition, i.e., Definition 1(iv).
  (d) The statement follows on from parts (a–c) and (28)–(31).

### Appendix A.2. Proof of Proposition 1

(a) The covariances of the realization of the ARMA(a,c) noise of Example 2(b) satisfy the recursions

(A11) $$\begin{array}{c} K_{S_{t+1}}=c^{2}K_{S_{t}}+K_{W},\;K_{S_{t},V_{t}}=\left(c-a\right)K_{S_{t}},\;K_{V_{t}}={\left(c-a\right)}^{2}K_{S_{t}}+K_{W},\;\forall t\in Z. \end{array}$$

If the recursion $K_{S_{t+1}}=c^{2}K_{S_{t}}+K_{W}$ is initiated at the stationary value $K_{S_{1}}=d_{11}=\frac{K_{W}}{1-c^{2}}$, then $K_{S_{t+1}}=d_{11},\forall t=2,3,\ldots$; hence, $S_{t},\forall t\in Z$ is stationary, which then implies the stationarity of $V_{t},\forall t\in Z$. Hence, if (133) holds, then $(V_{t},S_{t}),\forall t\in Z$ is stationary. Via simple calculations, (134) then follows. We carry out the same operation for the one-sided ARMA(a,c). (b) By the above covariances, for all $K_{S_{1}}\geq 0$, we have $\lim_{n⟶\infty }K_{S_{n}}=K_{S}^{\infty }$, where $K_{S}^{\infty }=c^{2}K_{S}^{\infty }+K_{W}$, which then implies $K_{S}^{\infty }=d_{11}$. Similarly, $\lim_{n⟶\infty }K_{S_{n},V_{n}}=K_{S,V}^{\infty }=d_{12}$, $\lim_{n⟶\infty }K_{V_{n}}=K_{V}^{\infty }=d_{22}$. (c) (135) and (136) follow on from Lemma 1 by replacing the conditioning information $V^{t-1}$ with $\left(V_{0},V^{t-1}\right)$ in (85). By mean square estimation, the initial data are

(A12) $$\begin{array}{cc} {\hat{S}}_{1}= & E\left\{S_{1}|V_{0}\right\}=E\left\{cS_{0}+W_{0}|V_{0}\right\}\\ = & E\left\{cS_{0}+W_{0}\right\}+cov\left(cS_{0}+W_{0},V_{0}\right){\left\{cov(V_{0},V_{0})\right\}}^{-1}\left(V_{0}-E\left\{V_{0}\right\}\right)\\ = & \left(cd_{12}+K_{W}\right)d_{22}^{-1}V_{0}, \end{array}$$

(A13) $$\begin{array}{cc} \Sigma_{1}= & cov\left(S_{1},S_{1}|V_{0}\right)=cov\left(S_{1},S_{1}\right)-{\left\{cov(S_{1},S_{1})\right\}}^{2}{\left\{cov(V_{0},V_{0})\right\}}^{-1}\\ = & d_{11}-d_{11}^{2}d_{22}^{-1} \end{array}$$

The last part is obvious.

### Appendix A.3. Proof of Corollary 3

(a) Since we have assumed that $S_{1}=S_{1}^{s}=s$ is fixed, and that it is known to the encoder and the decoder, then Theorem 1 still holds. This is due to replacing all conditional distributions, expectations and entropies via corresponding expressions with a fixed $S_{1}=S_{1}^{s}=s$. Hence, (75) is replaced by (141), and (68) is replaced by (142) (since the code is allowed to depend on $S_{1}=S_{1}^{s}=s$). (b) From the PO-SS realization of Definition 2 with $S_{1}=S_{1}^{s}=s$ fixed, it follows that a necessary condition for Conditions 1 of Section 1.1 to hold is (i). The expression of entropy (143) is easily obtained by invoking Condition (i) and the properties of conditional entropy. That is, $H\left(V_{1}|S_{1}^{s}=s\right)=H\left(C_{1}S_{1}^{s}+N_{1}W_{1}|S_{1}^{s}=s\right)=H\left(N_{1}W_{1}|S_{1}=s\right)=H\left(N_{1}W_{1}\right)$ by independence of $W_{1}$ and $S_{1}^{s}$, and $H\left(V_{2}|V_{1},s\right)=H\left(V_{2}|V_{1},S_{1}^{s}=s\right)=H\left(C_{2}S_{2}^{s}+N_{2}W_{2}|C_{1}S_{1}^{s}+N_{1}W_{1},S_{1}^{s}=s\right)=H\left(C_{2}S_{2}^{s}+N_{2}W_{2}|N_{1}W_{1},S_{1}^{s}=s\right)=H\left(C_{2}A_{1}S_{1}^{s}+C_{2}B_{1}W_{1}+N_{2}W_{2}|N_{1}W_{1},S_{1}^{s}=s\right)=H\left(N_{2}W_{2}|N_{1}W_{1},S_{1}^{s}=s\right)=H\left(N_{2}W_{2}\right)$, etc. The last statement is obvious. This completes the proof.

### Appendix A.4. Proof of Theorem 3

(a) Clearly, (154)–(165) follow directly from Theorem 1 and the preliminary calculations prior to the statement of the theorem. However, (154)–(165) can also be shown independently of Theorem 1 by invoking the maximum entropy property of Gaussian distributions, as follows: By Lemma 1, we have $H\left(V^{n}\right)=\sum_{t=1}^{n}H\left({\hat{I}}_{t}\right)$. By the maximum entropy principle, $H(Y^{n})$ is maximized if $P_{Y^{n}}$ is jointly Gaussian, the average power constraint holds, and (17) is respected. By (146), (150), and (160), if (150)–(165) hold, then $\left(X^{n},Y^{n}\right)$ is jointly Gaussian; hence, $H(Y^{n})$ is maximized. This shows (a).

(b) Step 1. By (163) and (164), an alternative representation of $X^{n}$ to the one given in Theorem 1 and induced by (68) is
  (A14) $$\begin{array}{cc} \; & X_{t}=\Gamma_{t}^{1}{\hat{S}}_{t}+\Gamma_{t}^{2}Y^{t-1}+Z_{t},\;t=1,\ldots ,n, \end{array}$$
  (A15) $$\begin{array}{cc} \; & Z_{t}\;\mathrm{satisfies}\;\text{(167)}. \end{array}$$
  for some nonrandom $\left(\Gamma_{\cdot }^{1},\Gamma_{\cdot }^{2}\right)$. Upon substituting (A14) into the channel output $Y^{n}$, we have
  (A16) $$\begin{array}{cc} Y_{t}= & \Gamma_{t}^{1}{\hat{S}}_{t}+\Gamma_{t}^{2}Y^{t-1}+Z_{t}+V_{t},\;t=1,\ldots ,n \end{array}$$
  (A17) $$\begin{array}{cc} = & \left(\Gamma_{t}^{1}+C_{t}\right){\hat{S}}_{t}+\Gamma_{t}^{2}Y^{t-1}+Z_{t}+{\hat{I}}_{t},\;\mathrm{by}\;\text{(90)}. \end{array}$$
  The right-hand side of (A17) is driven by two independent processes, $Z_{t},t=1,\ldots ,n$ and ${\hat{I}}_{t},t=1,\ldots ,n$, which are also mutually independent. Further, the right-hand side of (A17) is a linear function of a state process ${\hat{S}}_{t},t=1,\ldots ,n$, which satisfies the following recursion (88):
  (A18) $$\begin{array}{c} {\hat{S}}_{t+1}=A_{t}{\hat{S}}_{t}+M_{t}\left(\Sigma_{t}\right){\hat{I}}_{t},\;{\hat{S}}_{1}=\mu_{S_{1}}, \end{array}$$
  Note that the right-hand side of (A18) is driven by the orthogonal process ${\hat{I}}_{t}$, which is independent of $V^{t-1}$ and hence of ${\hat{S}}_{t}$. It is also independent of $Y^{t-1}$. By (167), $Z_{t}$ is independent of $Y^{t-1}$ and of ${\hat{S}}_{t}$. By (A17) and (A18), it follows that ${\hat{\hat{S}}}_{t},t=1,\ldots ,n$ satisfies a generalized Kalman filter recursion, similar to that of Lemma 1. Hence, the entropy $H(Y^{n})$ can be computed using the innovations process of $Y^{n}$, as in Lemma 1. Define the orthogonal Gaussian innovations process $I^{n}$ of $Y^{n}$ by
  (A19) $$\begin{array}{cc} I_{t}\overset{▵}{=} & Y_{t}-E\left\{Y_{t}|Y^{t-1}\right\},\;t=1,\ldots ,n \end{array}$$
  (A20) $$\begin{array}{cc} = & \left(\Gamma_{t}^{1}+C_{t}\right)\left({\hat{S}}_{t}-{\hat{\hat{S}}}_{t}\right)+{\hat{I}}_{t}-E\left\{{\hat{I}}_{t}|Y^{t-1}\right\}+Z_{t},\;\mathrm{by}\;\text{(A17)}. \end{array}$$
  (A21) $$\begin{array}{cc} = & \left(\Gamma_{t}^{1}+C_{t}\right)\left({\hat{S}}_{t}-{\hat{\hat{S}}}_{t}\right)+{\hat{I}}_{t}+Z_{t},\;\mathrm{by}{\hat{I}}_{t}\mathrm{indep}.\;\mathrm{of}Y^{t-1},E\left\{{\hat{I}}_{t}\right\}=0 \end{array}$$
  The entropy of $Y^{n}$ is computed as follows.
  (A22) $$\begin{array}{cc} H(Y^{n})= & \sum_{t=1}^{n}H\left(Y_{t}|Y^{t-1}\right) \end{array}$$
  (A23) $$\begin{array}{cc} = & \sum_{t=1}^{n}H\left(I_{t}|Y^{t-1}\right),\;\text{by (A19) and a property of conditional entropy}; \end{array}$$
  (A24) $$\begin{array}{cc} = & \sum_{t=1}^{n}H\left(I_{t}\right),\;\text{by orthogonality of}\;I_{t}\;\text{and}\;Y^{t-1}. \end{array}$$
  By (A21), the Gaussian innovations process $I^{n}$ does not depend on the strategy of $\Gamma^{2}$. Consequently, by (A24), the entropy $H(Y^{n})$ does not depend on the strategy of $\Gamma^{2}$.
  Step 2. Let $g_{t}\left(Y^{t-1}\right)\overset{▵}{=}\Gamma_{t}^{2}Y^{t-1},t=1,\ldots ,n$. By (A14) and (A15), it then follows that
  (A25) $$\begin{array}{cc} \frac{1}{n}E & \left\{\sum_{t=1}^{n}{\left(X_{t}\right)}^{2}\right\}=\frac{1}{n}E\left\{\sum_{t=1}^{n}{\left(\Gamma_{t}^{1}{\hat{S}}_{t}+g_{t}\left(Y^{t-1}\right)+Z_{t}\right)}^{2}\right\} \end{array}$$
  (A26) $$\begin{array}{cc} = & \frac{1}{n}E\left\{\sum_{t=1}^{n}{\left(\Gamma_{t}^{1}{\hat{S}}_{t}+g_{t}\left(Y^{t-1}\right)\right)}^{2}\right\}+\sum_{t=1}^{n}K_{Z_{t}},\;\text{by indep. of}\;Z_{t}\;\mathrm{and}\;\left(V^{t-1},{\hat{S}}_{t},Y^{t-1}\right). \end{array}$$
  By utilizing the mean square estimation theory, then the choice of g(·) that minimizes the right hand of (A26) is
  (A27) $$\begin{array}{c} g_{t}\left(Y^{t-1}\right)=g_{t}^{*}\left(Y^{t-1}\right)=-\Gamma_{t}^{1}E\left\{{\hat{S}}_{t}|Y^{t-1}\right\}=-\Gamma_{t}^{1}{\hat{\hat{S}}}_{t},\;t=1,\ldots ,n. \end{array}$$
  Hence, $\Gamma_{t}^{2}=-\Gamma_{t}^{1},\forall t$. Let $\Lambda_{t}\overset{▵}{=}\Gamma_{t}^{1},\forall t$ and substitute into the (A17) recursion to obtain (170) and into the average power (A26) to obtain (171). Hence, the derivation of (166)–(171) is completed.

It then follows that (172)–(180) are generalized Kalman filter recursions of estimating the new state process ${\hat{S}}_{t},t=1,\ldots ,n$ that satisfies recursion (A18) from the channel output process $Y_{t}$ that satisfies the recursion (170).

(c) By parts (a) and (b), and the entropy of Gaussian RVs, upon substituting (A24) and (98) into (154), we obtain part (c). This completes the proof.

### Appendix A.5. Proof of Proposition 2

Since the proof of [4] (Theorem 6.1) is based on [4] (Lemma 6.1), where the channel input $X_{t}$ is expressed as $X_{t}=\Lambda (S_{t}-E\left\{S_{t}|Y_{-\infty }^{t-1}\right),t=1,\ldots$, where Λ is a nonrandom vector, then (211) is necessary for [4] (Theorem 6.1) to hold. Next, we show that Conditions 1 and 2 of Section 1.1 are necessary and sufficient for Equality (211) to hold. To avoid a complex notation, we prove the claim for the realization of Example 2(a). Suppose that the *initial state* $S_{1}$ of the noise is $S_{1}=S_{1}^{s}=s$ and that it is known to the encoder and the decoder; without loss of generality, take s=0, which, by (127), implies $V_{0}=0,W_{0}=0$ (as is often carried out in [4]). Then, the following hold:

(A28) $$\begin{array}{cc} \; & S_{1}=0\;⟹\;V_{1}=W_{1},\;S_{2}=W_{1}=V_{1},\;\text{by (128), (129)}; \end{array}$$

(A29) $$\begin{array}{cc} \; & \left(S_{1}=0,V_{1}\right)\;\text{uniquely define}\;S_{2}=cS_{1}+W_{1}=W_{1}=V_{1},\;\text{by (128), (129)}; \end{array}$$

(A30) $$\begin{array}{cc} \; & V_{2}=\left(c-a\right)S_{2}+W_{2},\;S_{3}=cS_{2}+W_{2}=cV_{1}+W_{2},\;\text{by (128), (129)}; \end{array}$$

(A31) $$\begin{array}{cc} \; & \left(S_{1}=0,V_{1},V_{2}\right)\;\text{uniquely define}\;\left(S_{2},S_{3}\right); \end{array}$$

(A32) $$\begin{array}{cc} \; & \text{Repeating},\text{then}\;\left(S_{1}=0,V_{1},\ldots ,V_{t-1}\right)\;\text{uniquely define}\;\left(S_{2},S_{3},\ldots ,S_{t}\right),\forall t=3,4,\ldots . \end{array}$$

From (A28)–(A32), it then follows that for any $S_{1}=s$, including s=0, it is known by the encoder that the following equalities hold:

(A33) $$\begin{array}{cc} P_{X_{t}|X^{t-1},Y_{-\infty }^{t-1},S_{1}}= & P_{X_{t}|V^{t-1},Y_{-\infty }^{t-1},S_{1}},\;byY_{t}=X_{t}+V_{t} \end{array}$$

(A34) $$\begin{array}{cc} = & P_{X_{t}|S^{t},Y_{-\infty }^{t-1},S_{1}},\;t=1,\ldots , \end{array}$$

We can go one step further to identify the information structure of optimal channel input distributions using (A34), i.e., to show $P_{X_{t}|S^{t},Y_{-\infty }^{t-1},S_{1}}=P_{X_{t}|S_{t},Y_{-\infty }^{t-1},S_{1}},\;t=1,\ldots$, by repeating to proof of [8] (Theorem 1). However, for the statement of the proposition, this is not necessary.

Suppose that either $S_{1}=s$ is not known to the encoder, i.e., $V_{0}=v_{0},W_{0}=w_{0}$ are not known to the encoder, and $S_{1}\neq 0$, while the optimal channel input is expressed as a function of the state of the noise, $S^{n}$:

(A35) $$\begin{array}{cc} P_{X_{t}|X^{t-1},Y_{-\infty }^{t-1}}= & P_{X_{t}|V^{t-1},Y_{-\infty }^{t-1}}=P_{X_{t}|S^{t},Y_{-\infty }^{t-1}},\;t=1,\ldots , \end{array}$$

Then, by (128) and (129), it follows that $V_{1}=\left(c-a\right)S_{1}+W_{1},S_{2}=aS_{1}+W_{1}$. Hence, knowledge of $V_{1}$ does not specify $S_{2}$. Similarly, $V^{t-1}$ does not specify $S^{t}$, for t=2,3,…. Hence, we arrive at a contradiction of the last equality in (A35). This competes the proof.

### Appendix A.6. Proof of Lemma 2

(a) This is due to Lemma 1(v).
  (b) By taking the per unit time limit (221) and utilizing the hypothesis (223), the continuity of the log(·) and the fact that, for any convergent sequence $a_{n},n=1,2,\ldots$, i.e., $\lim_{n⟶\infty }a_{n}=a$, then $\frac{1}{n}\sum_{t=1}^{n}a_{n}⟶a$, as n⟶∞, (224) follows.

### Appendix A.7. Proof of Lemma 4

From Corollary 6(a), we deduce that $\Sigma_{t}^{o}\overset{▵}{=}\Sigma_{t},t=1,\ldots ,n$ satisfies (239) with initial condition (240). By Definition 7, the corresponding generalized algebraic Riccati equation is (241), and pairs {A,C} and $\left\{A^{*},GB^{*,\frac{1}{2}}\right\}$ are given by (242).

(1) By Definition 7, for c≠a, the pair {A,C}={c,c−a} is observable and hence detectable.
  (2) By Definition 7, the pair $\left\{A^{*},GB^{*,\frac{1}{2}}\right\}=\left\{a,0\right\}$ is unit circle controllable if and only if |a|≠1.
  (3) By Definition 7, the pair $\left\{A^{*},GB^{*,\frac{1}{2}}\right\}=\left\{a,0\right\}$ is stabilizable if and only if a∈(−1,1).
  (4) This follows from Theorem 5(1) and parts (1)–(4). Since (241) is a quadratic equation, we can verify that the two solutions are $\Sigma^{\infty }=0$ and $\Sigma^{\infty }=\frac{K_{W}\left(a^{2}-1\right)}{{\left(c-a\right)}^{2}}$, and we consider the statement of (243).
  (5) For values c∈(−∞,∞) and |a|<1, the pair {A,C}={c,c−a} is detectable and the pair $\left\{A^{*},GB^{*,\frac{1}{2}}\right\}=\left\{a,0\right\}$ is stabilizable, and the statement follows from Theorem 5(3).
  (6) (245) follows from Lemma 2(b) by invoking Corollary 6(a), i.e., $K_{{\hat{I}}_{t}}={\left(c-a\right)}^{2}\Sigma_{t}^{o}+K_{W},t=1,\ldots ,n$, where $\Sigma_{t}^{o}$ is generated by (239) and parts (4) and (5).

### Appendix A.8. Proof of Corollary 11

First, note that the analog of Theorem 1(a), for the code $\left(s,2^{nR},n\right)$, n=1,2,… is (299) and (300) because $P_{t}\left(dx_{t}|x^{t-1},y^{t-1},s\right)={\bar{P}}_{t}\left(dx_{t}|v^{t-1},y^{t-1},s\right),t=1,\ldots ,n$. Define ${\bar{P}}_{\left[0,n\right]}^{s}\left(\kappa \right)$ as in (300), with $x^{t-1}$ being replaced by $v^{t-1},t=1,\ldots ,n$.

- (a) Then
  (A36) $$\begin{array}{cc} P_{t}\left(dx_{t}|x^{t-1},y^{t-1},s\right)= & P_{t}\left(dx_{t}|v^{t-1},y^{t-1},s_{0}\right),\;t=1,\ldots ,n,\;byY_{t}=X_{t}+V_{t} \end{array}$$
  (A37) $$\begin{array}{cc} = & {\bar{P}}_{t}\left(dx_{t}|s^{t},y^{t-1},s\right),\;\text{Definition 8}. \end{array}$$
  The PO-SS realization, for a fixed $S_{1}^{s}=s$, is then
  (A38) $$\begin{array}{cc} \; & V_{t}=C_{t}S_{t}^{s}+N_{t}W_{t},\;S_{1}^{s}=s,\;t=1,\ldots ,n, \end{array}$$
  (A39) $$\begin{array}{cc} \; & S_{t+1}^{s}=A_{t}S_{t}^{s}+B_{t}W_{t},\;S_{1}^{s}=s. \end{array}$$
  Then,
  (A40) $$\begin{array}{cc} P_{t}\left(dy_{t}|x^{t},y^{t-1},s\right)= & P_{t}\left(dy_{t}|x^{t},v^{t-1},y^{t-1},s\right) \end{array}$$
  (A41) $$\begin{array}{cc} = & P_{t}\left(dy_{t}|x^{t},s^{t},y^{t-1},s\right),\;\text{by Definition 8, (A1)} \end{array}$$
  (A42) $$\begin{array}{cc} = & P_{t}\left(dy_{t}|x_{t},s^{t}\right),\;\mathrm{by}\;Y_{t}=X_{t}+V_{t}\;\text{and (A38)} \end{array}$$
  (A43) $$\begin{array}{cc} \overset{\left(a\right)}{=} & P_{t}\left(dy_{t}|x_{t},s_{t},s\right),\;\text{by mutually independent of}\;(W_{1},\ldots ,W_{n},S_{1}^{s}). \end{array}$$
  The probability distribution $P_{t}\left(dy_{t}|y^{t-1},s\right)$ is then given by
  (A44) $$\begin{array}{cc} P_{t}^{\bar{P}}\left(dy_{t}|y^{t-1},s\right)= & \int P_{t}\left(dy_{t}|x_{t},s_{t}\right)P_{t}\left(dx_{t}|s_{t},y^{t-1},s\right)\\ \; & .P_{t}^{\bar{P}}\left(ds_{t}|y^{t-1},s\right),\;t=1,\ldots ,n,\;\text{by reconditioning and (A43)}. \end{array}$$
  The pay-off is the sum of conditional entropies $\sum_{t=1}^{n}H\left(Y_{t}|Y^{t-1},s\right)$, and the constraint is (300). By Definition 2, the state $S_{t}^{s},t=2,\ldots ,n$ is Markov, $P_{S_{t}^{s}|S^{s,t-1}}=P_{S_{t}^{s}|S_{t-1}^{s}},t=2,\ldots ,n$. By (A44) and the Markov property of $S_{t}^{s},t=1,\ldots ,n$, at each time *t*, the input distribution $P_{t}^{\bar{P}}\left(ds_{t}|y^{t-1},s\right)$ depends on $P_{j}\left(dx_{j}|s_{j},y^{j-1},s\right),j=1,\ldots ,t-1$ and not on ${\bar{P}}_{j}\left(dx_{j}|s^{j},y^{j-1},s\right),j=1,\ldots ,t-1$. By (301) and (303), $P_{t}\left(dx_{t}|s_{t},y^{t-1},s\right)={\bar{P}}_{t}^{M}\left(dx_{t}|s_{t},y^{t-1},s\right),t=1,\ldots ,n$. It is noted that (301) and (303) also follow from a slight variation of the derivation given in [8] (Theorem 1). By the maximum entropy principle of Gaussian distributions, it then follows that the distribution ${\bar{P}}_{t}^{M}\left(dx_{t}|s_{t},y^{t-1},s\right)$ is conditionally Gaussian, with linear conditional mean and nonrandom conditional covariance. Then, (304) follows by repeating Step 2 of the derivation of Theorem 3. This completes the derivation of all statements of part (a).
  (b,c). The statements follow from part (a), using the generalized Kalman filter, as in Theorem 3.

## References

[1] Kourtellaris C., Charalambous C.D., Sergey L. New Formulas for Ergodic Feedback Capacity of AGN Channels Driven by Stable and Unstable Autoregressive Noise. Proceedings of the IEEE International Symposium on Information Theory (ISIT).

[2] Louka S., Kourtellaris C., Charalambous C.D. Qualitative Analysis of Feedback Capacity of AGN Channels Driven by Stable and Unstable Autoregressive Moving Average. Proceedings of the 2021 IEEE Information Theory Workshop (ITW).

[3] Charalambous C.D., Kourtellaris C., Louka S. Sequential Characterization of Cover and Pombra Gaussian Feedback Capacity: Generalizations to MIMO Channels via a Sufficient Statistic. Proceedings of the 2021 IEEE Information Theory Workshop (ITW).

[4] Kim Y.H. (2010). Feedback Capacity of Stationary Gaussian Channels. IEEE Trans. Inf. Theory.

[5] Cover T., Pombra S. (1989). Gaussian feedback capacity. IEEE Trans. Inf. Theory.

[6] Derpich M.S., Ostergaard J. (2024). Comments on “Feedback Capacity of Stationary Gaussian Channels”. IEEE Trans. Inf. Theory.

[7] Gattami A. (2019). Feedback Capacity of Gaussian Channels Revisited. IEEE Trans. Inf. Theory.

[8] Yang S., Kavcic A., Tatikonda S. (2007). On Feedback Capacity of Power-Constrained Gaussian Noise Channels with Memory. Inf. Theory IEEE Trans. Theory.

[9] Ihara S. (2019). On the Feedback Capacity of the First-Order Moving Average Gaussian Channel. Jpn. J. Stat. Data Sci..

[10] Charalambous C.D., Kourtellaris C., Louka S. (2020). New Formulas of Feedback Capacity for AGN Channels with Memory: A Time-Domain Sufficient Statistic Approach. arXiv.

[11] Liu T., Han G. (2019). Feedback Capacity of Stationary Gaussian Channels Further Examined. IEEE Trans. Inf. Theory.

[12] Li C., Elia N. (2019). Youla coding and computation of Gaussian feedback capacity. IEEE Trans. Inf. Theory.

[13] Butman S. (1976). Linear Feedback Rate Bounds for Regressive Channels. IEEE Trans. Inf. Theory.

[14] Wolfowitz J. (1975). Signalling over a Gaussian Channel with Feedback and Autoregressive Noise. J. Appl. Probab..

[15] Ihara S. (1993). Information Theory for Continuous Systems.

[16] Caines P.E. (1988). Linear Stochastic Systems.

[17] Kailath T., Sayed A., Hassibi B. (2000). Linear Estimation.

[18] Gallager R.T. (1968). Information Theory and Reliable Communication.

[19] Butman S. (1969). A General Formulation of Linear Feedback Communications systems with Solutions. IEEE Trans. Inf. Theory.

[20] Ebert P.M. (1970). The capacity of the Gaussian channel with feedback. Bell Sys. Tech. J..

[21] Tienan J., Schalkwijk J.P.M. (1974). An upper bound to the capacity of bandlimited Gaussian autoregressive channel with noiseless feedback. IEEE Trans. Inf. Theory.

[22] Dembo A. (1989). On Gaussian Feedback Capacity. IEEE Trans. Inf. Theory.

[23] Ihara S., Yanaki K. (1989). Capacity of discrete-time Gaussian channels with and without feedback-II. Jpn. J. Appl. Math..

[24] Ozarow L. (1984). Upper Bounds on the Capacity of Gaussian Channel with Feedback. IEEE Trans. Inf. Theory.

[25] Ozarow L.H. (1984). Random coding for additive Gaussin channels with Feedback. IEEE Trans. Inf. Theory.

[26] Yanaki K. (1992). Necessary and sufficient conditions for the capacity of the discrete-time Gaussian channel to be increased by feedback. IEEE Trans. Inf. Theory.

[27] Yanaki K. (1994). An upper bound on the discrete-time Gaussian channel with feedback-II. IEEE Trans. Inf. Theory.

[28] Chen H.W., Yanaki K. (1999). Refiniements of the half-bit and factor-of-two bounds for capacity in Gaussian channels with feedback. IEEE Trans. Inf. Theory.

[29] Chen H.W., Yanaki K. (2000). Upper bounds on the capacity of discrete-time blockwise white Gaussian channels with feedback. IEEE Trans. Inf. Theory.

[30] Gallager R.G., Nakiboglu B. (2010). Variations on a Theme by Schalkwijk and Kailath. IEEE Trans. Inf. Theory.

[31] Ordentlich E. A Class of Optimal Coding Schmes for Moving Average Additive Gaussian Noise Channels with Feedback. Proceedings of the IEEE International Symposium on Information Theory Proceedings (ISIT).

[32] Tatikonda S., Mitter S. (2009). The Capacity of Channels with Feedback. IEEE Trans. Inf. Theory.

[33] Liu T., Han G. The ARMAk Gaussian Feedback Capacity. Proceedings of the IEEE International Symposium on Information Theory Proceedings (ISIT).

[34] Kim Y.H. (2006). Feedback capacity of the first-order moving average Gaussian channel. IEEE Trans. Inf. Theory.

[35] Kourtellaris C., Charalambous C.D., Sergey L. From Feedback Capacity to Tight Achievable Bounds without Feedback for AGN Channels with Stable and Unstable Autoregressive Noise. Proceedings of the IEEE International Symposium on Information Theory (ISIT).

[36] Kourtellaris C., Charalambous C.D. (2018). Information Structures of Capacity Achieving Distributions for Feedback Channels with Memory and Transmission Cost: Stochastic Optimal Control & Variational Equalities. IEEE Trans. Inf. Theory.

[37] Charalambous C.D., Kourtellaris C., Loyka S. (2018). Capacity Achieving Distributions and Separation Principle for Feedback Gaussian Channels with Memory: The LQG Theory of Directed Information. IEEE Trans. Inf. Theory.

[38] Sabag O., Kostina V., Hassibi B. Feedback Capacity of MIMO Gaussian Channels. Proceedings of the IEEE International Symposium on Information Theory (ISIT).

[39] Kumar P.R., Varaiya P. (1986). Stochastic Systems: Estimation, Identification, and Adaptive Control.

[40] van Schuppen J.H. (2021). Control and System Theory of Discrete-Time Stochastic Systems.

[41] Charalambous C.D., Louka S. A Riccati-Lyapunov Approach to Nonfeedback Capacity of MIMO Gaussian Channels Driven by Stable and Unstable Noise. Proceedings of the 2022 IEEE Information Theory Workshop (ITW).

